# Towards a unified model of aneuploid karyotype dynamics

**DOI:** 10.1371/journal.pgen.1012210

**Published:** 2026-06-18

**Authors:** Mathieu Hénault, Lisa M. Wood, Lydia R. Heasley

**Affiliations:** Department of Biochemistry and Molecular Genetics, University of Colorado Anschutz Medical Campus, Aurora, Colorado, United States of America; Dartmouth College Geisel School of Medicine, UNITED STATES OF AMERICA

## Abstract

Aneuploidies—whole-chromosome copy number imbalances arising from nondisjunction—underlie numerous congenital and somatic disorders, but unlike many other disease-causing variants, they can revert back to euploidy through subsequent errors of the same type. The extent to which this inherent plasticity impacts the stability and persistence of aneuploid karyotypes in populations remains poorly understood, a gap in knowledge that continues to limit our understanding of aneuploidy-driven disease incidence, penetrance, and progression. To assess how reversion shapes aneuploid population dynamics, we developed a budding yeast system to systematically measure the rates at which aneuploidies arise and revert and quantify the relative fitness differences between these karyotypic states. We integrated these data into a computational framework encompassing the broad physiological range of aneuploid karyotype dynamics captured in our experiments. The resulting models reveal that canonical reversion (*i.e.*, subsequent secondary nondisjunction) occurs rarely, conferring a negligible effect on the population dynamics of most chromosomal aneuploidies. However, our models also identified that the reversion dynamics of some chromosomes—those displaying extremely high apparent rates of reversion—were more consistent with a coupled mutational process involving a transient aneuploid state. Whole-genome sequencing and live-cell microscopy demonstrates one such mechanism is facilitated by unresolved intermolecular linkages that disrupt chromosome segregation, leading to chromosome breakage and recombination-mediated repair over subsequent cell divisions. Collectively, this work advances a model of aneuploid population genetics and expands our perspective of the diverse, and chromosome-specific, mutational mechanisms shaping genome architecture.

## Author summary

Aneuploidies are important sources of genetic diversity that alter the copy number of entire chromosomes. These massive alterations in genome content can be adaptive or deleterious depending on biological context, yet predicting their functional consequences requires a better understanding of how frequently they arise and how stably they are propagated. Here, we measured the rates, stability, and fitness-costs of chromosome loss events for each chromosome in the budding yeast, *Saccharomyces cerevisiae*. Using these empirical data, we developed an integrated framework with which to explore the determinants that shape the landscape of aneuploid karyotypes in populations. We find that these parameters vary broadly across chromosomes, with a subset displaying instability dynamics not captured by standard population genetic models. Investigation of these outliers revealed that persistent physical linkages between chromosomes—which cause breakage during cell division and subsequent copy number variations generated via homologous repair of chromosome fragments—represent an additional source of instability that varies in incidence across chromosomes.

## Introduction

The functional relevance of any novel genetic variation depends not only on the phenotypic effect it induces, but on its capacity to persist in the population. For most variants, persistence is primarily determined by the rates at which they arise (μ) and their relative fitness (ω). Yet the variants most likely to exert the strongest phenotypic effects—whole-chromosome copy number variations (*i.e.*, aneuploidies)—are additionally constrained by a third factor: reversion. Arising through mitotic or meiotic nondisjunction events, aneuploidies can impart profound physiological changes to cells by simultaneously altering the dosage of hundreds to thousands of genes. Aneuploidies underlie numerous pathological states and adaptive processes, ranging from constitutional conditions like Down syndrome [1], to tumorigenesis [2,3], to the emergence of antimicrobial drug resistance [4]. But these aneuploid states are inherently unstable in dividing populations; each subsequent mitotic cell division carries an intrinsic risk of nondisjunction, providing aneuploid cells with opportunities to revert to euploidy or adopt new aneuploid karyotypes [5–11]. As such, and irrespective of any advantageous effects conferred by an aneuploid state, the associated phenotypes are ultimately only as stable as the underlying aneuploid genotype.

Aneuploidy reversion has been documented in numerous experimental systems [7,9,12,13] and is generally described by a two-step sequential mutational framework predicated on the assumptions that a cell must first become aneuploid (step 1, A) before reverting (step 2, R), and that both aneuploid formation and reversion occur stochastically. Within this framework, models differ in their assertions about the likelihood of reversion. Since A and R both constitute nondisjunction of the same chromosome, the probability of R occurring could be expected to equal that of A (*i.e.,* μ^A^ = μ^R^), referred to here as the equal-likelihood model. By contrast, if A alters basal genome stability [14,15], the probability of subsequent nondisjunction events including R occurring may be increased or decreased (*i.e.,* μ^A^ ≠ μ^R^), referred to here as the unequal-likelihood model. Yet, which of these models most accurately captures observed dynamics of reversion, and the extent to which this relationship shapes population-level karyotypic diversity remains poorly understood.

Empirically disentangling the effects of μ^A^, μ^R^, and ω on the observed frequency of an aneuploid karyotype has proven particularly challenging, since each determinant influences estimation of the others. Even studies using experimental approaches like mutation accumulation assays and fluctuation tests, which minimize the influence of ω on mutation rate estimates as compared to adaptive experimental evolution-type studies, have not explicitly accounted for the effects of ω and μ^R^ on the aneuploid cell counts observed, potentially confounding the derived estimates of μ^A^ [12,15–17]. Likewise, since the relative fitness (ω) of aneuploid cells is often lower than euploids, estimates of aneuploidy-associated fitness effects may be substantially distorted if the emergence of revertants (μ^R^) is not considered [18–24]. Most recently, μ^R^ for three chromosomal aneuploidies in yeast was estimated with a system that accounted for fitness differences between the aneuploid and revertant states, but precluded parallel estimation of μ^A^ for the aneuploidies tested [9].

As it stands, a unified model incorporating the inherent ephemerality of aneuploid karyotypes into the complex interplay of factors governing their persistence in populations has not been established. Thus, our ability to anticipate the occurrence, stability, and functional implications of these important mutations remains fundamentally limited. To address this gap in knowledge, we systematically investigated aneuploidy reversion with an experimental system capable of integrating all major determinants of aneuploid frequency (μ^A^, ω, μ^R^). This approach allowed us to construct a comprehensive, quantitative model of aneuploid population dynamics and in doing so to identify and characterize a previously unidentified, non-canonical mutational mechanism that directly links the nondisjunction and rapid reversion of specific chromosomal aneuploidies.

## Results

### A genetic system to investigate aneuploidy dynamics in populations

Although chromosome loss is typically lethal in haploids, it can be tolerated in diploids [12,15,25,26]. The resulting aneuploid state—called monosomy—can produce diverse effects on cellular physiology and fitness [2,10–12,14,15], and once established, can persist for many generations. However, monosomic cells can also revert to euploidy by acquiring a second copy of the remaining homologous chromosome, generating a genotype known as uniparental disomy (UPD) or whole chromosome loss of heterozygosity [12,13,27–29]. Although UPDs restore a balanced chromosomal copy number, they result in whole-chromosome homozygosis and are, themselves, associated with numerous recessive genetic disorders [13,15,27,30]. Given the important physiological implications of both monosomy and UPD, we focused our systematic analysis of aneuploidy frequency on this aneuploid-reversion pair using a tractable budding yeast (*Saccharomyces cerevisiae*) system.

**Fig 1:**
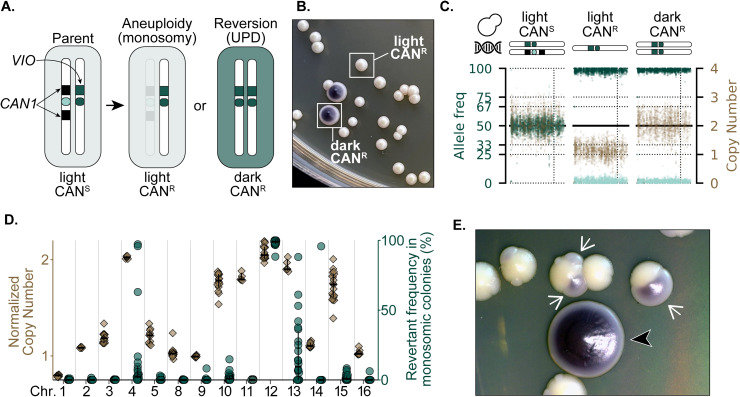
A genetic reporter system clarifies variations in reversion frequency across chromosomes. **A.** A schematic describing the genotypic and phenotypic outcomes reported by the *CAN1*-VIO selectable yeast system. **B.** A representative image of light and dark CAN^R^ colonies derived from the Chr10 strain. **C.** WGS data signatures associated with the parental (diploid) strain, light CAN^R^ (monosomic) colonies, and dark CAN^R^ (revertant UPD) colonies. Each dot denotes a heterozygous allelic position; colors denote normalized depth of coverage (brown), and the allele frequencies of the *CAN1*-marked chromosome (light green) and *VIO*-marked chromosome (dark green). **D.** Per-chromosome quantifications of the normalized copy number estimates inferred from WGS analysis of light CAN^R^ clones (brown diamonds) and the revertant frequency derived from the monosome replating tests (green circles). **E.** A representative image of the sectoring phenotype displayed by the CAN^R^ derivatives of the chromosome strains that display higher levels of revertant mosaicism, in this case, Chr10. Black arrowhead denotes a revertant colony identified at day 2 of the test, white arrowheads denote sectors that emerged within light colonies later in the experimental timecourse.

To facilitate selective recovery and high-resolution mutational analyses of cells harboring monosomy or revertant UPD of each of the 16 yeast chromosomes, heterozygous diploid yeast strains were engineered as follows: for each pair of homologous chromosomes, two *CAN1* gene cassettes were inserted at loci on each arm of one homolog and a violacein pigment operon [31] was inserted into the other homolog at a position allelic to one of the *CAN1* cassettes (Fig 1A). Loss of *CAN1* expression confers resistance to the toxic arginine analog canavanine (CAN^R^), and this double-cassette architecture enriches for recovery of CAN^R^ clones arising from complete chromosome loss while reducing those that result from copy-neutral loss of heterozygosity (LOH), segmental deletion, or point mutation [15,26]. When populations of these parental strains were pregrown in nonselective conditions and then plated onto canavanine-containing media, spontaneously arising CAN^R^ colonies developed and resolved into two major phenotypic classes—light purple (parental-like) or dark purple—reflecting the copy number difference of the pigment operon between monosomic and revertant UPD clones. In most backgrounds, dark CAN^R^ colonies were also larger, a phenotype consistent with euploid revertants having higher predicted ω than monosomes (Fig 1B) [9].

The high degree of heterozygosity existing between the homologs in these strains enabled us to use whole-genome sequencing (WGS) of independent light and dark CANᴿ clones to confirm the accuracy of this selectable system (S1 Fig and Table B in S1 Appendix). Light CANᴿ clones predominantly showed a monosomic signature of the expected chromosome—evidenced by whole-chromosome homozygosity and reduced read coverage—and dark CANᴿ clones primarily exhibited a UPD signature—evidenced by whole-chromosome homozygosity and diploid-level coverage (Fig 1C). This analysis also revealed recurrent, chromosome-specific, reversion patterns: whereas the affected chromosome in monosomic clones of Chr1, Chr2, Chr3, Chr5, Chr8, Chr9, Chr14, and Chr16 usually had the expected normalized copy number near 1 copy, all monosomic clones of Chr4, Chr10, Chr11, Chr12, Chr13, and Chr15 had a normalized copy number nearer to 2 copies (Fig 1D, brown diamonds). This suggested that, despite displaying the light colony color phenotype, the clones of this latter group were mosaic, with sizable revertant subpopulations already present at the time of sequencing. We validated the variable revertant mosaicism inferred from WGS by directly measuring the frequency of revertant cells in monosomic populations on a per-chromosome basis. Light monosomic CAN^R^ colonies isolated from independent canavanine plates were resuspended completely in water and diluted to single-cell density on rich media plates. After 2 days, dark revertant colonies were counted; total colony counts were quantified after 2 additional days, to accommodate the slower growth of monosomes (Fig 1E). Sequencing confirmed that the dark colonies recovered from these monosome-replating tests harbored the predicted revertant UPD genotype. In agreement with the WGS analysis, revertants were absent or very rare in populations of Chr1, Chr2, Chr3, Chr5, Chr8, Chr9, Chr10, Chr14, Chr15, and Chr16, but prevalent in populations of Chr4, Chr12, and Chr13 (Fig 1D, green circles). In both assessments of revertant frequency, Chr12 was a striking outlier: every sequenced light CAN^R^ clone showed a penetrant signature of UPD, and every replated population was almost entirely fixed by revertants (average revertant frequency, 99%). At the experimental endpoint of these replating tests, we noted variations in colony appearance further reflecting the diverse reversion dynamics of different chromosomes: though dark colonies quantified after 2 days were large and fully purple (Fig 1E, black arrowhead), many smaller monosomic colonies developed dark-purple sectors, indicating that reversions had occurred post-plating (Fig 1E, white arrowheads). This sectored morphology was most pronounced in CAN^R^ colonies from the Chr4, Chr10, Chr13, and Chr15 strains.

### A per-chromosome assessment of the fitness costs associated with monosomy

**Fig 2:**
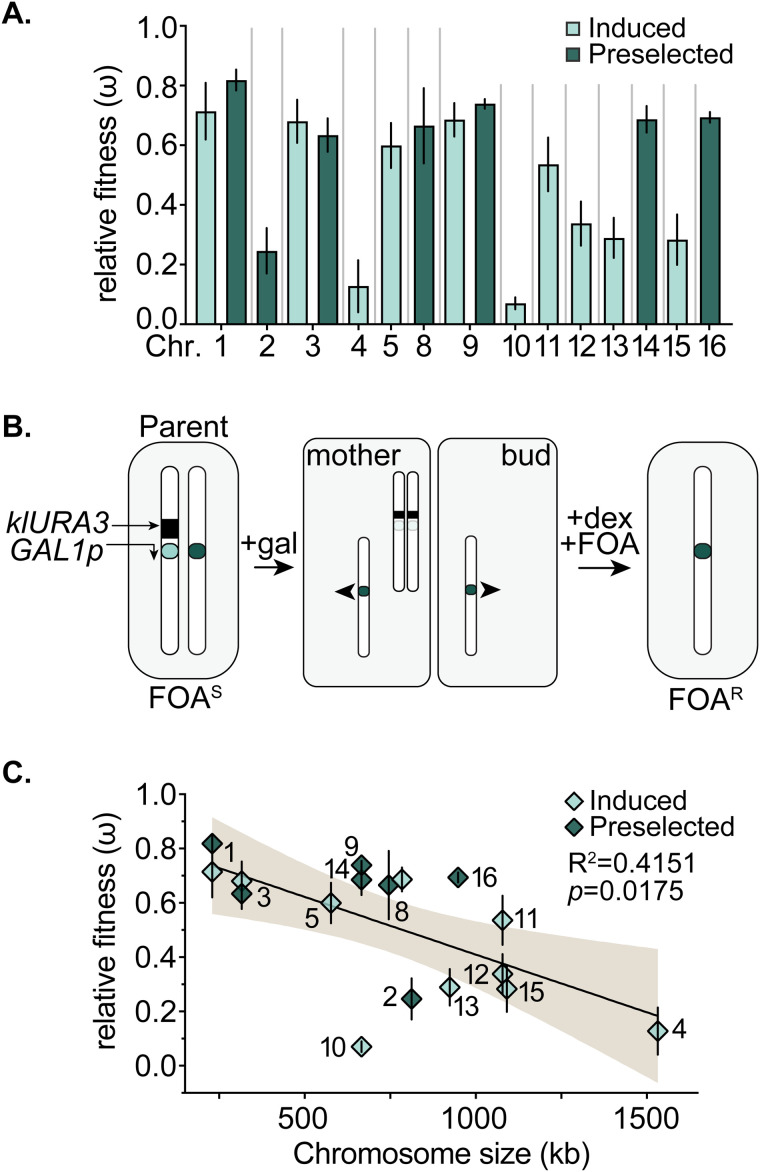
Fitness costs associated with monosomy vary markedly across chromosomes. **A.** The ω values derived from the induced (light green) and preselected (dark green) growth assays for each chromosome **B.** A schematic describing the *URA3-GAL1p-CEN* monosome generation and enrichment approach. **C.** The deduced ω values measured from the induced (light green) and preselected (dark green) growth assays for each chromosome plotted according to chromosome size. Brown curve depicts the 95% confidence intervals produced from the simple linear regression of ω vs. chromosome size.

The frequency of revertants in a monosome-derived population is determined by both the rate of reversion (μ^R^) and the relative growth rates (ω) of monosomic and revertant cells. For example, the high frequency of revertants in Chr4 and Chr12 monosomic populations could reflect a high rate of reversion or the high relative fitness of revertants compared to their slower-growing monosomic predecessors. Conversely, the low frequency of revertants in Chr2 monosomic populations could result from a low rate of reversion or the similar fitness of monosomic and revertant cells. To distinguish between these possibilities on a per-chromosome basis, we measured ω of monosomic and euploid cells using two orthogonal liquid growth assays. In the first assay, light CAN^R^ populations were inoculated in replicate microwell rich media cultures and growth kinetics were assessed by optical density (OD). Because revertant subpopulations would distort estimates of ω, this approach was restricted to monosomes that consistently exhibited undetectable revertant frequencies by both WGS and monosome replating tests (Fig 2A, preselected monosomes). For the second assay, we engineered a new suite of strains that enabled measurement of ω immediately after monosomic cell formation. Hemizygous insertions of the inducible *GAL1* promoter (*GAL1p*) were introduced adjacent to one centromere of each respective homologous pair (*GAL1p-CEN)*(Fig 2B) [13,32]. This cassette also encoded a *URA3* gene, rendering cells prototrophic for uracil (URA+) but sensitive to the drug 5-FOA (FOA^S^) [33]. In dextrose-containing media, *GAL1*p is repressed, allowing the centromere to function normally to facilitate accurate spindle-mediated disjunction of the sister chromatid pair in mitosis. Induction of *GAL1p* in galactose-containing media disrupts centromere activity via constitutive transcription, specifically impairing kinetochore assembly and spindle-mediated disjunction of the *URA3-GAL1p*-*CEN* chromatid pair [34]. Consequently, the mother cell retains both copies, becoming a trisomic URA + /FOA^S^ cell and producing a monosomic URA-/FOA^R^ bud cell; the latter cell type can be enriched by selection in 5-FOA containing-media (Fig 2B). To eliminate pre-existing FOA^R^ cells that could confound fitness measurements, populations were pregrown in media lacking uracil prior to induction with galactose. Induced cultures were then transferred into microwell cultures with dextrose- and 5-FOA-containing media to counterselect against the trisomic population and any residual uninduced disomic cells. The growth kinetics of the FOA^R^ subpopulation generated was monitored by OD. For both assays, OD measurements were used to determine the average maximum slope of the logarithmic growth phase for each monosomic replicate and a euploid control, and ω was defined as the quotient of these values, respectively.

Most monosomes, even those displaying severe growth defects, showed consistent cross-replicate growth kinetics throughout the experiment and uniform cell distributions at the bottom of the wells at the experimental endpoint (S2 Fig). In contrast, replicate cultures of monosomes of Chr4, Chr10, Chr12, Chr13, and Chr15 initially grew similarly, but became variable at later timepoints. Cell distributions within these wells were heterogeneous at the experiment’s end, reflecting a mixture of slow-growing and faster-growing subpopulations. Suspecting that these subpopulations consisted of revertants that had emerged soon after induction of monosomy and subsequently swept the population, we limited quantification of ω to the first 3–4 divisions of growth (S2 Fig, white area). Nonetheless, we cannot dismiss the possibility that high frequency reversion events compromised the accuracy of ω calculated for these specific chromosomes.

In both assays, monosomy universally reduced relative fitness, though the costs incurred depended greatly on the chromosome affected (Fig 2A). These findings provide additional support for the widely accepted model that aneuploidies disrupt essential biosynthetic and metabolic pathways through gene dosage imbalances [4,18,19,22–24,35,36]. Consistent with the work of others, we identified a statistically significant correlation between ω and chromosome size (Fig 2C)(R^2^ = 0.42, p = 0.0175). We did not investigate the biological basis of this relationship in the present study, since it has been reported elsewhere [22]. The results of these experiments provided insight into the reversion patterns observed in our earlier experiments by establishing two central principles: 1) fitness influences aneuploid frequency, and 2) reversion probability differs across chromosomes. For example, monosomy of Chr2 confers a severe fitness defect (ω = 0.246) yet replated monosomic clones yielded almost no revertants (Fig 1D), indicating that μ^R^ is very low. In contrast, monosomes of Chr4, Chr12, and Chr13—despite displaying similarly severe fitness costs—produced high frequencies of revertants, indicating that they revert at markedly higher rates.

### Establishing an integrated framework of aneuploid karyotype population dynamics

**Fig 3:**
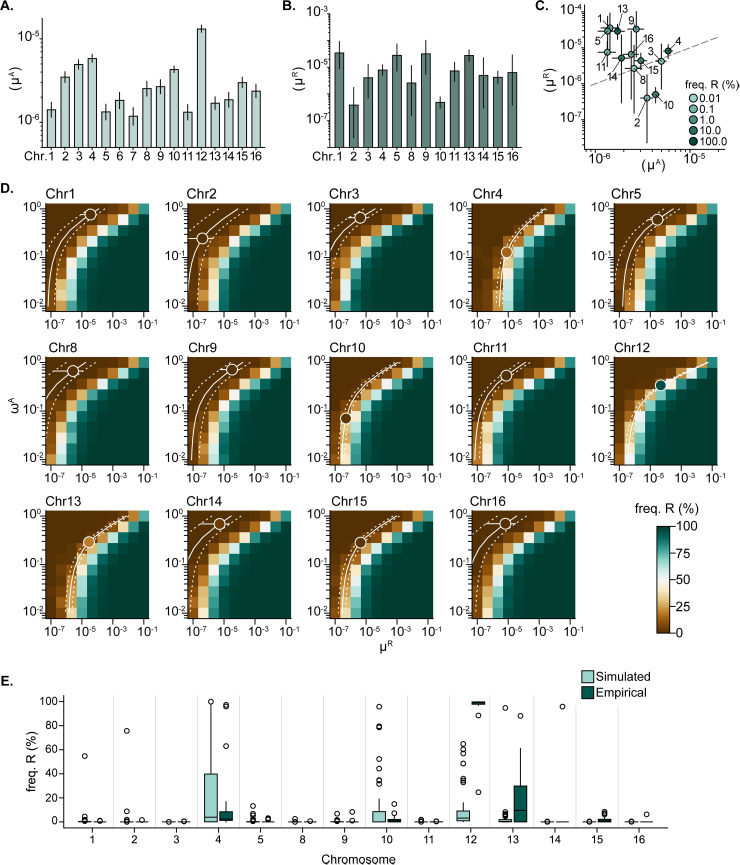
Rates of aneuploidy and reversion generally conform to an unequal likelihood model. **A.** Per-chromosome rates of monosomy (μ^A^) estimated from light CAN^R^ colony counts. **B.** Per-chromosome rates of reversion (μ^R^) estimated from the monosome replating test revertant count data. **C.** Comparison between μ^A^ and μ^R^. Horizontal and vertical bars indicate 95% confidence intervals of rate estimates. Dot color represents the average extant frequency of revertant cells in the replating tests from which μ^R^ values were computed. Dotted diagonal represents identity. **D.** Heatmaps showing the average extant frequency of revertant cells in 50 replicate simulated population expansions for each combination of input parameter values (μ^R^ and ω^A^). For each chromosome, simulated final population sizes were set to the average from the empirical replating tests. Outlined circles represent the empirical values for μ^R^ and ω^A^, and the circle fill color represents the empirically observed mean frequency of revertant cells. Horizontal lines denote the 95% confidence interval of the empirical μ^R^. Solid and dotted curves denote the estimated 95% confidence interval for μ^R^, respectively, fitted on empirical colony counts across the variable range of ω shown on the Y axis, representing the value computed for μ^R^ if there were an experimental error in the estimate of ω^A^. **E.** Comparison of the distributions of revertant frequencies across replicates of the simulated and empirical monosome replating tests. Individual circles denote outliers. Data are also presented as cumulative distribution curves in S6A Fig.

We proceeded to estimate per-chromosome rates of monosome formation (μ^A^) and reversion (μ^R^) using fluctuation assays [37,38]. For each CAN1-VIO strain, replicate populations were seeded into microwells containing rich media and expanded to saturation. The culture was then fully mixed and a fraction of each replicate was plated on canavanine-containing selective media to quantify CAN^R^ colonies, and a diluted fraction was plated on non-selective media to estimate total population size. Dark CAN^R^ revertants and light CAN^R^ monosomes were counted and scored after 2 and 4 days, respectively. To estimate mutation rates from the extant colony counts while also accounting for relative fitness differences between monosomic and euploid cells (both parental or revertant), we used the maximum likelihood estimator (MLE) program mlemur [39]. Light CAN^R^ colony counts were used to estimate μ^A^ (Fig 3A). The resulting ω-adjusted μ^A^ values varied >10-fold—ranging from 1.20✕10^-6^ cell^-1^ gen^-1^ (Chr7) to 1.33✕10^-5^ cell^-1^ gen^-1^ (Chr12) (Fig 3A)—suggesting that yeast chromosomes are differentially subjected to processes which influence their individual rates of nondisjunction. Similar values were also obtained from orthologous fluctuation tests using populations pregrown on solid media (Tables G and H in S1 Appendix). Although chromosome size has been correlated with nondisjunction frequency in humans [40], no significant relationships between μ^A^ and chromosome size, chromosome arm length, genic-, or non-genic element content were identified (S3 Fig) [22].

Estimating μ^R^ required additional consideration. Since a cell must first become monosomic before reverting to form a dark CAN^R^ colony, the rate estimated from dark CANᴿ colony counts would typically be interpreted as the compound rate (μ^C^) of these two sequential mutations:


$$\begin{array}{c} \mu^{\text{C}}\;=\;\mu^{\text{A}}\;\times \;\mu^{\text{R}} \end{array}$$ (1)

Additionally, because the populations subjected to the canavanine fluctuation tests were founded by diploid cells, dark CANᴿ revertant colonies could arise from two parallel mechanisms: reversion from monosomy or from trisomy (S4 Fig and S2 Appendix). Monosomic and trisomic karyotypic states form as reciprocal products of the same nondisjunction event; therefore, the rate of trisome formation is equivalent to μ^A^. However, neither the relative fitness of trisomes (ω^T^) nor their rates of reversion (μ^T^)—both of which would influence the frequency of extant dark CAN^R^ colonies—could be directly measured with this selectable-genetic system, precluding accurate estimation of μ^R^. To circumvent these confounding issues, we instead estimated μ^R^ using the revertant colony counts quantified from the monosome replating tests. Because the populations subjected to the replating tests started with monosomic founders, the dynamics of reversion could be directly assessed without having to account for the parallel dynamics of trisomy. The resultant μ^R^ values varied 89-fold between chromosomes (4.02✕10^-7^(Chr2) to 3.58✕10^-5^ (Chr1)) (Fig 3B). For most chromosomes, reversion probability best fits an unequal likelihood model (μ^A^ ≠ μ^R^): Chr2 and Chr10 had lower μ^R^ than μ^A^ (8.75-fold and 8.63-fold respectively), while Chr1, Chr9, Chr11, Chr13, Chr14, and Chr16 had higher μ^R^ than μ^A^ (2.74-fold to 25.03-fold). Only Chr3, Chr4, Chr8, and Chr15 show similar μ^A^ and μ^R^ values more indicative of an equal likelihood model of reversion (Fig 3C). In general, the consistently low values of μ^R^ indicate that chromosome loss does not substantially perturb genome stability in this organism, clarifying a central facet of aneuploid population dynamics: across the physiologically relevant range of μ^A^, μ^R^, and ω values measured in these experiments, reversion is a minor determinant of monosomic karyotype stability in populations.

Estimation of μ^R^ for Chr12 was confounded by the near-complete sweep of revertants in all replicate monosomic populations. Revertant fixation violates an integral requirement of fluctuation assay-derived rate estimation: mutants must be rare enough that independent mutant lineages remain distinguishable within each population. This high revertant frequency permits at least two alternative interpretations: reversion from monosomy could occur through a single mechanism operating at an exceptionally high rate, or reflect the combined output of multiple parallel mechanisms. To distinguish between these possibilities, we developed fflucsim, a forward simulator of population mutational dynamics. fflucsim performs *in silico* fluctuation tests parameterized by user-specified values for all relevant variables (namely μ^A^, μ^R^, μ^T^, ω^A^, ω^T^, and final population size) and explicitly tracks mutation events, mutant frequencies, and population genealogies. We validated that fflucsim accurately recapitulates standard mutational models by simulating population expansion across a broad range of mutation rate and relative fitness values, then benchmarking the known input rates against those estimated from output mutant counts using rSalvador [41] (S5 Fig).

We used fflucsim to investigate whether the frequency of revertants observed in the monosome replating tests reflected one or multiple mechanisms. If revertants had emerged via a single mechanism, then fflucsim models parameterized solely by ω and μ^R^ should be sufficient to reproduce the observed frequencies. If multiple reversion mechanisms had contributed to the emergence of revertants, even models permitting very high μ^R^ values would fail to recapitulate the observed revertant abundance. We simulated replicate monosomic population expansions across the experimentally derived parameter space of ω and μ^R^ for each chromosome tested, then directly compared the simulated and observed revertant frequencies across the full range of input values. For most chromosomes (Chr1, Chr2, Chr3, Chr5, Chr8, Chr9, Chr10, Chr11, Chr14, Chr15, and Chr16), simulations parameterized with their empirically-derived μ^R^ and ω values produced revertant count distributions that closely matched the observed data; any deviations were fully attributable to minor errors in our empirical estimates of ω or μ^R^ (Fig 3D). In contrast, no combination of ω and μ^R^—even μ^R^ values close to 1, which would constitute an essentially deterministic event—was sufficient to recapitulate the high frequency of revertants observed in the replated populations of Chr12 monosomes. In addition, the observed revertant frequency distributions for Chr4, Chr10, and Chr13 also differed from the simulated results (Fig 3E and S6 Fig), indicating that the reversion dynamics of Chr4, Chr10, Chr12, and Chr13 do not conform to the predictions of standard mutational models. Instead, they better support the interpretation that for these chromosomes, reversion occurs through multiple parallel mutational routes.

Before investigating the molecular basis of these predicted alternative reversion pathways, we re-examined the canavanine fluctuation test results to evaluate the contribution of monosomic cell-derived reversion to the observed frequencies of dark CAN^R^ colonies. If dark CAN^R^ colonies arise solely from the reversion of monosomic and trisomic cells, then the compound rate of formation (μ^C^) would be the sum of rates describing both two-step mutational processes (S4 Fig):


$$\begin{array}{c} \mu^{\text{C}}\;=\;{(\mu }^{\text{A}}\;\times \;\mu^{\text{R}})\;+(\mu^{\text{A}}\;\times \;\frac{\text{1}}{\text{3}}\mu^{\text{T}}) \end{array}$$ (2)

**Fig 4:**
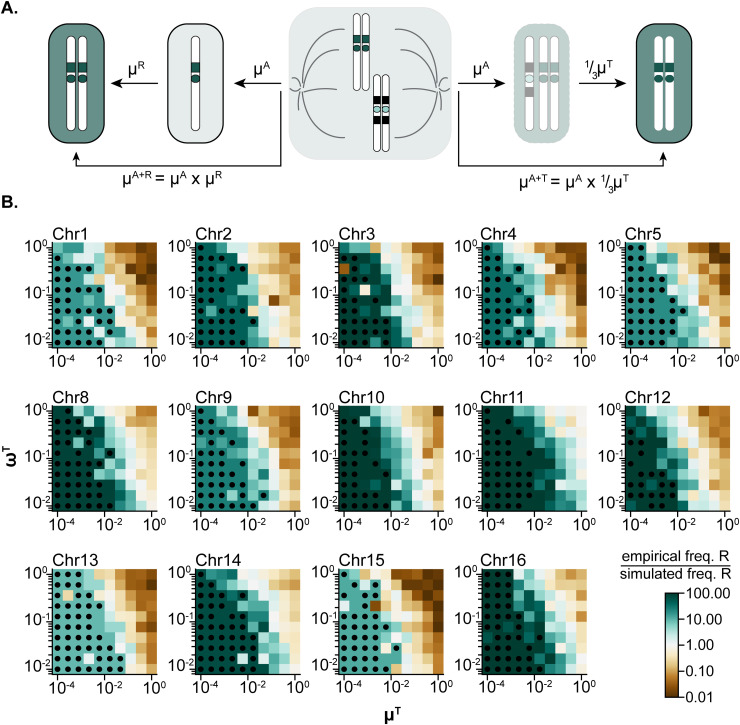
Empirical revertant frequencies are not fully described by reversion from intermediate aneuploid states. **A.** Trisomic cells are produced as reciprocal products of the same aneuploidy event. Monosomic cells can revert to a disomic karyotype at rate μ^R^, while trisomic cells revert to a disomic karyotype at rate μ^T^. **B.** Heatmaps show the fits of the revertant CAN^R^ cell frequencies derived from the experimental and simulated fluctuation assays. Cell color corresponds to the ratio of the mean revertant CAN^R^ cell frequency in 50 replicate simulated populations and the empirical frequency for each combination of input parameter values (μ^T^ and ω^T^); see heatmap legend at bottom right. The empirical estimates were specified for the parameters: μ^A^, μ^R^ and ω^A^. For each chromosome, simulated final population sizes were set to the average from the CAN^R^ fluctuation assays. Dots mark parameter combinations for which no viable revertant cell was produced across all 50 simulated replicates, and were substituted by a lower bound frequency corresponding to a single viable revertant cell in a single replicate.

With chromosome-specific μ^A^ and μ^R^ values determined, we inferred the per-chromosome compound rates of monosomic cell-derived reversion (μ^A+R^ = μ^A^ ✕ μ^R^) and compared the resulting values to the rates estimated directly from the dark CAN^R^ colony counts (μ^C^) (Fig 4A). The resulting μ^A+R^ values were ~2–5 orders of magnitude lower than μ^C^, indicating that reversion from monosomy had contributed minimally to the occurrence of dark CAN^R^ colonies in the tested populations. This result suggested that the majority of observed revertants derived from trisomic cells, reflecting either their increased relative fitness (ω^T^) or higher reversion rates (μ^T^). Higher ω^T^ values would result in greater expansion of trisomic subpopulations, thereby increasing the opportunity for reversion events to occur. Alternatively, if trisomic karyotypes are more genetically unstable, reversion could occur at higher rates than in monosomic cells (μ^T^ ≫ μ^R^). To explore these possibilities quantitatively, we again used fflucsim, this time simulating the expansion of populations founded by diploid ancestors. These simulations incorporated 5 parameters: empirically determined values of ω^A^, μ^A^ and μ^R^, and a broad range of hypothetical values of ω^T^ and μ^T^. We compared the frequency of revertant CAN^R^ cells produced by these simulations to the actual values observed in the CAN^R^ fluctuation tests (Fig 4B). These results demonstrate that for most chromosomes, μ^T^ values in the range of 10^-3^-10^-1^ cell^-1^ gen^-1^ would be necessary to produce frequencies of dark CAN^R^ cells compatible with our experimental results (Fig 4B, white cells). However, the limited experimental data available indicate that trisomic cells are less fit than euploids (ω^T^ < 1) [16,24], meaning that μ^T^ values approaching 1 would often be required to reconcile our observations with model outputs. To our knowledge, such values of μ^T^ would be orders of magnitude higher than any aneuploidy rate yet reported and incompatible with previous observations demonstrating the prevalence and long-term stability of trisomic karyotypes in wild populations of yeast [12,20,25,42]. Instead, these results provide additional support for the existence of one or more alternative mechanisms that generate revertant UPD karyotypes. Crucially, both our experimental data and forward simulations indicate that these mechanisms 1) need not proceed through a stable aneuploid intermediate (monosomic or trisomic) and 2) may operate at effective rates or with mutational dynamics which render them poorly described by the constant-rate assumptions underlying standard mutational models.

### Alternative mutational mechanisms facilitate generation of revertant karyotypes

**Fig 5:**
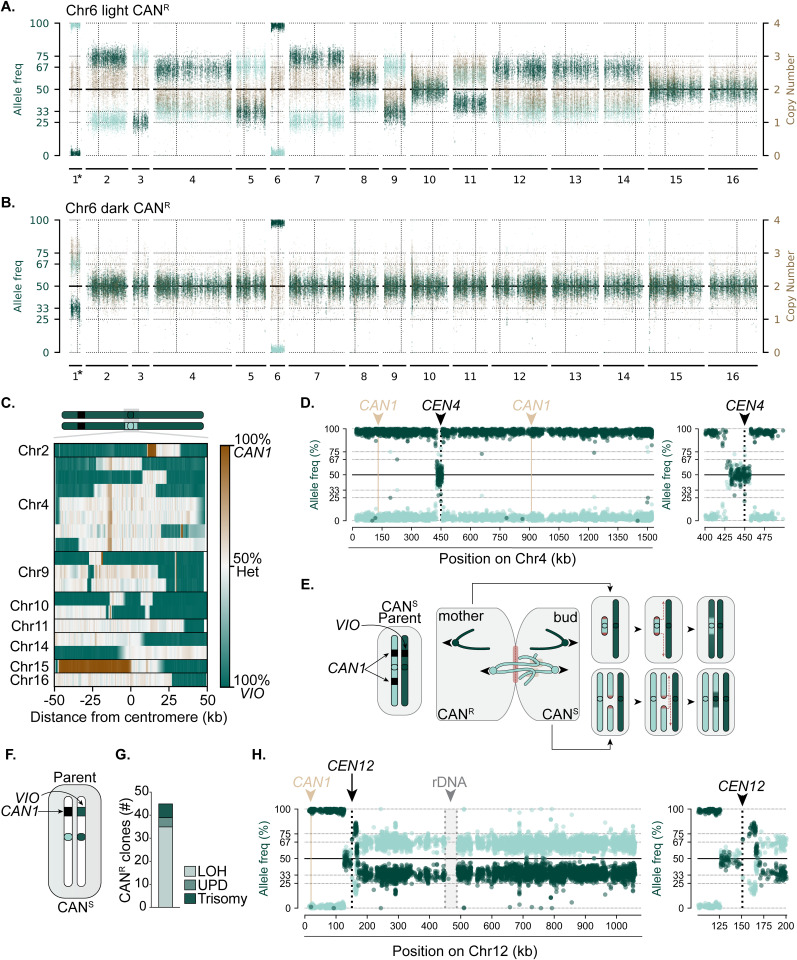
Disomic revertant karyotypes develop independent of a stable aneuploid intermediate state. **A. and B.** Representative WGS mappings of light and dark CAN^R^ clones derived from the Chr6 *CAN1*-VIO strain. Each dot denotes a heterozygous allelic position; colors denote normalized depth of coverage (brown), and the allele frequencies of the *CAN1*-marked chromosome (light green) and *VIO*-marked chromosome (dark green). Vertical dashed lines delineate centromeric positions. Trisomy of Chr1, denoted with the asterisk, is a preexisting aneuploidy present in the parental Chr6. **C.** A heatmap of allele frequency at each parental heterozygous position to illustrate the pericentromere-biased breakpoint positions observed in recombinant dark CAN^R^ clones. **D.** A representative Chr4-specific WGS mapping of a dark CAN^R^ colony recovered from the Chr4 *CAN1-*VIO strain displaying the characteristic pericentromeric breakpoints and heterozygosity. Each dot denotes a heterozygous position; color denotes the *CAN1*-marked chromosome (light green) and VIO-marked chromosome (dark green). **E.** A schematic illustrating the predicted mechanism and outcomes of a sister-sister intermolecular linkage mediated missegregation event. **F.** A schematic of the single *CAN1*-VIO cassette Chr12 strain. **G.** Quantification of the karyotypic spectra harbored by CAN^R^ clones derived from the single-cassette strain. **H.** Chr12-specific whole-chromosome or centromere-proximal WGS mappings of a recovered trisomic CAN^R^ colony.

To better understand the alternative reversion mechanisms implicated by our rate analyses, we performed a focused molecular characterization of reversion events guided by several observations from the WGS analysis of light and dark CAN^R^ clones. Unlike most light CAN^R^ colonies—in which only the copy number of the expected chromosome was affected—each clone from the Chr6 and Chr7 strains carried 1–13 additional unselected karyotypic alterations, many of which were mosaic within the sequenced population (Fig 5A and Table B in S1 Appendix). This precluded accurate assessments of both monosome fitness and reversion dynamics, and instead typified a mutational signature called chromosomal instability (CIN)—a mutator phenotype defined by the constitutive, high frequency accumulation of aneuploidies and other structural variations [21,43,44]. Indeed, aneuploidies of Chr6 are known to induce CIN because copy number imbalances of Chr6 resident genes *ACT1* (actin) and *TUB2* (beta-tubulin) disrupt spindle assembly and positioning, increasing nondisjunction rates [14,21,45–47] (S2 Appendix). In contrast, the dark CAN^R^ clones recovered from the same cultures had acquired only the predicted UPD, with none of the additional karyotypic alterations harbored by their matched light counterparts (Fig 5B). Given the high aneuploid burden and ongoing instability present in the light clones, it is improbable that they were the direct predecessors of the dark clones, particularly within the generational limits of our experiments. Rather, these genotypic patterns imply that revertant UPDs can also arise by mechanisms that bypass a stable aneuploid intermediate state. One proposed mechanism, called reciprocal UPD, posits that intermolecular DNA- or protein-based linkages persisting between homologs in mitosis may facilitate their simultaneous nondisjunction, producing daughter cells each carrying a UPD of one homolog [30]. Yet, direct evidence demonstrating that intermolecular linkages are sufficient to drive cooperative missegregation of multiple chromosomes has remained limited.

Another karyotypic pattern captured in the WGS analysis provided insight into the plausibility of such a linkage-driven model of aneuploidy generation: while most dark CAN^R^ clones harbored a typical UPD, ~ 10% harbored a disomic genotype defined by a short heterozygous tract spanning the centromere and bounded by pericentromeric breakpoints (Fig 5C). Dark CAN^R^ clones from the Chr4 strain exhibited a pronounced enrichment for this genotype (8/10 sequenced clones) (Fig 5D), a finding that was particularly surprising given that the *CAN1* markers on Chr4 were separated by more than 750kb and positioned at least 300kb from the centromere in either direction. In principle, such molecules could form via independent, sequential recombination events on each arm, but this mechanism would not explain the pericentromeric breakpoint clustering we observed. Similarly, although centromeric gene conversion—in which breaks in one homolog’s centromere are repaired using sequence from the other [48]—can generate recombinant molecules, the resulting breakpoint patterns are distinct from those observed in dark CAN^R^ clones. Instead, their breakpoint bias more closely resembles the mutational signature generated by the break–fusion–bridge cycles of dicentric chromosomes [49–51]. Previous studies have shown that when dicentric chromosomes are engaged by microtubules emanating from opposite spindle poles in mitosis, they are broken during cytokinesis, typically at positions near one of the centromeres [49,52,53]. Although neither WGS nor the visible phenotypes of these recombinant clones indicated the presence of dicentric molecules undergoing iterative cycles of breakage and repair, the similarity in breakpoint distribution suggested the existence of alternative molecular scenarios in which two centromeres would behave as though physically linked.

We reasoned that sister chromatids, homologs, or even nonhomologous chromosomes connected by persistent intermolecular linkages during mitosis could mimic the missegregation dynamics of a dicentric molecule and result in the formation of the recombinant products recovered in the CAN^R^ clones [49,51,54–60]. One variation of this model—in which such linkages persist between sister chromatids—is illustrated in Fig 5E. If intermolecularly linked sisters segregate to opposite spindle poles during mitosis, the persistent linkages between them will form DNA bridges during anaphase. Left unresolved, this bridged DNA will be severed during cytokinesis [49,54,56,61]. The resulting daughter cells will inherit complementary fragments of the linked pair: one retaining only the centromeric fragment, the other retaining the distal arm fragments together with the intact sister chromatid (Fig 5E). In the next cell cycle, homology-templated repair of these fragments would generate recombinant chromosomes [56]. Repair of the centromere-containing fragment using the homolog as the template would yield a homologous pair that retains heterozygosity near the centromere but is homozygous along the arms—precisely the signature displayed by the recombinant CAN^R^ clones (Fig 5C-D). Oppositely, repair of the arm fragments using either the sister chromatid or the homolog as a template would result in trisomy or tetrasomy, with at least one molecule potentially containing sequences derived from both homologs.

Under this model, and constrained by the positioning of the dual *CAN1* cassettes in our system, clones that inherited the centromeric fragment were more likely to be recovered by CAN^R^ selection than the reciprocal trisomic or tetrasomic products, since these latter products would likely retain at least one *CAN1* cassette and fail to form colonies on canavanine-containing media. Indeed, in the > 500 CAN^R^ clones sequenced in this study, only a few recombinant trisomes displayed the allelic pattern predicted in Fig 5C. Thus, to more rigorously test this model, we engineered a new strain in which the homologs of Chr12 harbored either a single *CAN1* or pigment cassette ~131kb from the centromere and 19kb from the left telomere (Fig 5F). This positioning increased the likelihood of recovering recombinant trisomic products, although most dark CAN^R^ clones were expected to harbor simple copy-neutral LOH tracts that eliminated the *CAN1* cassette and duplicated the pigment operon. Indeed, 77.7% (35/45) of dark CAN^R^ clones recovered harbored short terminal tracts of LOH near the chromosome end (Fig 5G). However, the other 22.2% harbored aneuploidies of Chr12—both UPD and trisomy. Consistent with the predictions of the intermolecular linkage model, every trisomic clone contained at least one recombinant molecule with pericentromeric breakpoints (Fig 5H and S7 Fig). In addition, many displayed complex recombination patterns including LOH across all three molecules or intermediate allele frequencies reflecting mosaicism within the sequenced populations. Both suggest that generation of the recombinant trisome may have involved multiple broken molecules or repair templates, or the production of unstable recombinant products [62,63].

**Fig 6:**
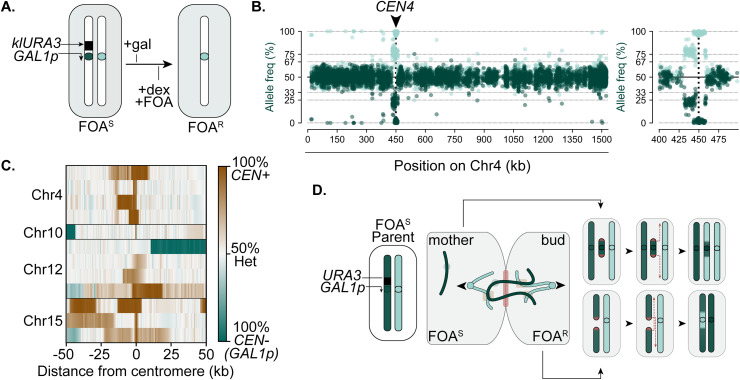
Recombinant chromosome generation coincides with chromosome segregation. **A.** Schematic of the centromere inactivation assay. **B.** A representative Chr4-specific WGS mapping of a FOA^R^ colony recovered from the *URA3-GAL1p-CEN4* strain that displays pericentromeric breakpoints. **C.** A heatmap of allele frequency at each parental heterozygous position illustrating the pericentromere-biased breakpoint positions observed in FOA^R^ clones. **D.** A schematic illustrating the predicted mechanism and outcomes of an intermolecular linkage event that generates an FOA^R^ daughter cell bearing the recombinant genotypes shown in B. and **C.**

The recombinant patterns exhibited by the disomic and trisomic CAN^R^ clones are consistent with a model in which intermolecular linkages between molecules can coordinate their missegregation, leading to breakage and subsequent repair by homologous recombination. Yet, these results did not formally exclude alternative mechanisms such as sequential recombination or centromeric gene conversion. Nor could these CAN^R^ systems directly test a central assumption of the model: that bipolar mitotic attachment is necessary to orient linked molecules in a way that drives their breakage and subsequent recombinant product formation. We used the *URA3-GAL1p-CEN* strains to test this assumption. In yeast, forces generated by the elongating anaphase spindle are required to drive chromosome translocation through the bud neck constriction into the bud cell [64–66]. Conditional centromere inactivation—via *GAL1p* induction—prevents kinetochore assembly and thus coupling of these segregation forces to the specific pair of sister molecules harboring the *URA3-GAL1p-CEN* cassette, resulting in their retention in the mother cell. In this controlled context, only monosomic bud cells arising from divisions in which the *URA3-GAL1p-CEN* sister pair remained unsegregated in the mother cell would be predicted to manifest a FOA^R^ phenotype (Fig 2A). Alternatively, recovery of FOA^R^ cells harboring a recombinant product of the inactivated chromosome would indicate that its formation occurs independently of direct spindle-mediated segregation, but coincident with segregation of the remaining chromosomes. We assessed these possibilities by plating galactose induced populations of *URA3-GAL1p-CEN* strains to solid 5-FOA containing media, allowing FOA^R^ colonies to develop, and isolating individual clones for characterization by WGS (Fig 6A).

Consistent with our previous results (Fig 1D), most sequenced FOA^R^ clones exhibited stable monosomic or revertant mosaic signatures. Yet, 19% displayed a recombinant disomic signature typified by centromere-proximal breakpoints, with Chr4 again overrepresented among this group (4/10 sequenced clones)(Fig 6B and 6C). This recombinant genotype resembled the one displayed by the CAN^R^ clones, with one key difference: whereas CAN^R^ recombinant chromosome pairs retained heterozygosity only near the centromere, FOA^R^ recombinant pairs were homozygous in the pericentromeric region but retained heterozygosity along the length of the chromosome arms (Fig 6B and 6C). Such products could only arise if the arm segments of an inactivated chromatid were segregated into the bud cell—a process that, to our knowledge, cannot occur in the absence of spindle-generated force. Rather, this result implies that bipolar spindle-generated forces were transmitted to the inactivated chromatid by an indirect mechanism, the most parsimonious being via linkage to actively segregating chromosomes (Fig 6D). As illustrated in Fig 6D, a possible outcome of this type of event would be the breakage and partial inheritance of the inactivated chromatid. The daughter cell inheriting the arm fragments would become FOA^R^ if those fragments were repaired using the homolog as a template, generating a pair of chromosomes precisely displaying the recombinant signature observed in the FOA^R^ clones. This result refines a central principle of the intermolecular linkage model: while mitotic chromosome segregation is necessary for recombinant molecule formation, the direct segregation of linked partners is not. Intermolecular linkages can sufficiently transfer the forces necessary to establish a chromosomal architecture that will result in breakage and asymmetric inheritance of the linked molecules.

22% of sequenced FOA^R^ Chr12 clones were typified not by pericentromeric breakpoints, but by a breakpoint within the ribosomal DNA (rDNA) array on the right arm instead (S8 Fig). Due to the homotypic and repetitive architecture of the rDNA array, we were unable to define the precise location of these breakpoints. Like the Chr12 trisomic clones (Fig 5F-G), these clones usually displayed mosaic coverage and allele frequencies, indicating the existence of multiple distinct molecules of Chr12. Such patterns are consistent with a model in which intermolecular linkages within the rDNA lead to chromosome breakage during mitosis [56], and that repair of these fragments may occur through a process yielding diverse molecular outcomes. Notably, in addition to the selected chromosomal aneuploidy, 19% of FOA^R^ clones derived from the other *URA3-GAL1p-CEN* strains also harbored terminal tracts of LOH originating in the rDNA and extending to the right telomere. Elevated incidence of LOH distal to the rDNA has been widely observed in yeast [28,42,67–69] The coincidence of this independent mutation with the recombinant reversion of other chromosomes suggests that the rDNA may be a hotspot for persistent linkage formation (S8 Fig).

**Fig 7:**
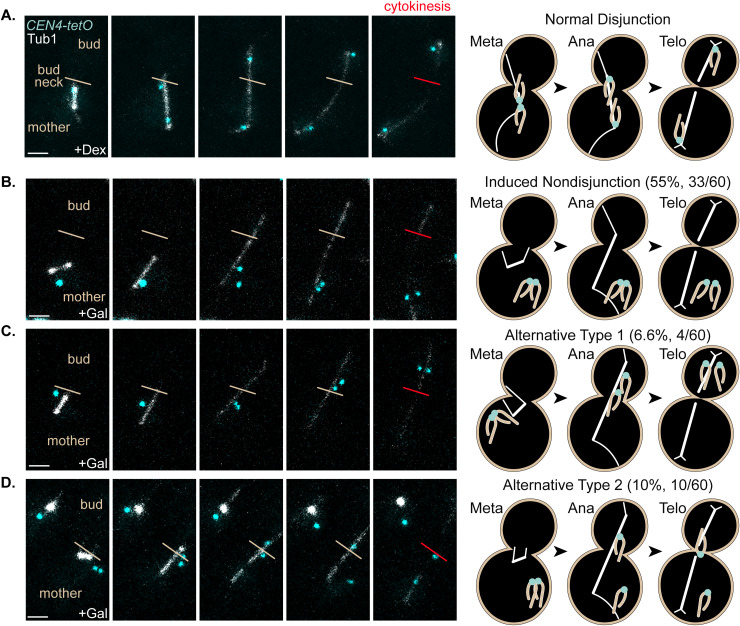
Chromosome missegregation occurs independently of direct spindle attachments. **A.-D.** Representative time-lapse image series (left) and interpretive schematic and quantitation (right) of *URA3-GAL1p-CEN4-tetO* cells expressing TetR-GFP and Tub1-mRuby2: **A.** a cell in dextrose-containing media progressing normally through mitosis, **B.-D.** cells in galactose-containing media displaying (**B.**) the expected nondisjunction of the inactivated chromosome pair encoding, (**C.**) Alternative Type 1 missegregation in which both inactivated chromosomes segregate into the bud cell (**D.**), or Alternative Type 2 missegregation in which the inactivated molecules migrate into the budneck region prior to cytokinesis. The red budneck delineation denotes the completion of mother-bud cell separation by cytokinesis as assessed by brightfield imaging. Scale bars, 2μM.

Although the *URA3-GAL1p-CEN* system generates a mitotic scenario that would rarely occur in wild-type cells—because an unattached chromatid pair typically results in a mitotic checkpoint-mediated cell cycle arrest until attachment is achieved [70,71]—it constituted a valuable tool to further probe the validity of the intermolecular linkage model. Next, we used time-lapse fluorescence microscopy to visualize the segregation dynamics of *CEN4* throughout mitosis in dextrose- and galactose-containing media [72,73]. Into the *URA3-GAL1p-CEN4* strain, a multicopy *tetO* array was introduced adjacent to the *GAL1p-CEN4* cassette, along with fluorescently-tagged alleles of the *tetO*-binding protein (TetR-GFP) and alpha tubulin (mRuby-Tub1). Proliferating populations were imaged over time and the *CEN4* disjunction dynamics in mitosis were assessed in each condition. As expected, 100% of observed *CEN4* disjunction events (59/59) occurred normally in dextrose-containing media: the duplicated *tetO* loci localized between the spindle poles in prometaphase and, at anaphase onset, remained closely associated with the spindle poles as they separated into the mother and bud cells (Fig 7A and S1 Movie). In galactose-containing media, we expected to see two disjunction phenotypes: 1) normal disjunction in cells that had not sufficiently inactivated *CEN4* [74], and 2) inactivation-mediated nondisjunction, in which both *tetO* loci dissociated from the spindle throughout mitosis and remained unsegregated within the mother cell (Fig 7B and S2 Movie). Indeed, we observed these behaviors in 28.3% (17/60) and 55% (33/60) of dividing cells, respectively. However, we also observed alternative segregation events affecting *tetO* pairs that were clearly dissociated from the spindle at the time of anaphase onset (Fig 7C-D and S3 and S4 Movies). In 6.6% of dividing cells, both *tetO* loci segregated into the bud cell (Fig 7C, Alternative Type 1). Unlike normal disjunction—in which the centromeres remain closely juxtaposed to the poles as the spindle elongates—the *tetO* loci in these cells showed no directed poleward movement at anaphase onset. Only late in anaphase, when the spindle reached its maximum length, did the two *tetO* loci abruptly translocate into the bud cell (Fig 7C).

In approximately 10% of cells, one or both *tetO* loci migrated to the bud neck region before moving either into the bud cell or back into the mother cell (Fig 7D, Alternative Type 2). Two distinct behaviors were observed at this stage. In some cases, bud neck localization was transient and culminated in rapid movement of a *tetO* locus into either compartment at the time of cytokinesis, resembling the dynamics of dicentric chromosome severing [49]. In other cases, *tetO* loci remained at the bud neck for extended periods and drifted slowly back into the cell interior only after cytokinesis had occurred (Fig 7D). *TetO* pairs displaying these alternative segregation patterns lacked any detectable colocalized Nuf2, an outer kinetochore protein required for microtubule binding (S9 Fig), again demonstrating that their bud-cell directed movements occurred independently of direct spindle attachment but concurrent with chromosome segregation.

**Fig 8:**
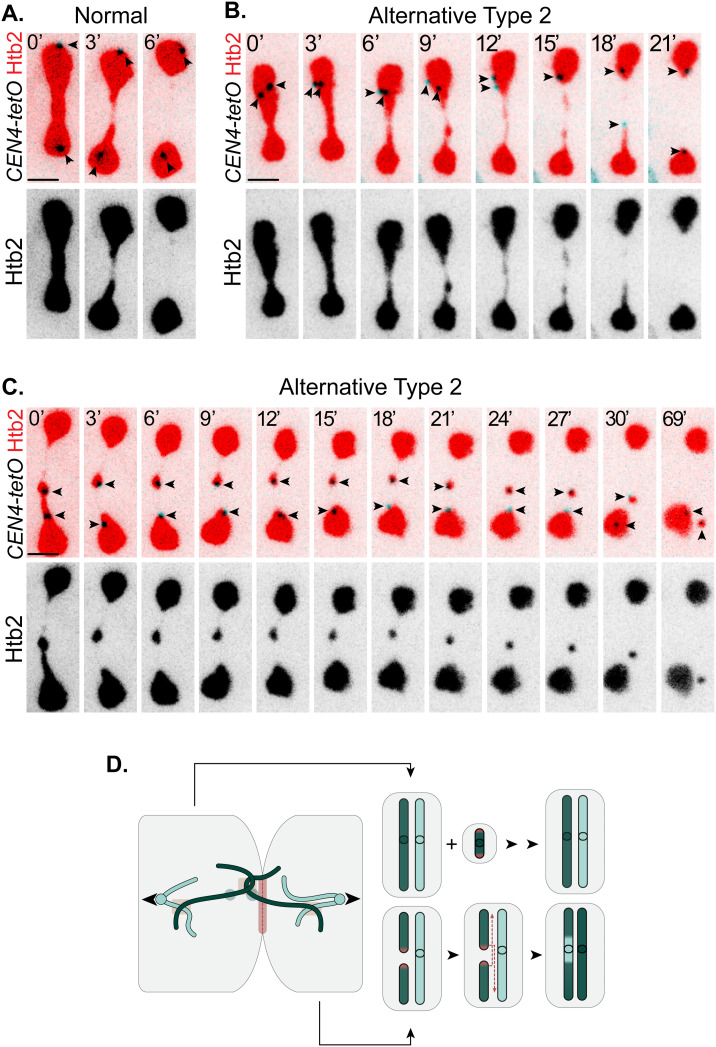
Chromatin bridges and chromosome fragmentation coincide with alternative missegregation events. **A.-C.** Representative time-lapse image series of *URA3-GAL1p-CEN4-tetO* cells expressing TetR-GFP and Htb2-tdTomato that exhibit: **A.** normal segregation of the *CEN4-tetO* locus and resolution of the dividing nuclei, **B.**, alternative *CEN4-tetO* segregation and persistence of a long-lived chromatin bridge that resolves only after the rapid translocation of one *tetO* locus into the bud cell, and **C.**, alternative *CEN4-tetO* segregation and persistence of a chromatin bridge that resolves to form a *tetO*-containing chromosome fragment. Time-stamps (in minutes) are relative to the first frame of the displayed series. Arrowheads denote the two *tetO* loci. **D.** A schematic illustrating the observed chromatin dynamics and predicted molecular outcomes of the alternative missegregation even displayed in **C.** Scale bars, 2μM.

To determine whether intermolecular linkages with actively segregating chromosomes facilitated these alternative *tetO* segregation behaviors, we repeated this experiment using a strain expressing a fluorescently tagged histone (Htb2-TdTomato) and analyzed the chromatin dynamics in Alternative Type 2 cells. Consistent with the intermolecular linkage model, 100% displayed chromatinized DNA bridges spanning the dividing nuclei and encompassing at least one *tetO* locus for variable durations—this was never observed in cells exhibiting normal segregation dynamics (Fig 8A). Transient, rapid movement of *tetO* loci into the bud neck coincided with bridge resolution (Fig 8B and S5 Movie), indicating that these bridges consisted of chromatin linking at least one unattached *tetO*-bearing chromatid to a chromosome that had segregated into the bud cell. As is shown in Fig 8B, only after the abrupt, late-stage translocation of one *tetO* locus through the bud neck does the chromatin bridge which spanned the dividing nuclei for the duration of anaphase finally resolve (Fig 8B, black arrowhead, 18’). Prolonged localization of *tetO* loci at the bud neck reflected a distinct chromatin architecture and mode of bridge resolution, which occurred only at cytokinesis through the apparent severing of chromatin proximal to the bud neck. In 38% of cases, this process generated a chromatin fragment containing at least one *tetO* locus; this fragment was never reincorporated into the primary nuclear compartment of the inheriting daughter cell but instead remained cytoplasmically separated throughout the subsequent cell cycle. In the example shown in Fig 8C, both *tetO* loci are encompassed within the chromatin bridge, although their relative positions with respect to the bud neck differ. In this instance, the orientation of the *tetO* loci and the Htb2 signal suggests that the unattached *tetO-*bearing chromatids may be linked both to one another and to chromosomes that have segregated into each cell. Following cytokinesis, the *tetO* locus positioned closest to the bud neck resolves into a new, chromatinized compartment in the mother cell, leaving the bud cell devoid of this *CEN4*-adjacent DNA fragment (Fig 8C and S6 Movie). Together, these observations demonstrate that intermolecular linkages promote centromere-proximal breakage and loss of centromere-bearing DNA, leading to daughter cells with incomplete or damaged chromosomes. These findings directly support our proposed mechanism of recombinant CAN^R^ and FOA^R^ clone formation (Fig 8D).

## Discussion

The inherent reversibility of aneuploid karyotypes has long been recognized, yet the impacts on population-level dynamics have remained difficult to discern. Here we empirically quantified the occurrence, associated fitness effects, and reversibility of monosomic karyotypes. We developed new mutational models with which to integrate these data, yielding a unified framework of the determinants of aneuploid karyotype diversity and stability in populations. Because we evaluated monosomies of multiple different chromosomes, this framework encompasses the physiologically relevant range of rates and fitness coefficients associated with aneuploidy in *S. cerevisiae*, enabling us to articulate general principles of aneuploidy dynamics and to resolve additional mutational mechanisms that impact the basal stability and inheritance of chromosomes in unperturbed conditions.

A primary goal of this study was to define how reversion constrains aneuploid karyotype stability. We find that the dynamics of canonical 2-step reversion are generally explained by an unequal likelihood mutational model: a cell loses a chromosome and then, via a subsequent random nondisjunction event, reverts to a disomic state. Reversion rates varied across chromosomes but were uniformly low (<10^-4^ per cell per generation), indicating that 2-step reversion contributes minimally to the stability of aneuploid subpopulations under conditions where populations expand from euploid precursors in permissive, nearly static environments (S2 Appendix). In such scenarios, the relative fitness of aneuploid cells is the primary determinant of karyotype frequencies, suggesting that selection, rather than reversion, dominates the maintenance and diversity of aneuploid lineages. The fitness costs and mutation rates derived in the growth conditions used in this study are likely to differ across stressful or fluctuating environments. Consequently, and in line with a growing body of work, the extent to which natural selection shapes karyotypic diversity within populations is strongly dependent on the environment [75]. However, reversion may become a more influential determinant of karyotype stability in populations that are already highly karyotypically diverse [76–80], or in those undergoing major shifts in effective size or structure—such as bottlenecks, founder events, or selective sweeps. Indeed, reversion had a markedly greater impact on the karyotypic diversification of populations derived from aneuploid founders than from euploids. Moving forward, the influence of specific environmental or population characteristics, and how they alter the primary determinants of aneuploid karyotype stability, can be systematically interrogated using the experimental systems and computational models presented in this work.

A second objective of this study was to investigate aneuploidy dynamics across a broad chromosomal context. We find substantial variation in nondisjunction incidence across the different yeast chromosomes. Discerning the underlying basis for this variance warrants further study, as doing so is likely to clarify central principles of genome and chromosomal stability and refine current paradigms of aneuploidy-driven disease risk. Perhaps variations in centromeric sequences, which are highly variant and fast-evolving [48,81–86], may underlie chromosome-specific differences in kinetochore assembly and disjunction fidelity. Alternatively, the spatial organization of chromosomes within the nucleus may impact the likelihood of nondisjunction [87–89]. Our work presents an additional possibility: for certain chromosomes—particularly those exhibiting the highest rates of loss—nondisjunction may more frequently occur through a coupled mechanism defined by intermolecular linkage-mediated missegregation, chromosome breakage, and homologous repair.

DNA bridges arise in mitosis from intermolecular linkages with diverse molecular origins, including incomplete DNA replication [60,90,91], defective cohesion or condensin degradation [92,93], residual catenanes [54,90,92,94], and unresolved recombination intermediates [61,95].The molecular features of these bridges elicit distinct cellular responses that differentially affect chromosome stability. For example, wild type mammalian cells form catenane-driven ultrafine centromeric DNA bridges each cell cycle, but these are efficiently recognized and resolved by the BLM/PICH/FANC complex within the normal timeframe of mitosis [54,96]. Other bridge types are not resolved within mitosis but instead trigger a delay in cytokinesis via the NoCut checkpoint [97,98], allowing lagging chromatin to complete segregation before cytokinetic abscission. Bridges that evade recognition, repair, or resolution result in chromosome fragmentation, micronucleus formation, or cytokinetic failure—outcomes collectively associated with genomic instability [96]. While these catastrophic outcomes are hallmarks of genetically unstable cell types including cancer cells and DNA stability mutants [99], their observed incidence in wild type cells is rare [61,90,98]. Our data indicate that damage-inducing linkages form stochastically in wild type cells and that chromosome-specific propensities to form such linkages—perhaps due to the presence of hyper-recombinant loci, the enrichment of cohesin and condensin complexes, etc.—are an important, yet understudied contributor to the observed diversity in nondisjunction rate in wild type populations.

Although we have endeavored to define a unified framework of aneuploid karyotype dynamics that can be explored *in silico*, the genetic systems used in this study allowed empirical detection of but a subset of outcomes predicted by our models of linkage-mediated missegregation, suggesting that we have substantially underestimated the true incidence of this mutational mechanism in populations. For example, recombinant products formed when linked molecules are severed in repetitive regions—such as the rDNA [56], subtelomeric arrays, telomeric repeats, or dispersed repeats—may not resolve with detectable breakpoints or heterozygous tracts displayed by the recombinant clones characterized in this study. Instead, these events would resolve as typical UPDs, making them indiscernible from the revertant karyotype formed through the canonical 2-step reversion pathway. This possibility is particularly salient when interpreting the reversion dynamics of Chr12, which displayed an exceptionally high reversion rate and penetrant revertant WGS signature consistent with a typical UPD. Genetic and chemical perturbations affecting rDNA transcriptional regulation, condensation, and decatenation have been shown to promote the formation of intermolecular rDNA linkages and chromosome severing in cytokinesis [56,100–102]. Our work prompts the hypothesis that even in unperturbed wild type cells, persistent linkages existing within the ~ 1Mb rDNA array facilitate high-frequency chromosome nondisjunction events capable of resolving into a simple UPD karyotype through a complex mechanism of templated repair [42,56]. Our future studies will seek to define the positional and architectural features of such molecular linkages within the rDNA and genome-wide, to determine why some chromosomes appear subject to this mode of genomic instability more than others, and to discern the molecular mechanisms by which repair of severed chromosomes occurs subsequent to nondisjunction.

## Methods

### Strains and media

Strains and plasmids used in this study are listed in Table A in S1 Appendix. Strain construction was performed using standard transformation, crossing, and sporulation procedures. Directed insertions of selectable cassettes and reporter constructs were validated using PCR and WGS. Depending on the experiment, yeast cells were grown in one of the following media: rich nonselective media (10g/L yeast extract, 20 g/L Peptone, 20 g/L bacteriological agar, and 20g/L glucose (2.0%)), synthetic-defined complete media (20g/L glucose or 20g/L galactose, 5g/L ammonium sulfate, 1.7g/L yeast nitrogen base without amino acids, 1.4g/L complete drop-out mix, 20g/L bacteriological agar), canavanine-containing media (20g/L glucose, 5g/L ammonium sulfate, 1.7g/L yeast nitrogen base without amino acids, 1.4g/L arginine dropout mix, 20g/L bacteriological agar, 0.06g/L canavanine sulfate), uracil drop-out media (20g/L glucose or 20g/L raffinose, 5g/L ammonium sulfate, 1.7g/L yeast nitrogen base without amino acids, 1.4g/L uracil dropout mix), 5-FOA containing media (20g/L glucose, 5g/L ammonium sulfate, 1.7g/L yeast nitrogen base without amino acids, 1.4g/L complete drop-out mix, 20g/L bacteriological agar, g/L 5-Fluoroorotic Acid).

### Canavanine fluctuation tests

*Liquid media pregrowth:* For each *CAN1*-marked strain, replicate ~500-cell populations were seeded into microwells containing YPD and expanded for 48hrs (median population size = 7.3x10^6^, median number of generations = 13.8). *Solid media pregrowth:* Each *CAN1*-marked strain was streaked onto YPD plates to single cell density and independent colonies were allowed to develop for 48hrs before isolating each and resuspending in 200uL of sterile water. *Plating:* A fraction (20–40%; average 3-7x10^6^ cells) of each replicate was plated on canavanine-containing media, and a diluted fraction plated on YPD. Plates were incubated at 30°C for 96 hrs; both dark CAN^R^ and YPD-plated colonies were counted after 48hrs, the light CAN^R^ colonies at 96hrs. *Data availability:* Raw colony counts, frequency calculations, and inferred population sizes are presented in Table G in S1 Appendix. For strains LRH587, LRH585, Chr684, and LRH588, we were unable to successfully integrate the complete VIO cassette, even after multiple attempts. Therefore, revertant phenotypes were deduced on the basis of colony size at 48hrs. WGS demonstrated that revertant genotype identification by colony size was accurate.

### Monosome replating tests

*Plating:* Small (48–72hr) light CAN^R^ colonies isolated from independent canavanine plates were resuspended and diluted to single-cell density on synthetic defined complete media plates and incubated at 30°C for 48hrs. After 48hrs, dark revertant colonies were quantified; after 96hrs, light monosomic colonies were quantified. *Data availability:* Raw colony counts, frequency calculations, and inferred population sizes are presented in Table E in S1 Appendix. *Visualization:* The frequency of revertant clones in each replicate monosomic population assessed are presented in Fig 1D in a plot created in Graphpad Prism 10.

### Rate estimation using mlemur

*Rate estimation:* We used mlemur v0.9.6.5 [39] to estimate the mutation rates for all the experimental fluctuation test data. The batch mode in the graphical user interface was used to input selective and non-selective colony counts, culture volumes, dilution factors and relative mutant fitness. *Data availability:* The colony count input and rate outputs for the monosome replating assay and canavanine fluctuation tests are presented in Tables F and H in S1 Appendix, respectively. *Visualization:* The rates of reversion estimated from the canavanine fluctuation tests and monosome replating tests are presented in Fig 3A and 3B with plots created in Graphpad Prism. The comparative plot in Fig 3C was made with a custom plotting script (see Data Availability).

### Relative fitness quantification

*Preselected monosome assay:* For monosomes of Chr1, Chr2, Chr3, Chr8, Chr9, Chr14, and Chr16, 3 independent 72hr grown light CAN^R^ clones were isolated and inoculated in 1mL of liquid rich YPD media. A diploid control strain constructed in the same hybrid genetic background was also inoculated in triplicate and all cultures were grown overnight at room temperature to saturation. Saturated cultures were then diluted 20-fold into fresh YPD and dispensed in duplicate into microwell dishes. *Induced monosome assay:* The pGAL1-CEN strains were grown overnight in 1mL cultures of liquid uracil drop-out media containing 2% raffinose. These cultures were then diluted 3-fold into synthetic defined complete media containing 2% galactose and grown for 2.5hrs to induce centromere inactivation. At least 5 replicate 100-fold dilutions were seeded into 384-well microplates (Corning cat. 3680; Corning, New York) containing synthetic defined complete media containing 2% glucose and 1mg/L 5-FOA, in a total volume of 80 μL per well. *Optical density measurements:* Growth kinetics were tracked over 36 hrs by measuring the optical density (OD600) of each microwell every 20 min in a Cytation3 (BioTek) plate reader heated to 30°C without shaking. *Growth curve analysis:* For each replicate growth curve, the maximum growth rate was estimated using custom Python v3.10 [103] scripts. Linear regressions of OD600 values against time were fitted for overlapping sliding windows of 20 timepoints (6.67 hrs) using scipy v1.14 [104], and the maximum slope value was used. *Determination of fitness coefficients:* The average maximum slope values calculated from each replicate monosomic population of a given strain or isolate was divided by the average maximum slope value calculated from the replicate growth curves of the appropriate diploid control strain. *Data availability:* Raw slope values and fitness coefficients are presented in D in S1 Appendix. *Visualization:* The plot of inferred relative fitness coefficients and simple linear regression analysis presented in Fig 2B was generated using Graphpad Prism 10.

### Whole Genome Sequencing (WGS) library preparation

The information for all clones sequenced in this study is deposited in Table I in S1 Appendix. WGS of selected clones was performed as described previously [12,15]. Briefly, candidate colonies were isolated using sterile toothpicks, repatched to rich media plates, and expanded for one day. The entire patch of cells was then used to isolate genomic DNA using the Yeastar Genomic Kit from Zymo Research (cat. D2002; Irvine, CA). DNA quantity and quality was assessed using a Qubit fluorometer (Thermo Fisher Scientific). Pooled, barcoded libraries of 96–384 individual genomes were generated using a Seqwell plexWell-384 kit. The final barcoded libraries were sequenced at Novogene Corp. using an Illumina Novaseq sequencer.

### WGS data analysis

*Read processing and trimming:* Reads were trimmed using fastp v0.23.4 [105] with default options. *Read mapping:* Reads were mapped to the *S. cerevisiae* reference genome vR64 (http://sgd-archive.yeastgenome.org/sequence/S288C_reference/genome_releases/) using bwa-mem2 v2.3 [106] with default options. Secondary alignments were filtered out using samtools v1.21 [107]. Picard v3.1.1 (http://broadinstitute.github.io/picard) was used to mark and remove duplicates (MarkDuplicates) and add read groups to the BAM files (AddOrReplaceReadGroups). *Variant detection:* Variants were called using bcftools v1.21 [108], first generating pileups (mpileup) and subsequently calling the variants (call) with options -m -v. Biallelic single nucleotide variants were kept for the genome analyses. *Data availability:* Per chromosome assessment of the total clones sequenced and the percentage of which harbor defined karyotypic variations is presented in Table B in S1 Appendix. Raw sequencing data are deposited at NCBI under Bioproject PRJNA1414607.*Visualization:* Figs 1C, 5A, 5B, 5G, 5H, 6B, 6C, and 6E were made with custom scripts deposited on this study’s Github page.

### Monosome copy number analysis

*Genomic analysis:* The WGS data derived from each of the light CAN^R^ clones which harbored only a monosome of the marked chromosome were remapped to a repeat masked reference assembly. The use of a masked reference eliminated the possibility that mismapping to repetitive regions of the genome (*i.e.*, the rDNA array, subtelomeric repeats, mobile elements) would distort coverage analyses and allowed us to more precisely calculate the normalized copy number of the marked chromosome relative to the rest of the genome. The average depth of coverage of the respective marked chromosome was divided by the mean coverage of the other 15 chromosomes and normalized to relate a copy number value. *Data Availability:* Per clone normalized copy number values are presented in Table C in S1 Appendix. *Visualization:* The deduced copy numbers of each monosome analyzed are presented in Fig 1D in a plot created in Graphpad Prism 10.

### Forward simulation of fluctuation assays

We developed fflucsim, a forward simulation tool to generate in silico populations expanding from a single diploid cell ancestor. fflucsim is a Python library that includes objects and functions to generate cells and expand them into large populations under adjustable parameters dictating the population dynamics that generate monosomic karyotypes and the mutational events immediately following that revert to disomic karyotypes. Briefly, founder cells are initiated with user-specified values for aneuploidy rates and relative fitness of aneuploid karyotypes. Cells divide synchronously [109] using relative fitness and mutation rates as probabilities of karyotype change and generation of a daughter cell, respectively, until a user-specified target population size is reached. Cells implement two distinct homologs, one carrying a counter-selectable marker, which enables the selection of surviving cells at the end of the population expansion (mimicking the *CAN1* cassettes in our experimental fluctuation assays). Importantly, fflucsim keeps full records of the parameters, mutation events and full genealogy in cell and population objects, making it a uniquely powerful framework to track the dynamics of complex mutations with alternative paths to back-mutation. The source code and the Snakemake v9.15 [110] scripts used for all simulations presented in this study are available at GitHub (see Data accessibility section).

### Microscopy

*Growth conditions:* Cells were pregrown to log phase in synthetic-defined complete media containing 2% raffinose, before dilution into complete media containing either 2% glucose or 2% galactose. After 90 min of growth, cells were prepared for imaging. *Slide preparation:* Cells were concentrated, spotted on a 1.5% agarose pad containing the same respective media, covered with a coverslip and sealed with paraffin. *Imaging procedures:* Images were collected on a Nikon Ti-E microscope equipped with a 1.45NA100 × CFIPlan Apo objective, piezo electric stage (Physik Instrumente; Auburn, MA), spinning disk confocal scanner unit (CSU10; Yokogawa), 488-nm and 561-nm lasers (Agilent Technologies; Santa Clara, CA), and an EMCCDcamera (iXonUltra 897;AndorTechnology, Belfast, UK) using NIS Elements software (Nikon). Z-series spanning 7–9 μm with 0.5 μm steps were acquired every 3min for 2 hours. *Data Availability*: Raw image files will be provided upon request. *Visualization*: Image files were imported as hyperstacks and processed using Fiji [111]. Maximum projections of z-series for each channel were generated. Intensity and color adjustments, cropping, and merging was performed with tools in either Fiji or Adobe Photoshop.

## Supporting information

S1 Fig Karyotypic alterations associated with the light and dark CAN^R^ phenotype on a per- chromosome basis. **A., B.** Quantification of the percentage of light CAN^R^ (**A**) and dark CAN^R^ (**B**) clones that harbored the denoted karyotypic states affecting the predicted chromosome (Selected) as well as any additional unselected events (Unselected).
(TIF)

S2 Fig Divergent late-stage growth dynamics suggest the sweep of revertant karyotypes. OD600 growth curve data and end point microwell images of representative strains subjected to the induced monosome generation fitness assays. Black arrowheads point to late-stage, rapid, increases in growth rate indicative of emergent revertant subpopulations. Grey boxes in each plot denote the growth kinetics data that were excluded from our estimate of growth rate to eliminate revertant emergence as a confounding factor.
(TIF)

S3 FigMutation rates are not correlated with chromosomal features.Log-transformed per-chromosome estimates of the rates of monosomy and reversion plotted by chromosome size **(A.)** or the length of each chromosome arm **(B.)**. Vertical error bars denote the 95% confidence intervals associated with each per-chromosome estimate of μ^A^ or μ^R^. Brown and green areas depict the 95% confidence intervals produced from the simple linear regression of μ^A^ (brown) μ^R^ (green) or vs. chromosome size.(TIF)

S4 Fig Parallel mutational routes can generate revertant UPDs. A schematic illustrating genotypes, phenotypes, and rates associated with nondisjunction events that result in the formation and subsequent reversion of monosomic and trisomic daughter cell pairs. Symbols are the same as in Fig 1. Only the derivatives outlined with a solid line would be recoverable in the canavanine fluctuation tests.
(TIF)

S5 FigValidation of fflucsim using back-calculation of mutation rates from simulated mutant counts.Mutation rates (dots) and 95% confidence intervals (vertical lines) were estimated from simulated mutant counts using rSalvador [41]. We performed 245 simulations across all combinations of seven μ^A^ values (10^-7^–10^-4^), seven μ^R^ values (10^-7^–10^-1^) and five ω^A^ values (10^-2^–10^0^). For these validation simulations, we ignored reversion that occurs via the trisomic counterparts of monosomy events, setting μ^T^=0 and ω^T^=1. We performed 50 replicates for each parameter combination. Two models were fitted on simulated mutant counts, the standard Luria-Delbrück model (Lea-Coulson, open dots) and the model accounting for variable mutant fitness (Mandelbrot-Koch, closed dots). The rate values expected from the input parameters are shown as thin background lines. Input parameters are summarized as heatmaps below each plot. Monosome rates (μ^A^) were estimated from combined counts of monosomic and revertant mutants (**A**), and compound rates (μ^C^ = μ^A^ × μ^R^) were estimated from revertant mutant counts alone (**B**).(TIF)

S6 FigEmpirical revertant frequencies deviate substantially from the expected value for a subset of chromosomes.**A.** Cumulative distributions of revertant cell (R) frequency for the monosome replating tests (dark green) and simulated populations generated by fflucsim (light green). The fflucsim distributions correspond to the 50 replicates of the single simulated assay parameterized with the pair of μ^R^ and ω^A^ values closest to the empirical estimates for the corresponding chromosome. **B.** Heatmaps showing the same data as Fig 3D with revertant frequency mapped on a logarithmic scale. Insets highlight the set of input parameters closest to the empirical estimates.(TIF)

S7 Fig Pericentromeric recombination patterns suggest an intermolecular linkage-mediated mechanism of chromosome nondisjunction. Chr12-specific whole-chromosome or centromere-proximal WGS mappings of the recovered trisomic CAN^R^ colonies. Black arrowheads denote the position of *CEN12.*
(TIF)

S8 Fig Breaks within the ribosomal DNA coincide with FOA^R^ karyotype formation. **A.** Representative Chr12-specific WGS mappings from recombinant FOA^R^ colonies derived from the *URA3-GAL1p-CEN12* strain displaying a breakpoint within the rDNA locus. **B.** A schematic illustrating the predicted mechanism and outcomes of a nondisjunction event resulting from intermolecular linkages arising in the rDNA. **C.** A representative WGS mapping and corresponding Chr12-specific WGS mapping of a FOA^R^ clone recovered from the *URA3-GAL1p-CEN10* strain. As shown in the top mapping, this clone is monosomic for Chr10 and also harbors a mosaic structural variation involving the right arm of Chr12 beginning at a breakpoint in the rDNA. The bottom mapping more clearly illustrates the rDNA breakpoint and mosaic allele frequency on Chr12.
(TIF)

S9 Fig Alternative missegregation occurs in the absence of intact kinetochores. **A. and C.** Representative images of a normal disjunction event (**A.**) and an alternative Type 2 missegregation event (**C.**) occurring in *URA3-GAL1p-CEN4-tetO* cells expressing TetR-GFP and Nuf2-tdTomato, a kinetochore protein required for spindle microtubule attachment. **B. and D.** Corresponding line scan analysis illustrating the degree of TetR and Nuf2 signal overlap in **A.** and **C.**; Line scan analysis was performed by determining the pixel intensity across the distance spanning all four fluorescent foci for both detection channels using Fiji.
(TIF)

S1 Movie Representative time-lapse image series of a *URA3-GAL1p-CEN4-tetO* cell expressing TetR-GFP and Tub1-mRuby2 displaying typical chromosome segregation dynamics in glucose-containing media. (AVI)

S2 Movie Representative time-lapse image series of a *URA3-GAL1p-CEN4-tetO* cell expressing TetR-GFP and Tub1-mRuby2 displaying the expected centromere inactivation nondisjunction dynamics in galactose-containing media. (AVI)

S3 Movie Representative time-lapse image series of a *URA3-GAL1p-CEN4-tetO* cell expressing TetR-GFP and Tub1-mRuby2 displaying Type 1 nondisjunction dynamics in galactose-containing media. (AVI)

S4 Movie Representative time-lapse image series of a *URA3-GAL1p-CEN4-tetO* cell expressing TetR-GFP and Tub1-mRuby2 displaying Type 2 nondisjunction dynamics in galactose-containing media. (AVI)

S5 Movie Representative time-lapse image series of a *URA3-GAL1p-CEN4-tetO* cell expressing TetR-GFP and Htb2-tdTomato displaying Type 2 nondisjunction dynamics of the *CEN4-tetO* loci and persistence of a long-lived chromatin bridge that resolves only after the rapid translocation of one *tetO* locus into the bud cell. (AVI)

S6 Movie Representative time-lapse image series of a *URA3-GAL1p-CEN4-tetO* cell expressing TetR-GFP and Htb2-tdTomato displaying Type 2 nondisjunction dynamics of the *CEN4-tetO* loci and alternative *CEN4-tetO* segregation and persistence of a chromatin bridge that resolves to form a *tetO*-containing chromosome fragment. (AVI)

S1 Appendix Table A. Yeast strains and plasmids used in this study. Table B. Karyotypic variant analysis of sequenced clones. Table C. Per-chromosome monosome depth of coverage analysis. Table D. Data used to calculate relative fitness coefficients. Table E. Monosome replating test count data. Table F. Monosome replating test rate analysis. Table G. Canavanine fluctuation test colony count data. Table H. Canavanine fluctuation test rate analysis. Table I. WGS sample information.
(XLSX)

S2 Appendix Extended Discussion. Additional discussion points regarding the biological contexts for modeling aneuploidy dynamics.
(DOCX)

## References

[1] Bull MJ. Down Syndrome. N Engl J Med. 2020;382(24):2344–52. 32521135 10.1056/NEJMra1706537

[2] Weaver BA, Cleveland DW. The aneuploidy paradox in cell growth and tumorigenesis. Cancer Cell. 2008;14(6):431–3. doi: 10.1016/j.ccr.2008.11.011 19061834 PMC3132552

[3] Turajlic S, Sottoriva A, Graham T, Swanton C. Resolving genetic heterogeneity in cancer. Nature Reviews Genetics. 2019. 10.1038/s41576-019-0114-6 30918367

[4] Selmecki AM, Dulmage K, Cowen LE, Anderson JB, Berman J. Acquisition of aneuploidy provides increased fitness during the evolution of antifungal drug resistance. PLoS Genet. 2009;5(10):e1000705. doi: 10.1371/journal.pgen.1000705 19876375 PMC2760147

[5] Kohanovski I, Pontz M, Vande Zande P, Selmecki A, Dahan O, Pilpel Y, et al. Aneuploidy can be an evolutionary diversion on the path to adaptation. Mol Biol Evol. 2024;41(3):msae052. doi: 10.1093/molbev/msae052 38427813 PMC10951435

[6] Yona AH, Manor YS, Herbst RH, Romano GH, Mitchell A, Kupiec M, et al. Chromosomal duplication is a transient evolutionary solution to stress. Proc Natl Acad Sci U S A. 2012;109(51):21010–5. doi: 10.1073/pnas.1211150109 23197825 PMC3529009

[7] Tan Z, Hays M, Cromie GA, Jeffery EW, Scott AC, Ahyong V, et al. Aneuploidy underlies a multicellular phenotypic switch. Proc Natl Acad Sci U S A. 2013;110(30):12367–72. doi: 10.1073/pnas.1301047110 23812752 PMC3725063

[8] Cromie GA, Tan Z, Hays M, Jeffery EW, Dudley AM. Dissecting gene expression changes accompanying a ploidy-based phenotypic switch. G3 (Bethesda). 2017;7(1):233–46. doi: 10.1534/g3.116.036160 27836908 PMC5217112

[9] Hose J, Zheng Q, Sharp NP, Gasch AP. On the rate of aneuploidy reversion in a wild yeast model. Genetics. 2025;229(2):iyae196. doi: 10.1093/genetics/iyae196 39584496 PMC13031112

[10] Gilchrist C, Stelkens R. Aneuploidy in yeast: Segregation error or adaptation mechanism?. Yeast. 2019;36(9):525–39. 31199875 10.1002/yea.3427 PMC6772139

[11] Gerstein AC, Berman J. Shift and adapt: The costs and benefits of karyotype variations. Current Opinion in Microbiology. 2015;26(August):130–6. 26321163 10.1016/j.mib.2015.06.010 PMC4577464

[12] Heasley LR, Argueso JL. Bursts of genomic instability potentiate phenotypic and genomic diversification in. Frontiers in Genetics. 2022;13(June):912851. 35783258 10.3389/fgene.2022.912851 PMC9247159

[13] Reid RJD, Sunjevaric I, Voth WP, Ciccone S, Du W, Olsen AE, et al. Chromosome-scale genetic mapping using a set of 16 conditionally stable Saccharomyces cerevisiae chromosomes. Genetics. 2008;180(4):1799–808. doi: 10.1534/genetics.108.087999 18832360 PMC2600922

[14] Sheltzer JM, Amon A. The aneuploidy paradox: Costs and benefits of an incorrect karyotype. Trends Genet. 2011;27(11):446–53. doi: 10.1016/j.tig.2011.07.003 21872963 PMC3197822

[15] Heasley LR, Watson RA, Argueso JL. Punctuated aneuploidization of the budding yeast genome. Genetics. 2020;216(1):43–50. doi: 10.1534/genetics.120.303536 32753390 PMC7463280

[16] Sharp NP, Sandell L, James CG, Otto SP. The genome-wide rate and spectrum of spontaneous mutations differ between haploid and diploid yeast. Proc Natl Acad Sci U S A. 2018;115(22):E5046–55. doi: 10.1073/pnas.1801040115 29760081 PMC5984525

[17] Kumaran R, Yang S-Y, Leu J-Y. Characterization of chromosome stability in diploid, polyploid and hybrid yeast cells. PLoS One. 2013. 10.1371/journal.pone.0068094 PMC3707968 23874507

[18] Hose J, Escalante LE, Clowers KJ, et al. The genetic basis of aneuploidy tolerance in wild yeast. eLife. 2020;9. doi: 10.7554/eLife.52063 PMC6970514 31909711

[19] Dutcher HA, Hose J, Howe H, Rojas J, Gasch AP. The response to single-gene duplication implicates translation as a key vulnerability in aneuploid yeast. PLoS Genet. 2024;20(10):e1011454. doi: 10.1371/journal.pgen.1011454 39453980 PMC11540229

[20] Zhu YO, Sherlock G, Petrov DA. Whole genome analysis of 132 clinical Saccharomyces cerevisiae strains reveals extensive ploidy variation. G3 (Bethesda, Md). 2016. 10.1534/g3.116.029397 PMC4978896 27317778

[21] Zhu J, Pavelka N, Bradford WD, Rancati G, Li R. Karyotypic determinants of chromosome instability in aneuploid budding yeast. PLoS Genet. 2012;8(5):e1002719. doi: 10.1371/journal.pgen.1002719 22615582 PMC3355078

[22] Rojas J, Hose J, Dutcher HA, Place M, Wolters JF, Hittinger CT, et al. Comparative modeling reveals the molecular determinants of aneuploidy fitness cost in a wild yeast model. Cell Genom. 2024;4(10):100656. doi: 10.1016/j.xgen.2024.100656 39317188 PMC11602619

[23] Torres EM, Sokolsky T, Tucker CM, Chan LY, Boselli M, Dunham MJ, et al. Effects of aneuploidy on cellular physiology and cell division in haploid yeast. Science. 2007;317(5840):916–24. doi: 10.1126/science.1142210 17702937

[24] Pompei S, Cosentino Lagomarsino M. A fitness trade-off explains the early fate of yeast aneuploids with chromosome gains. Proc Natl Acad Sci U S A. 2023;120(15):e2211687120. doi: 10.1073/pnas.2211687120 37018197 PMC10104565

[25] Peter P, Jackson J, De Chiara M, Friedrich A. Genome evolution across 1,011 Saccharomyces cerevisiae isolates. Nature. 2018;556(7701):339–44. 29643504 10.1038/s41586-018-0030-5 PMC6784862

[26] Sampaio NMV, Ajith VP, Watson RA, Heasley LR, Chakraborty P, Rodrigues-Prause A, et al. Characterization of systemic genomic instability in budding yeast. Proc Natl Acad Sci U S A. 2020;117(45):28221–31. doi: 10.1073/pnas.2010303117 33106418 PMC7668020

[27] Engel E. Uniparental disomy revisited: The first twelve years. Am J Med Genet. 1993;46(6):670–4. doi: 10.1002/ajmg.1320460613 8362910

[28] Pankajam AV, Dash S, Saifudeen A, Dutta A, Nishant KT. Loss of heterozygosity and base mutation rates vary among saccharomyces cerevisiae hybrid strains. G3 (Bethesda). 2020;10(9):3309–19. doi: 10.1534/g3.120.401551 32727920 PMC7466981

[29] Dutta A, Dutreux F, Schacherer J. Loss of heterozygosity spectrum depends on ploidy level in natural yeast populations. Mol Biol Evol. 2022;39(11):msac214. doi: 10.1093/molbev/msac214 36205042 PMC9641995

[30] Andersen SL, Petes TD. Reciprocal uniparental disomy in yeast. Proceedings of the National Academy of Sciences of the United States of America. 2012;109(25):9947–52. 22665764 10.1073/pnas.1207736109 PMC3382553

[31] Chuang J, Boeke JD, Mitchell LA. Coupling yeast golden gate and VEGAS for efficient assembly of the violacein pathway in Saccharomyces cerevisiae. Methods Mol Biol. 2018;1671:211–25. 29170962 10.1007/978-1-4939-7295-1_14

[32] Hill A, Bloom K. Genetic manipulation of centromere function. Molecular and Cellular Biology. 1987;7(7):2397–405. 3302676 10.1128/mcb.7.7.2397-2405.1987 PMC365371

[33] Boeke JD, LaCroute F, Fink GR. A positive selection for mutants lacking orotidine-5’-phosphate decarboxylase activity in yeast: 5-fluoro-orotic acid resistance. Mol Gen Genet. 1984;197(2):345–6. doi: 10.1007/BF00330984 6394957

[34] Tanaka K, Kitamura E, Kitamura Y, Tanaka TU. Molecular mechanisms of microtubule-dependent kinetochore transport toward spindle poles. J Cell Biol. 2007;178(2):269–81. doi: 10.1083/jcb.200702141 17620411 PMC2064446

[35] Oromendia AB, Dodgson SE, Amon AA. Aneuploidy causes proteotoxic stress in yeast. Genes & Development. 2012;26(24):2696–708. 23222101 10.1101/gad.207407.112 PMC3533075

[36] Pavelka N, Rancati G, Zhu J, Bradford WD, Saraf A, Florens L, et al. Aneuploidy confers quantitative proteome changes and phenotypic variation in budding yeast. Nature. 2010;468(7321):321–5. doi: 10.1038/nature09529 20962780 PMC2978756

[37] Luria SE, Delbrück M. Mutations of bacteria from virus sensitivity to virus resistance. Genetics. 1943;28(6):491–511. doi: 10.1093/genetics/28.6.491 17247100 PMC1209226

[38] Lang GI. Measuring mutation rates using the Luria-Delbrück fluctuation assay. Methods Mol Biol. 2018;1672:21–31. 29043614 10.1007/978-1-4939-7306-4_3

[39] Łazowski K. Efficient, robust, and versatile fluctuation data analysis using MLE MUtation Rate calculator (mlemur). Mutat Res. 2023;826:111816. doi: 10.1016/j.mrfmmm.2023.111816 37104996

[40] Worrall JT, Tamura N, Mazzagatti A, Shaikh N, van Lingen T, Bakker B, et al. Non-random Mis-segregation of human chromosomes. Cell Rep. 2018;23(11):3366–80. doi: 10.1016/j.celrep.2018.05.047 29898405 PMC6019738

[41] Zheng Q. rSalvador: An R package for the fluctuation experiment. G3 (Bethesda). 2017;7(12):3849–56. doi: 10.1534/g3.117.300120 29084818 PMC5714482

[42] Sharp NP, Smith DR, Driscoll G, Sun K, Vickerman CM, Martin SCT. Contribution of spontaneous mutations to quantitative and molecular variation at the highly repetitive rDNA locus in yeast. Genome Biol Evol. 2023;15(10):evad179. doi: 10.1093/gbe/evad179 37847861 PMC10581546

[43] Funk LC, Zasadil LM, Weaver BA. Living in CIN: Mitotic infidelity and its consequences for tumor promotion and suppression. Developmental Cell. 2016. 10.1016/j.devcel.2016.10.023 PMC5204306 27997823

[44] Sheltzer JM, Blank HM, Pfau SJ, et al. Aneuploidy drives genomic instability in yeast. Science. 2011;333(6045):1026–30. 21852501 10.1126/science.1206412 PMC3278960

[45] Anders KR, Kudrna JR, Keller KE, et al. A strategy for constructing aneuploid yeast strains by transient nondisjunction of a target chromosome. BMC Genetics. 2009;10(July):36. 19594932 10.1186/1471-2156-10-36 PMC2725114

[46] Espinet C, de la Torre MA, Aldea M, Herrero E. An efficient method to isolate yeast genes causing overexpression-mediated growth arrest. Yeast. 1995;11(1):25–32. doi: 10.1002/yea.320110104 7762298

[47] Deutschbauer AM, Jaramillo DF, Proctor M, Kumm J, Hillenmeyer ME, Davis RW, et al. Mechanisms of haploinsufficiency revealed by genome-wide profiling in yeast. Genetics. 2005;169(4):1915–25. doi: 10.1534/genetics.104.036871 15716499 PMC1449596

[48] Kozmin SG, Dominska M, Zheng D-Q, Petes TD. Splitting the yeast centromere by recombination. Nucleic Acids Res. 2024;52(2):690–707. doi: 10.1093/nar/gkad1110 37994724 PMC10810202

[49] Lopez V, Barinova N, Onishi M, Pobiega S, Pringle JR, Dubrana K, et al. Cytokinesis breaks dicentric chromosomes preferentially at pericentromeric regions and telomere fusions. Genes Dev. 2015;29(3):322–36. doi: 10.1101/gad.254664.114 25644606 PMC4318148

[50] McClintock B. The Stability of broken ends of chromosomes in zea mays. Genetics. 1941;26(2):234–82. doi: 10.1093/genetics/26.2.234 17247004 PMC1209127

[51] Guérin TM, Marcand S. Breakage in breakage-fusion-bridge cycle: An 80-year-old mystery. Trends Genet. 2022;38(7):641–5. 35397934 10.1016/j.tig.2022.03.008

[52] Song W, Gawel M, Dominska M, Greenwell PW, Hazkani-Covo E, Bloom K, et al. Nonrandom distribution of interhomolog recombination events induced by breakage of a dicentric chromosome in Saccharomyces cerevisiae. Genetics. 2013;194(1):69–80. doi: 10.1534/genetics.113.150144 23410835 PMC3632482

[53] Cook D, Kozmin SG, Yeh E, Petes TD, Bloom K. Dicentric chromosomes are resolved through breakage and repair at their centromeres. Chromosoma. 2024;133(2):117–34. doi: 10.1007/s00412-023-00814-6 38165460 PMC11180013

[54] Chan K-L, North PS, Hickson ID. BLM is required for faithful chromosome segregation and its localization defines a class of ultrafine anaphase bridges. EMBO J. 2007;26(14):3397–409. doi: 10.1038/sj.emboj.7601777 17599064 PMC1933408

[55] Finardi A, Massari LF, Visintin R. Anaphase bridges: Not all natural fibers are healthy. Genes. 2020;11(8):902. 32784550 10.3390/genes11080902 PMC7464157

[56] Quevedo O, García-Luis J, Matos-Perdomo E, Aragón L, Machín F. Nondisjunction of a single chromosome leads to breakage and activation of DNA damage checkpoint in G2. PLoS Genet. 2012;8(2):e1002509. doi: 10.1371/journal.pgen.1002509 22363215 PMC3280967

[57] Uhlmann F, Lottspeich F, Nasmyth K. Sister-chromatid separation at anaphase onset is promoted by cleavage of the cohesin subunit Scc1. Nature. 1999;400(6739):37–42. doi: 10.1038/21831 10403247

[58] Holm C, Goto T, Wang JC, Botstein D. DNA topoisomerase II is required at the time of mitosis in yeast. Cell. 1985;41(2):553–63. doi: 10.1016/s0092-8674(85)80028-3 2985283

[59] Holm C, Stearns T, Botstein D. DNA topoisomerase II must act at mitosis to prevent nondisjunction and chromosome breakage. Mol Cell Biol. 1989;9(1):159–68. doi: 10.1128/mcb.9.1.159-168.1989 2538717 PMC362157

[60] Ivanova T, Maier M, Missarova A, Ziegler-Birling C, Dam M, Gomar-Alba M, et al. Budding yeast complete DNA synthesis after chromosome segregation begins. Nat Commun. 2020;11(1):2267. doi: 10.1038/s41467-020-16100-3 32385287 PMC7210879

[61] Chan YW, Fugger K, West SC. Unresolved recombination intermediates lead to ultra-fine anaphase bridges, chromosome breaks and aberrations. Nat Cell Biol. 2018;20(1):92–103. doi: 10.1038/s41556-017-0011-1 29255170 PMC5742284

[62] Vasan S, Deem A, Ramakrishnan S, Argueso JL, Malkova A. Cascades of genetic instability resulting from compromised break-induced replication. PLoS Genet. 2014;10(2):e1004119. doi: 10.1371/journal.pgen.1004119 24586181 PMC3937135

[63] Tsaponina O, Haber JE. Frequent interchromosomal template switches during gene conversion in S. cerevisiae. Mol Cell. 2014;55(4):615–25. doi: 10.1016/j.molcel.2014.06.025 25066232 PMC4150392

[64] Yeh E, Yang C, Chin E, et al. Dynamic positioning of mitotic spindles in yeast: role of microtubule motors and cortical determinants. Molecular Biology of the Cell. 2000;11(11):3949–61. 11071919 10.1091/mbc.11.11.3949 PMC15049

[65] Li YY, Yeh E, Hays T, Bloom K. Disruption of mitotic spindle orientation in a yeast dynein mutant. Proc Natl Acad Sci U S A. 1993;90(21):10096–100. doi: 10.1073/pnas.90.21.10096 8234262 PMC47720

[66] Palmer RE, Sullivan DS, Huffaker T, Koshland D. Role of astral microtubules and actin in spindle orientation and migration in the budding yeast, Saccharomyces cerevisiae. J Cell Biol. 1992;119(3):583–93. doi: 10.1083/jcb.119.3.583 1400594 PMC2289680

[67] Smukowski Heil CS, DeSevo CG, Pai DA, Tucker CM, Hoang ML, Dunham MJ. Loss of heterozygosity drives adaptation in hybrid yeast. Mol Biol Evol. 2017;34(7):1596–612. doi: 10.1093/molbev/msx098 28369610 PMC5455960

[68] Fisher KJ, Buskirk SW, Vignogna RC, Marad DA, Lang GI. Adaptive genome duplication affects patterns of molecular evolution in Saccharomyces cerevisiae. PLoS Genet. 2018;14(5):e1007396. doi: 10.1371/journal.pgen.1007396 29799840 PMC5991770

[69] James TY, Michelotti LA, Glasco AD, Clemons RA, Powers RA, James ES, et al. Adaptation by loss of heterozygosity in saccharomyces cerevisiae clones under divergent selection. Genetics. 2019;213(2):665–83. doi: 10.1534/genetics.119.302411 31371407 PMC6781901

[70] Musacchio A, Salmon ED. The spindle-assembly checkpoint in space and time. Nat Rev Mol Cell Biol. 2007;8(5):379–93. doi: 10.1038/nrm2163 17426725

[71] Musacchio A n d r e a. The molecular biology of spindle assembly checkpoint signaling dynamics. Current Biology: CB. 2015. 10.1016/j.cub.2015.08.051 26485365

[72] Tanaka K, Mukae N, Dewar H. Molecular mechanisms of kinetochore capture by spindle microtubules. Nature. 2005. 10.1038/nature03483 15846338

[73] Pearson CG, Maddox PS, Salmon ED, Bloom K. Budding yeast chromosome structure and dynamics during mitosis. J Cell Biol. 2001;152(6):1255–66. doi: 10.1083/jcb.152.6.1255 11257125 PMC2199205

[74] Palme J, Wang J, Springer M. Variation in the modality of a yeast signaling pathway is mediated by a single regulator. Elife. 2021;10:e69974. doi: 10.7554/eLife.69974 34369878 PMC8373380

[75] Merlo LMF, Pepper JW, Reid BJ, Maley CC. Cancer as an evolutionary and ecological process. Nat Rev Cancer. 2006;6(12):924–35. doi: 10.1038/nrc2013 17109012

[76] Bakker B, Taudt A, Belderbos ME, Porubsky D, Spierings DCJ, de Jong TV, et al. Single-cell sequencing reveals karyotype heterogeneity in murine and human malignancies. Genome Biol. 2016;17(1):115. doi: 10.1186/s13059-016-0971-7 27246460 PMC4888588

[77] Gao R, Davis A, McDonald TO, Sei E, Shi X, Wang Y, et al. Punctuated copy number evolution and clonal stasis in triple-negative breast cancer. Nat Genet. 2016;48(10):1119–30. doi: 10.1038/ng.3641 27526321 PMC5042845

[78] Casasent AK, Schalck A, Gao R, Sei E, Long A, Pangburn W, et al. Multiclonal invasion in breast tumors identified by topographic single cell sequencing. Cell. 2018;172(1–2):205-217.e12. doi: 10.1016/j.cell.2017.12.007 29307488 PMC5766405

[79] Bollen Y, Stelloo E, van Leenen P, van den Bos M, Ponsioen B, Lu B, et al. Reconstructing single-cell karyotype alterations in colorectal cancer identifies punctuated and gradual diversification patterns. Nat Genet. 2021;53(8):1187–95. doi: 10.1038/s41588-021-00891-2 34211178 PMC8346364

[80] Starostik MR, Sosina OA, McCoy RC. Single-cell analysis of human embryos reveals diverse patterns of aneuploidy and mosaicism. Genome Res. 2020;30(6):814–25. doi: 10.1101/gr.262774.120 32641298 PMC7370883

[81] Altemose N, Logsdon GA, Bzikadze AV, Sidhwani P, Langley SA, Caldas GV, et al. Complete genomic and epigenetic maps of human centromeres. Science. 2022;376(6588):eabl4178. doi: 10.1126/science.abl4178 35357911 PMC9233505

[82] Salinas-Luypaert C, Dubocanin D, Lee RJ, Andrade Ruiz L, Gamba R, Grison M, et al. DNA methylation influences human centromere positioning and function. Nat Genet. 2025;57(10):2509–21. doi: 10.1038/s41588-025-02324-w 40908343 PMC12513831

[83] Henikoff S, Henikoff JG. “Point” centromeres of Saccharomyces harbor single centromere-specific nucleosomes. Genetics. 2012;190(4):1575–7. doi: 10.1534/genetics.111.137711 22234856 PMC3316665

[84] Krassovsky K, Henikoff JG, Henikoff S. Tripartite organization of centromeric chromatin in budding yeast. Proc Natl Acad Sci U S A. 2012;109(1):243–8. doi: 10.1073/pnas.1118898109 22184235 PMC3252899

[85] Bensasson D. Evidence for a high mutation rate at rapidly evolving yeast centromeres. BMC Evol Biol. 2011;11:211. doi: 10.1186/1471-2148-11-211 21767380 PMC3155921

[86] Helsen J, Ramachandran K, Sherlock G, Dey G. Progressive coevolution of the yeast centromere and kinetochore. Nature. 2025. 10.1038/s41586-025-09779-1 PMC12925627 41299172

[87] Duan Z, Andronescu M, Schutz K, McIlwain S, Kim YJ, Lee C, et al. A three-dimensional model of the yeast genome. Nature. 2010;465(7296):363–7. doi: 10.1038/nature08973 20436457 PMC2874121

[88] Costantino L, Hsieh T-HS, Lamothe R, Darzacq X, Koshland D. Cohesin residency determines chromatin loop patterns. Elife. 2020;9:e59889. doi: 10.7554/eLife.59889 33170773 PMC7655110

[89] Kim S, Liachko I, Donna G, et al. The Dynamic Three-Dimensional Organization of the Diploid Yeast Genome. 2017. 10.7554/eLife.23623 PMC5476426 28537556

[90] Chan KL, Palmai-Pallag T, Ying S, Hickson ID. Replication stress induces sister-chromatid bridging at fragile site loci in mitosis. Nat Cell Biol. 2009;11(6):753–60. doi: 10.1038/ncb1882 19465922

[91] Naim V, Wilhelm T, Debatisse M, Rosselli F. ERCC1 and MUS81-EME1 promote sister chromatid separation by processing late replication intermediates at common fragile sites during mitosis. Nat Cell Biol. 2013;15(8):1008–15. doi: 10.1038/ncb2793 23811686

[92] Wang LH-C, Schwarzbraun T, Speicher MR, Nigg EA. Persistence of DNA threads in human anaphase cells suggests late completion of sister chromatid decatenation. Chromosoma. 2008;117(2):123–35. doi: 10.1007/s00412-007-0131-7 17989990 PMC2755729

[93] Wang LH-C, Mayer B, Stemmann O, Nigg EA. Centromere DNA decatenation depends on cohesin removal and is required for mammalian cell division. J Cell Sci. 2010;123(Pt 5):806–13. doi: 10.1242/jcs.058255 20144989

[94] Chan YW, West SC. A new class of ultrafine anaphase bridges generated by homologous recombination. Cell Cycle. 2018;17(17):2101–9. 30253678 10.1080/15384101.2018.1515555 PMC6226235

[95] Sarbajna S, Davies D, West SC. Roles of SLX1-SLX4, MUS81-EME1, and GEN1 in avoiding genome instability and mitotic catastrophe. Genes & Development. 2014;28(10):1124–36. 24831703 10.1101/gad.238303.114 PMC4035540

[96] Naim V, Rosselli F. The FANC pathway and BLM collaborate during mitosis to prevent micro-nucleation and chromosome abnormalities. Nat Cell Biol. 2009;11(6):761–8. doi: 10.1038/ncb1883 19465921

[97] Norden C, Mendoza M, Dobbelaere J, Kotwaliwale CV, Biggins S, Barral Y. The NoCut pathway links completion of cytokinesis to spindle midzone function to prevent chromosome breakage. Cell. 2006;125(1):85–98. doi: 10.1016/j.cell.2006.01.045 16615892

[98] Amaral N, Vendrell A, Funaya C, et al. The Aurora-B-Dependent NoCut checkpoint prevents damage of anaphase bridges after DNA replication stress. Nat Cell Biol. 2016;18(5):516–26. 27111841 10.1038/ncb3343

[99] Stewénius Y, Gorunova L, Jonson T, Larsson N, Höglund M, Mandahl N, et al. Structural and numerical chromosome changes in colon cancer develop through telomere-mediated anaphase bridges, not through mitotic multipolarity. Proc Natl Acad Sci U S A. 2005;102(15):5541–6. doi: 10.1073/pnas.0408454102 15809428 PMC556242

[100] D’Amours D, Stegmeier F, Amon A. Cdc14 and condensin control the dissolution of cohesin-independent chromosome linkages at repeated DNA. Cell. 2004;117(4):455–69. doi: 10.1016/s0092-8674(04)00413-1 15137939

[101] Machín F, Torres-Rosell J, De Piccoli G, Carballo JA, Cha RS, Jarmuz A, et al. Transcription of ribosomal genes can cause nondisjunction. J Cell Biol. 2006;173(6):893–903. doi: 10.1083/jcb.200511129 16769819 PMC2063915

[102] Potapova TA, Unruh JR, Yu Z, Rancati G, Li H, Stampfer MR, et al. Superresolution microscopy reveals linkages between ribosomal DNA on heterologous chromosomes. J Cell Biol. 2019;218(8):2492–513. doi: 10.1083/jcb.201810166 31270138 PMC6683752

[103] Van Rossum G, Drake FL. Python 3 Reference Manual. CreateSpace. 2009.

[104] Virtanen P, Gommers R, Oliphant TE, Haberland M, Reddy T, Cournapeau D, et al. SciPy 1.0: Fundamental algorithms for scientific computing in Python. Nat Methods. 2020;17(3):261–72. doi: 10.1038/s41592-019-0686-2 32015543 PMC7056644

[105] Chen S, Zhou Y, Chen Y, Gu J. Fastp: An ultra-fast all-in-one FASTQ preprocessor. Bioinformatics. 2018;34(17):i884–90. 10.1093/bioinformatics/bty560 PMC6129281 30423086

[106] Vasimuddin Md, Misra S, Li H, Aluru S. Efficient Architecture-Aware Acceleration of BWA-MEM for Multicore Systems. 2019 IEEE International Parallel and Distributed Processing Symposium (IPDPS), 2019. 314–24. doi: 10.1109/ipdps.2019.00041

[107] Li H, Handsaker B, Wysoker A, et al. The Sequence Alignment/Map format and SAMtools. Bioinformatics. 2009;25(16):2078–9. 19505943 10.1093/bioinformatics/btp352 PMC2723002

[108] Li H. A statistical framework for SNP calling, mutation discovery, association mapping and population genetical parameter estimation from sequencing data. Bioinformatics. 2011;27(21):2987–93. doi: 10.1093/bioinformatics/btr509 21903627 PMC3198575

[109] Sarkar S. Haldane’s solution of the Luria-Delbrück distribution. Genetics. 1991;127(2):257–61. doi: 10.1093/genetics/127.2.257 2004702 PMC1204353

[110] Köster J, Rahmann S. Snakemake--a scalable bioinformatics workflow engine. Bioinformatics. 2012;28(19):2520–2. doi: 10.1093/bioinformatics/bts480 22908215

[111] Schindelin J, Arganda-Carreras I, Frise E, Kaynig V, Longair M, Pietzsch T, et al. Fiji: An open-source platform for biological-image analysis. Nat Methods. 2012;9(7):676–82. doi: 10.1038/nmeth.2019 22743772 PMC3855844

