# Supplementary figures and images for: Towards a unified model of aneuploid karyotype dynamics
Source: PLoS Genet. 2026 Jun 18;22(6):e1012210. doi: 10.1371/journal.pgen.1012210 (PMC13318051; doi:10.1371/journal.pgen.1012210)

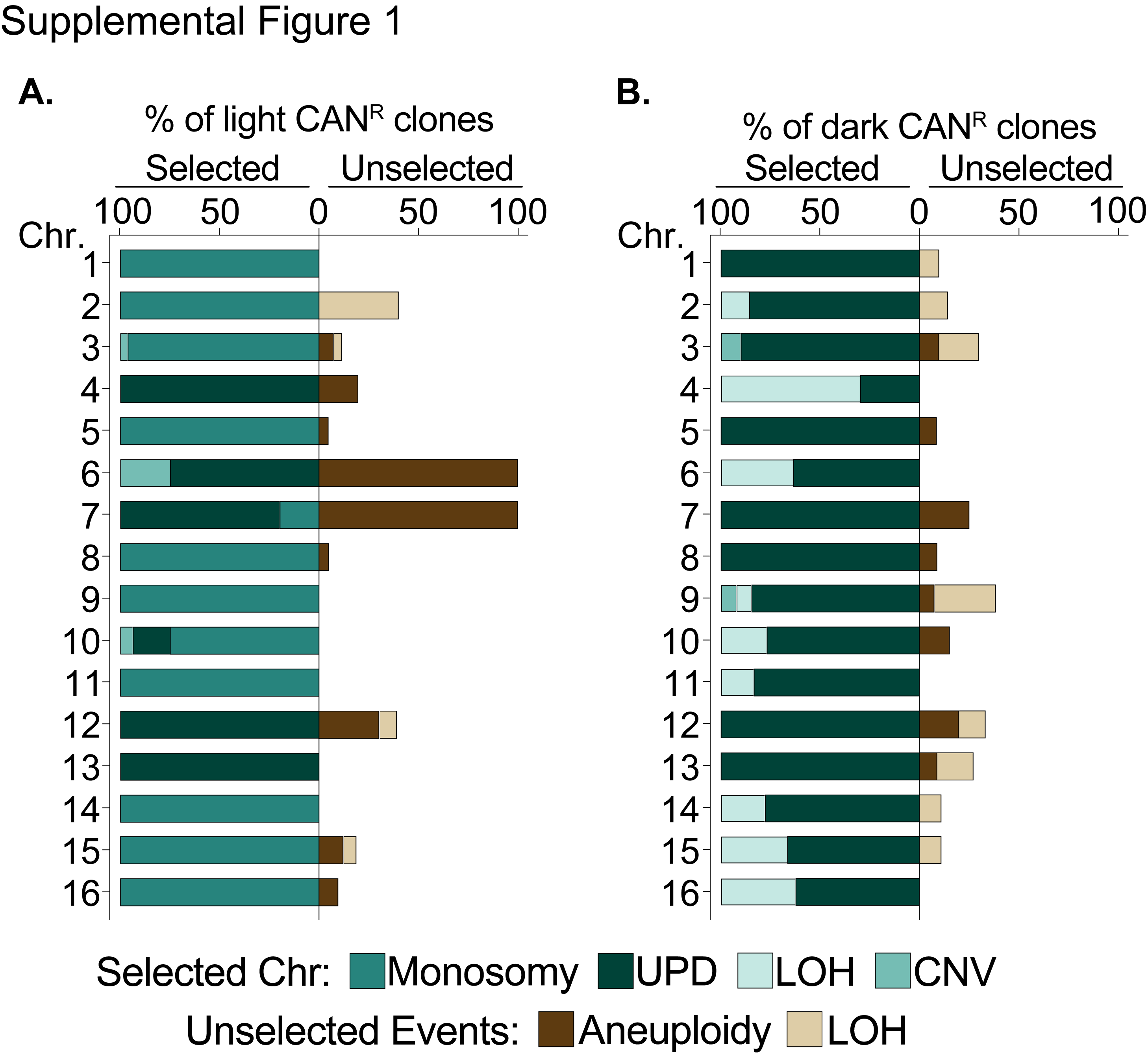

Supplement: S1 Fig — A., B. Quantification of the percentage of light CANR (A) and dark CANR (B) clones that harbored the denoted karyotypic states affecting the predicted chromosome (Selected) as well as any additional unselected events (Unselected). (TIF) [file pgen.1012210.s001.tif]

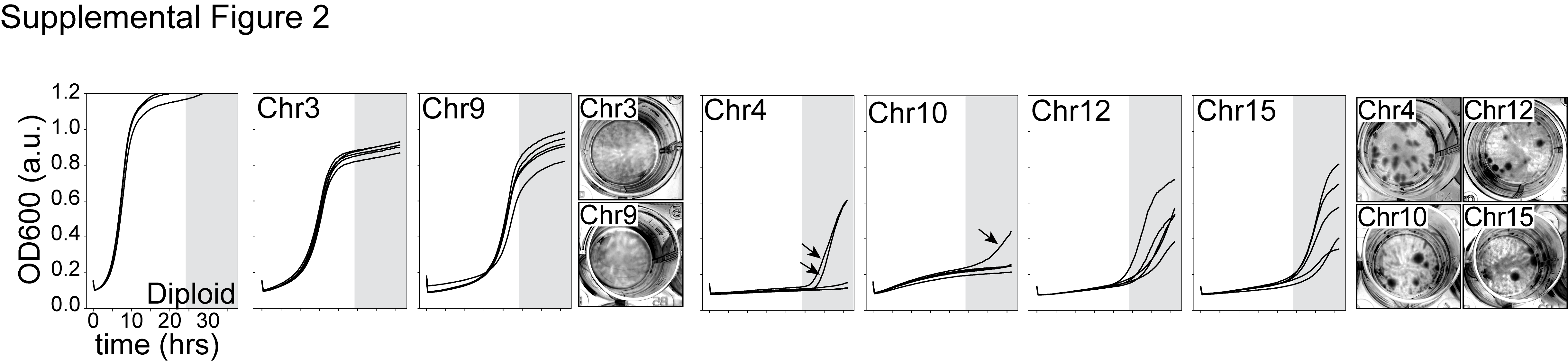

Supplement: S2 Fig — OD600 growth curve data and end point microwell images of representative strains subjected to the induced monosome generation fitness assays. Black arrowheads point to late-stage, rapid, increases in growth rate indicative of emergent revertant subpopulations. Grey boxes in each plot denote the growth kinetics data that were excluded from our estimate of growth rate to eliminate revertant emergence as a confounding factor. (TIF) [file pgen.1012210.s002.tif]

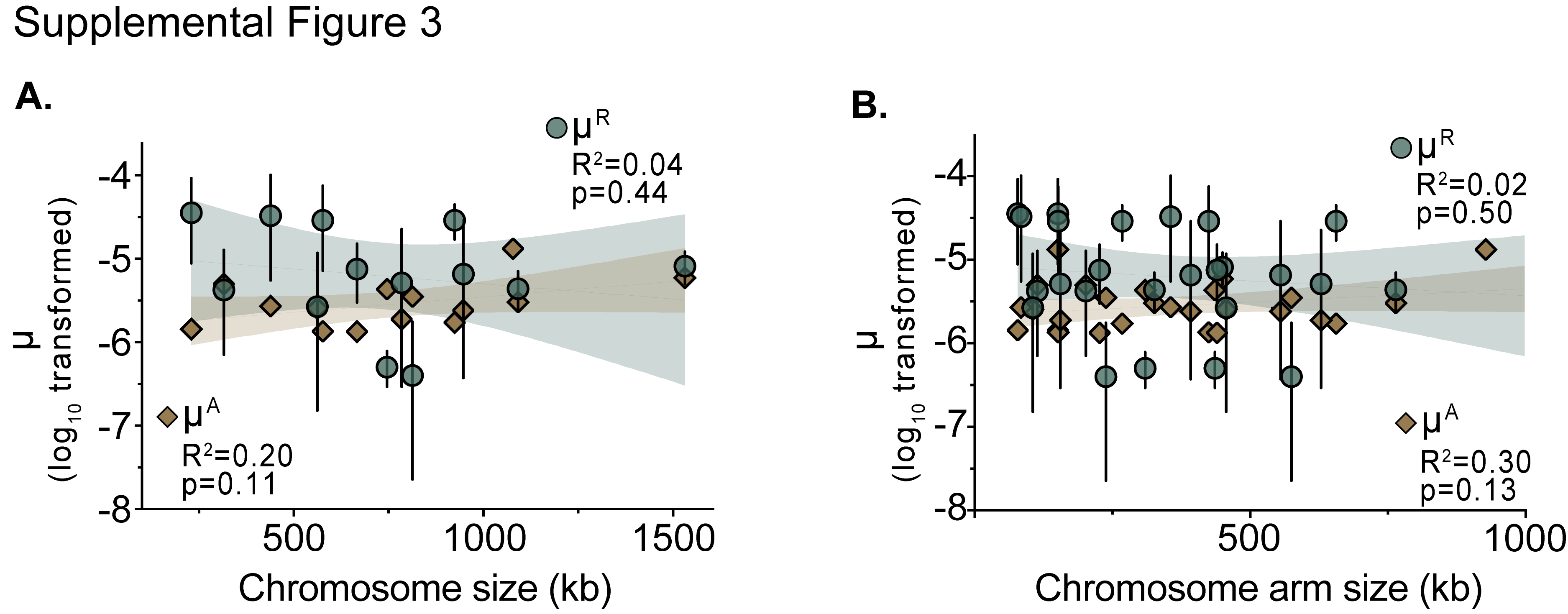

Supplement: S3 Fig — Log-transformed per-chromosome estimates of the rates of monosomy and reversion plotted by chromosome size (A.) or the length of each chromosome arm (B.). Vertical error bars denote the 95% confidence intervals associated with each per-chromosome estimate of μA or μR. Brown and green areas depict the 95% confidence intervals produced from the simple linear regression of μA (brown) μR (green) or vs. chromosome size. (TIF) [file pgen.1012210.s003.tif]

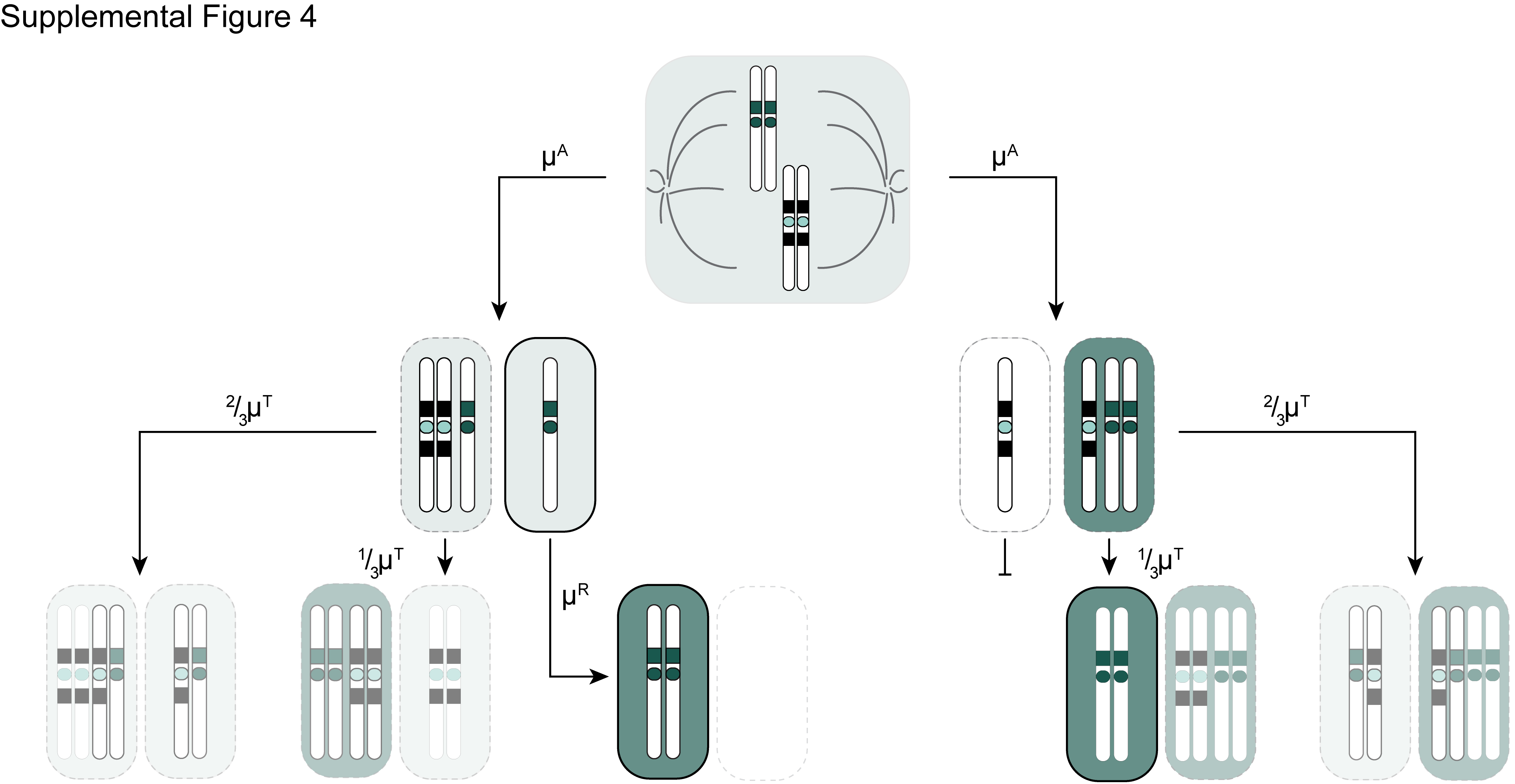

Supplement: S4 Fig — A schematic illustrating genotypes, phenotypes, and rates associated with nondisjunction events that result in the formation and subsequent reversion of monosomic and trisomic daughter cell pairs. Symbols are the same as in Fig 1. Only the derivatives outlined with a solid line would be recoverable in the canavanine fluctuation tests. (TIF) [file pgen.1012210.s004.tif]

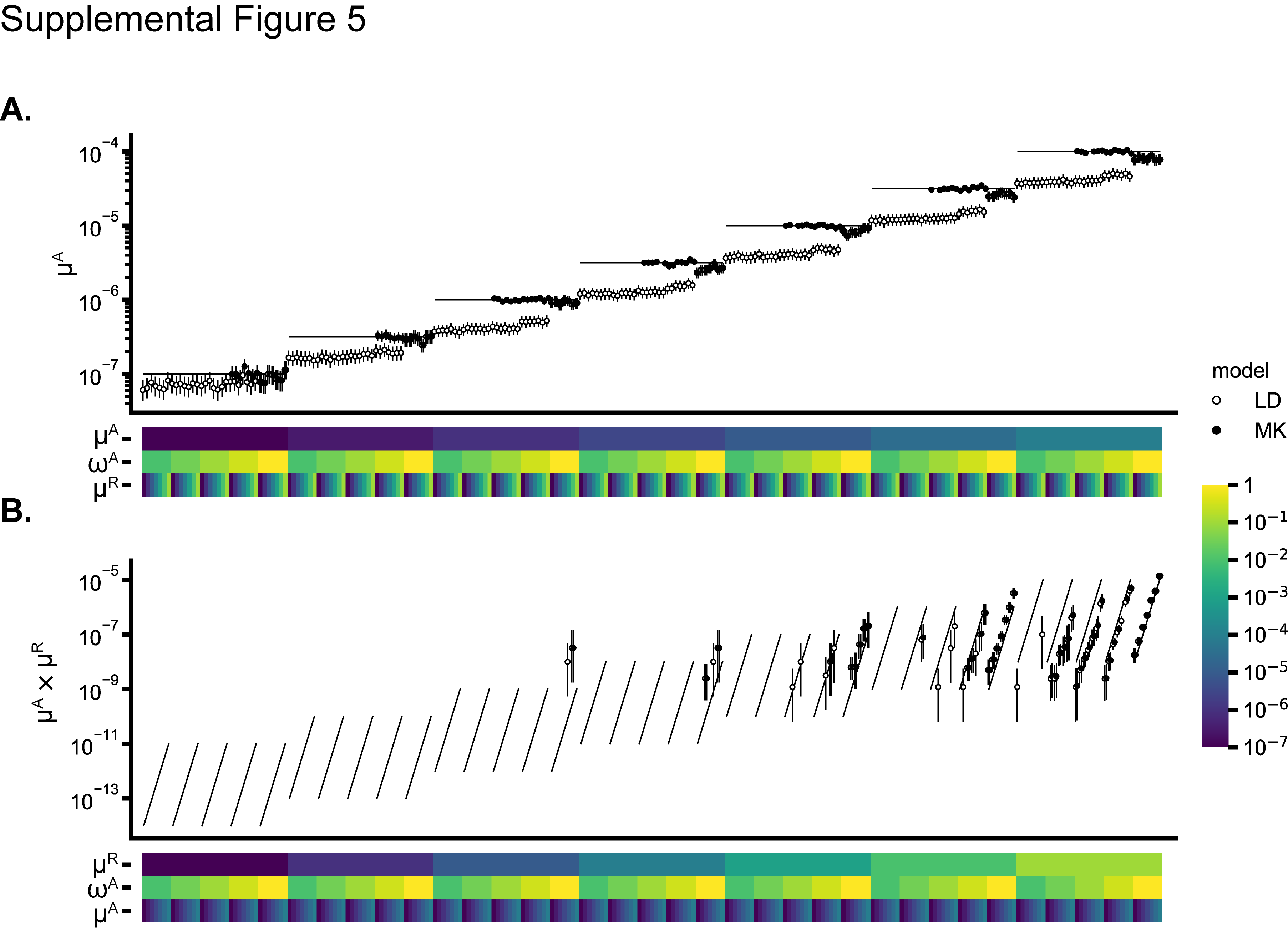

Supplement: S5 Fig — Mutation rates (dots) and 95% confidence intervals (vertical lines) were estimated from simulated mutant counts using rSalvador [41]. We performed 245 simulations across all combinations of seven μA values (10-7–10-4), seven μR values (10-7–10-1) and five ωA values (10-2–100). For these validation simulations, we ignored reversion that occurs via the trisomic counterparts of monosomy events, setting μT=0 and ωT=1. We performed 50 replicates for each parameter combination. Two models were fitted on simulated mutant counts, the standard Luria-Delbrück model (Lea-Coulson, open dots) and the model accounting for variable mutant fitness (Mandelbrot-Koch, closed dots). The rate values expected from the input parameters are shown as thin background lines. Input parameters are summarized as heatmaps below each plot. Monosome rates (μA) were estimated from combined counts of monosomic and revertant mutants (A), and compound rates (μC = μA × μR) were estimated from revertant mutant counts alone (B). (TIF) [file pgen.1012210.s005.tif]

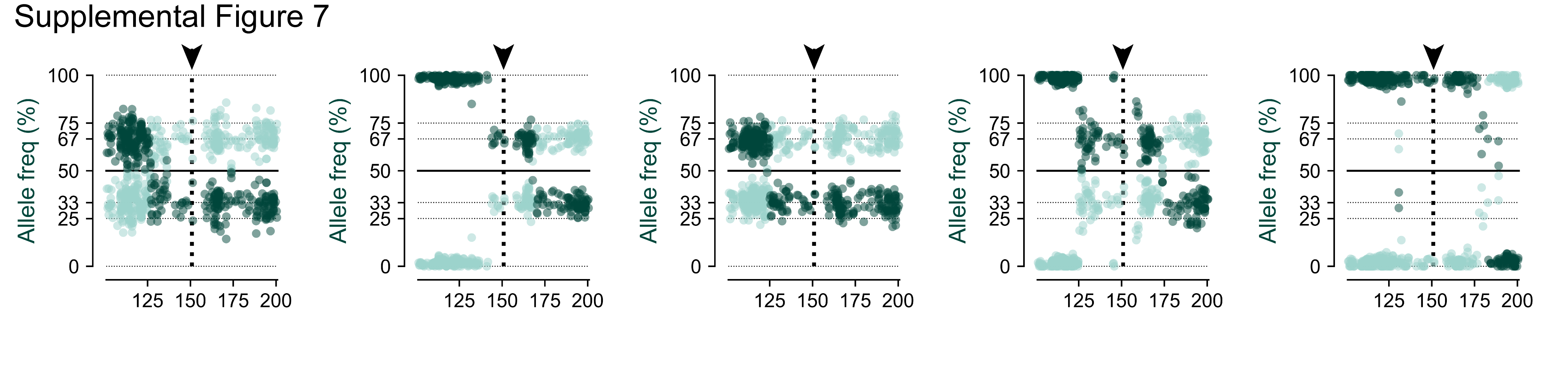

Supplement: S7 Fig — Chr12-specific whole-chromosome or centromere-proximal WGS mappings of the recovered trisomic CANR colonies. Black arrowheads denote the position of CEN12. (TIF) [file pgen.1012210.s007.tif]

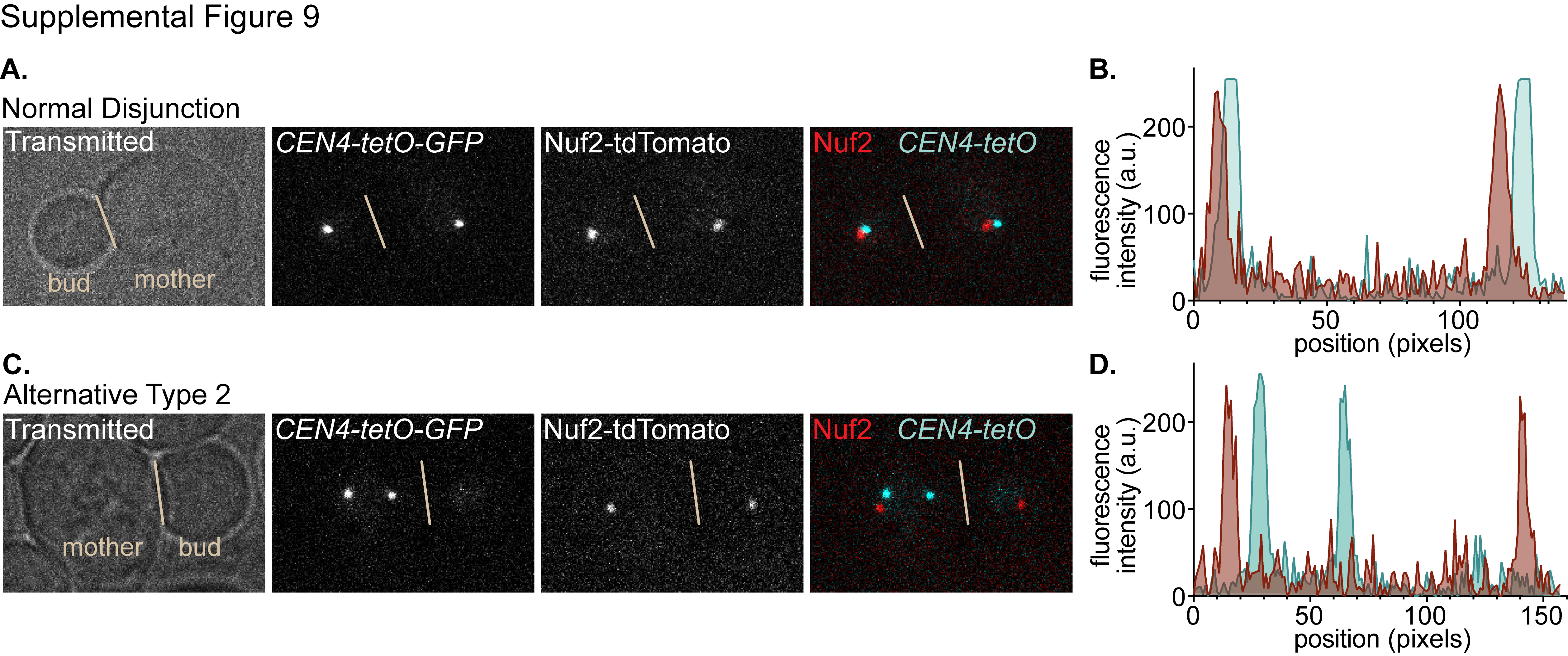

Supplement: S9 Fig — A. and C. Representative images of a normal disjunction event (A.) and an alternative Type 2 missegregation event (C.) occurring in URA3-GAL1p-CEN4-tetO cells expressing TetR-GFP and Nuf2-tdTomato, a kinetochore protein required for spindle microtubule attachment. B. and D. Corresponding line scan analysis illustrating the degree of TetR and Nuf2 signal overlap in A. and C.; Line scan analysis was performed by determining the pixel intensity across the distance spanning all four fluorescent foci for both detection channels using Fiji. (TIF) [file pgen.1012210.s009.tif]
