# Supplementary material for: Towards a unified model of aneuploid karyotype dynamics
Source: PLoS Genet. 2026 Jun 18;22(6):e1012210. doi: 10.1371/journal.pgen.1012210 (PMC13318051; doi:10.1371/journal.pgen.1012210)
Supplement: S2 Appendix — Additional discussion points regarding the biological contexts for modeling aneuploidy dynamics. (DOCX) [file pgen.1012210.s017.docx]

**S2 Appendix:**

On the possible contribution of non-reciprocal asymmetric aneuploidy to karyotypic dynamics in populations: Several key differences between yeast and mammalian mitosis lead us to hypothesize that the asymmetric aneuploidy generated from canonical anaphase chromosome lag is a negligible effector of the aneuploid karyotype spectra and frequencies observed in yeast populations. First, lagging chromosomes in mammalian cells generally arise from merotelic attachments [(Cimini et al. 2001)](https://paperpile.com/c/DTalbn/wcR2), in which microtubules emanating from both spindle poles engage the kinetochore of a single chromatid. Because yeast point centromeres assemble kinetochores that can only engage a single microtubule [(Winey et al. 1995)](https://paperpile.com/c/DTalbn/Abat), merotelic attachments are not thought to be possible, and to our knowledge, have never been observed in yeast. Second, the ‘loss’ of lagging chromosomes is generally attributed to their exclusion from the reforming nuclear compartments. Due to their distance from the primary chromatin masses—which recruit nuclear envelope reformation machinery like the BAF and LEM protein complexes [(Samwer et al. 2017; von Appen et al. 2020)](https://paperpile.com/c/DTalbn/Gi1R+XaC1)—lagging chromosomes either form separate micronuclei or remain exposed in the interphase cytoplasm, resulting in their loss or degradation [(Hatch et al. 2013)](https://paperpile.com/c/DTalbn/JEqc). Because yeast undergo closed mitosis, even chromatin that lags in the spindle midzone (*i.e.*, the rDNA array) remains encapsulated within the intact nucleus, greatly reducing the chances of its isolation from the dividing nuclear compartment [(Quevedo et al. 2012)](https://paperpile.com/c/DTalbn/Nnb0). It is worth noting, however, that our live-cell imaging studies of *CEN4* (Fig 8C) captured an event in which resolution of a lagging centromere resulted in the formation of a separated ‘micronuclear-like’ compartment that was never reabsorbed into the primary nucleus. This observation indicates that asymmetric loss of DNA does occur in yeast, though the underlying cellular events that lead to this outcome have not been well-described. In addition, though our model of linkage-mediated nondisjunction and recombinant reversion is presented as a symmetrical event, the relative repair efficiencies of the reciprocally inherited chromosome fragments has not been demonstrated.

We have considered how lag-mediated events (asymmetric) would impact the monosome and revertant dynamics described in our model. Experimentally, monosomy arising from both canonical symmetric events and asymmetric events are encompassed in our existing estimates of $\mu$^A^, such that we can define the compound rate of monosomy as: $\mu$^A*^ = $\mu$^A^ + $\mu$^L^, where $\mu$^A^ is the rate of symmetrical monosomy and $\mu$^L^ is the rate of asymmetrical monosomy. $\mu$^A^ and $\mu$^L^ would generate indistinguishable monosomic CAN^R^ cells (Fig. S4) with equal probabilities of reverting ($\mu$^R^). However, whereas symmetric events also generate a trisomic daughter cell capable of reverting ($\mu$^T^), asymmetric events would generate a daughter retaining the parental karyotype which would confer a negligible contribution to subsequent population dynamics. As such, if $\mu$^L^ >> $\mu$^A^, the contribution of trisomy-mediated reversion ($\mu$^T^) would be reduced in the population because the ratio of monosomes and trisomes generated would be nonequivalent (A>>T). Without accounting for asymmetric events, our models demonstrated that $\mu$^T^ would need to be extraordinarily high to explain the observed frequency of revertants in our fluctuation test data; if $\mu$^L^ >> $\mu$^A^, this would necessitate that trisome-mediated reversion be an essentially deterministic event, which it is not. Thus, though our experimental data do not explicitly disprove the possibility that $\mu$^L^ >> $\mu$^A^—and thus that $\mu$^A^ << $\mu$^R^— this possibility does not change our primary conclusion that reversion through stable aneuploid states is insufficient to explain the observed frequency of revertant cells in the populations we sampled.

On the scalability of the model as a function of ploidy: Organisms vary widely at the level of base ploidy, and thus the occurrence of aneuploidy in these different ploidy contexts is expected to vary [(Sharp et al. 2018; Peter et al. 2018; Selmecki et al. 2015)](https://paperpile.com/c/DTalbn/MrsF+EmPc+rt6I). We considered whether the probability of nondisjunction in a given cell division ought to scale with the current number of chromosome copies. All rates being equal, reversion from trisomy to disomy is 3 times more likely to occur than reversion from monosomy to disomy (Fig. S2), though only 1/3 of reversion events would result in the uniparental disomy assessed in the fluctuation assays. From a purely probabilistic standpoint, the increased likelihood of nondisjunction ought to scale with chromosome copy number. From an empirical standpoint, previous studies describing ploidy-dependent differences in chromosome loss rates have indicated that ploidy is negatively correlated with genomic instability, manifesting as higher rates of chromosome loss [(Mayer and Aguilera 1990; Kumaran et al. 2013; Dutta et al. 2022)](https://paperpile.com/c/DTalbn/iCp2+uBZp+Qahj). However, with a few notable exceptions, the increased frequencies of aneuploidy observed in cells of higher ploidy are vulnerable caveats noted in the introduction of our manuscript.

On CIN as it relates to linkage-mediated nondisjunction: CIN phenotypes can manifest broad mutational spectra. The CIN displayed in the sequenced clones of Chr6 and Chr7 is generally attributed to defects in microtubule assembly and stability and is thought to manifest in increased rates of simple nondisjunction events. However, we have also considered the possibility that it might also result in an increase in linkage-mediated nondisjunction typified by centromere-proximal breakpoints. As a preliminary test of this hypothesis, we assessed the spectra of unselected aneuploidies—simple or recombinant—harbored by the light CAN^R^ clones of Chr6 and Chr7. All of the unselected aneuploidies detected were simple; we detected no examples of recombinant aneuploidy. Only 3 unselected recombination tracts were detected amongst the clones: 2/3 events were mosaic terminal LOH tracts with the breakpoint arising in the ribosomal DNA; the other event constituted a terminal LOH tract on the right arm of Chr8 with the breakpoint near the subtelomere. The co-occurrence of rDNA-originating LOH could suggest that the CIN phenotype displayed by these clones increases linkage-mediated nondisjunction. However, this is a speculation that requires further study.

**S2 Appendix References:**

[Appen, Alexander von, Dollie LaJoie, Isabel E. Johnson, et al. 2020. “LEM2 Phase Separation Promotes ESCRT-Mediated Nuclear Envelope Reformation.” *Nature* 582 (7810): 115–118.](http://paperpile.com/b/DTalbn/XaC1)

[Cimini, D., B. Howell, P. Maddox, A. Khodjakov, F. Degrassi, and E. D. Salmon. 2001. “Merotelic Kinetochore Orientation Is a Major Mechanism of Aneuploidy in Mitotic Mammalian Tissue Cells.” *The Journal of Cell Biology* 153 (3): 517–527.](http://paperpile.com/b/DTalbn/wcR2)

[Hatch, Emily M., Andrew H. Fischer, Thomas J. Deerinck, and Martin W. Hetzer. 2013. “Catastrophic Nuclear Envelope Collapse in Cancer Cell Micronuclei.” *Cell* 154 (1): 47–60.](http://paperpile.com/b/DTalbn/JEqc)

[Mayer, V. W., and A. Aguilera. 1990. “High Levels of Chromosome Instability in Polyploids of Saccharomyces Cerevisiae.” *Mutation Research* 231 (2): 177–186.](http://paperpile.com/b/DTalbn/iCp2)

[Samwer, Matthias, Maximilian W. G. Schneider, Rudolf Hoefler, et al. 2017. “DNA Cross-Bridging Shapes a Single Nucleus from a Set of Mitotic Chromosomes.” *Cell* 170 (5): 956–972.e23.](http://paperpile.com/b/DTalbn/Gi1R)

[Selmecki, A. M., Y. E. Maruvka, P. A. Richmond, et al. 2015. “Polyploidy Can Drive Rapid Adaptation in Yeast.” *Nature*, March.](http://paperpile.com/b/DTalbn/rt6I) <https://www.ncbi.nlm.nih.gov/pubmed/25731168>[.](http://paperpile.com/b/DTalbn/rt6I)

[Sharp, Nathaniel P., Linnea Sandell, Christopher G. James, and Sarah P. Otto. 2018. “The Genome-Wide Rate and Spectrum of Spontaneous Mutations Differ between Haploid and Diploid Yeast.” *Proceedings of the National Academy of Sciences of the United States of America* 115 (22): E5046–E5055.](http://paperpile.com/b/DTalbn/MrsF)

[Winey, M., C. L. Mamay, E. T. O’Toole, et al. 1995. “Three-Dimensional Ultrastructural Analysis of the Saccharomyces Cerevisiae Mitotic Spindle.” *The Journal of Cell Biology* 129 (6): 1601–1615.](http://paperpile.com/b/DTalbn/Abat)
