# Supplementary material for: Towards a unified model of aneuploid karyotype dynamics
Source: PLoS Genet. 2026 Jun 18;22(6):e1012210. doi: 10.1371/journal.pgen.1012210 (PMC13318051; doi:10.1371/journal.pgen.1012210)
Supplement: Attachment — Submitted filename: Response to Reviewers.pdf [file pgen.1012210.s019.pdf]

General Response to Reviewer Feedback:

We are grateful for the constructive and thoughtful feedback provided by the reviewers. In general we are pleased that the reviewers found the work sound and impactful. We are also glad that—despite the dense technical language required to explain the genetic and computational approaches—the main conclusions of the study were evident. The critiques raised by the reviewers offered meaningful clarity as to how to improve the article and we have incorporated these changes into the revised manuscript. Within the accompanying revised text file, new revisions are highlighted in green. They are directly addressed, point by point, in the following pages. These responses are also highlighted in green, with explicit changes emphasized with bold font.

Specific Responses to Reviewer Questions and Comments:

Reviewer #1:
1. In the introduction, it might be helpful to clarify that many of the studies cited used empirical approaches designed to minimize the influence of selection on the detection of aneuploidy and other mutants (as they should). For instance, in mutation accumulation (MA) experiments, it's certainly true that the possibility of aneuploidy reversion is generally not accounted for, and that differences in fitness could potentially confound rate estimates; however, even with these caveats, MA or fluctuation test style experiments will be a much more useful approaches than other types of experimental evolution where karyotypes evolve under selection, assuming our goal is to understand intrinsic mutation properties. The authors know all this, but in my experience many geneticists are not used to thinking about this distinction, and may assume that the karyotypic changes in question are under strong selection in these experiments.

This is a great point, one worth being explicit about in the introduction. **We've amended the revised text accordingly.**

2. It is known that classical nondisjunction, where a chromosome gain in one daughter cell is coupled with a chromosome loss in the other daughter cell, is not the only means through which aneuploidy might arise. In particular, chromosome loss could occur through "anaphase lag", where there is a loss without a corresponding gain. If this is happening in yeast, we might expect the rate of mutation to monosomy (which can occur through nondisjunction or chromosome lag) to exceed the rate of reversion, which can only occur through nondisjunction, all else being equal. I'd ask the authors to consider how the anaphase lag model might apply to their observations.

Several key differences between yeast and mammalian mitosis led us to hypothesize that the asymmetric aneuploidy generated from anaphase lag is a negligible effector of the aneuploid karyotype spectra and frequencies observed in yeast populations. First, lagging chromosomes generally arise from merotelic attachments (PMID:11331303), in which microtubules emanating from both spindle poles engage the kinetochore of a single chromatid. Because yeast kinetochores can only engage a single microtubule, merotelic attachments are not thought to be possible (PMID:7790357), and to our knowledge, have never been observed in yeast. Second, the 'loss' of lagging chromosomes is generally attributed to their exclusion from the reforming nuclear compartments. Due to their distance from the primary chromatin masses—which recruit nuclear envelope reformation machinery like the BAF and LEM protein complexes—lagging chromosomes form either separate micronuclei or remain exposed in the interphase cytoplasm, resulting in their loss or degradation. Because yeast undergo closed mitosis, even chromatin that lags in the spindle midzone (*i.e.*, the rDNA array is one such locus that does this frequently) remains encapsulated within the intact nucleus, greatly reducing the chances of its isolation from the dividing nuclear compartments. It is worth noting, however, that our live-cell imaging studies of *CEN4* (Figure 8C) captured an event in which resolution of a lagging centromere resulted in the formation of a separated 'micronuclear-like' compartment that was never reabsorbed into the primary nucleus. This observation indicates that asymmetric loss of DNA does occur in

yeast, though the underlying cellular events that lead to this outcome have not been well-described. In addition, though our model of linkage-mediated nondisjunction and recombinant reversion is presented as a symmetrical event, the relative repair efficiencies of the reciprocally inherited chromosome fragments has not been demonstrated. Biases in repair efficiency will impact the potential effects of asymmetric chromosome loss on aneuploid frequency and will certainly be a focus of our future studies.

We have, however, considered how lag-mediated events (asymmetrical) would impact the monosome and revertant dynamics described in our model. Experimentally, monosomy arising from both canonical symmetrical events and asymmetric events are encompassed in our existing estimates of μ^A, such that we can define the compound rate of monosomy as: $\mu^{A*} = \mu^A + \mu^L$, where μ^A is the rate of symmetrical monosomy and μ^L is the rate of asymmetrical monosomy. μ^A and μ^L would generate indistinguishable monosomic CAN^R cells (Fig. S4) with equal probabilities of reverting (μ^R). However, whereas symmetrical events also generate a trisomic daughter cell capable of reverting (μ^T), asymmetrical events would generate a daughter retaining the parental karyotype which would confer a negligible contribution to subsequent aneuploid population dynamics. As such, if $\mu^L >> \mu^A$, the contribution of trisomy-mediated reversion (μ^T) would be reduced in the population because the ratio of monosomes and trisomes generated would be nonequivalent (A>>T). Without accounting for asymmetric events, our models demonstrated that μ^T would need to be extraordinarily high to explain the observed frequency of revertants in our fluctuation test data; if $\mu^L >> \mu^A$, this would necessitate that trisome-mediated reversion be an essentially deterministic event, which it is not. Thus, though our experimental data do not explicitly disprove the possibility that $\mu^L >> \mu^A$—and thus that $\mu^A << \mu^R$— this possibility does not change our primary conclusion that reversion through stable aneuploid states is insufficient to explain the observed frequency of revertant cells in the populations we sampled.

We feel that including this discussion will add value to the manuscript and increase the relevance of our study to model systems in which lagging chromosome loss is well-documented. We have therefore provided it in a new supplemental document called 'Extended Discussion'.

3. As we move towards unbiased quantification of karyotype mutations, I wonder if the authors have considered whether the probability of nondisjunction in a given cell division ought to scale with the current number of chromosome copies. For example, all else being equal, the probability of segregation error occurring in a tetraploid should be twice that of a diploid, since tetraploids have to manage twice as many chromosomes overall. When thinking about aneuploidy reversion in diploids, this would suggest that reversion from trisomy to euploidy is more likely than reversion from monosomy to euploidy.

This logic is correct and is an integral parameter of the modelling studies we conducted. All rates being equal, reversion from trisomy to disomy is 3 times more likely to occur than reversion from monosomy to disomy (Fig. S4), though only 1/3 of reversion events would result in the UPD selected in the fluctuation assays. From a purely probabilistic standpoint, the increased likelihood of nondisjunction ought to scale with chromosome copy number. From an empirical standpoint, previous studies describing ploidy-dependent differences in chromosome loss rates have indicated that ploidy is negatively correlated with genomic instability, manifesting as higher rates of chromosome loss (PMID: 2200955, PMID: 23874507, PMID: 36205042). However, with a few notable exceptions, the increased frequencies of aneuploidy observed in cells of higher ploidy are vulnerable caveats noted in the introduction of our manuscript.

We have added a discussion of this to the new supplemental document called 'Extended Discussion'.

4. The authors use the term "uniparental disomy" (UPD) to refer to cells that are euploid diploids but have two identical copies of a given chromosome due to chromosome loss and subsequent reversion. I think it's good to link yeast aneuploidy data with the UPD concept to highlight the health relevance and compare across species,

but I'm a bit concerned about applying the term equivalently to these systems. In obligate sexuals like humans, we don't normally expect cells with two chromosome copies from the same parent, and UPD may be a good term for the exceptions. But in asexual populations of diploid yeast, both homologs always come from the same parental cell, though they are usually distinct copies. I wonder if the emerging literature could be clearer if we started calling these events chromosome-wide LOH or uni-homolog disomy or chromosome loss revertants or something of that nature, since asexual yeast cells only have one parent.

We recognize that UPD, as defined in the context of sexual reproduction, is not strictly translatable to the genetics of an asexual organism. Nevertheless, the term has clear precedent in the yeast literature, where it has been used recurrently to denote single, whole-chromosome homozygosis (PMID: 22665764; PMID: 18832360; PMID: 31572430). Given this established usage, we believe that adopting a new term would introduce more confusion than it resolves, **and have therefore opted to explicitly introduce UPD and whole-chromosome homozygosis as equivalent terms and then retain the use of UPD throughout the manuscript for consistency with the cited literature.**

5. Elevated LOH distal to the rDNA locus on Chr12 has been seen repeatedly, e.g., PMID: 37847861, PMID: 28369610, PMID: 29799840, PMID: 31371407, PMID: 32727920; it would be good to comment on the relationship between the current results and these previous observations—I'm not sure if the authors would say the elevated LOH in this region could be partly attributable to the chromosome attachment models they discuss, or if other mechanisms are more likely, like standard break induced replication. I might also note that increased UPD with respect to Chr12 (or even just the rDNA region) could help maintain similar rDNA copy number across the homologs.

Good point, **we have included more context—described and cited—regarding the known hyperrecombinant nature of the rDNA in the revised text**.

6. I came across a likely case of "uniparental disomy" in the following study, where the evidence is a chromosome-wide LOH in a "hybrid" strain that started mutation accumulation with high heterozygosity. PMID: 32727920.

Thank you for identifying this unacknowledged citation, **we have referenced it in our discussion of reversion in the revised text.**

7. I gather that the relatively high degree of initial genome wide heterozygosity in the diploids studied here is intentional, making it easier to see LOH and karyotype change, but if this strategy was stated explicitly, I missed it.

This is exactly our rationale, but it was not explicitly stated in the text. **We have added it to the revised version.**

8. Could mutations at the violacein insertion site conceivably affect colony color?

Mutations in the *VIO* cassette can alter pigment production—both in intensity and actual color (PMID 19962399, PMID: 25956652). Moreover, although not mutations per se, the pigment intensity displayed by the parental strains varied by chromosome, suggesting that the qualities of the endogenous sequence flanking the insertion site did impact cassette expression. This is a major reason we performed WGS to confirm the predicted karyotypes of light and dark CANR clones derived from all parental strains.

9. Line 308. A pretty strong fitness cost of trisomy was also observed in PMID: 29760081.

Thank you for noting this. We cited it several sentences after our discussion of trisome stability, but neglected to include it in the references to trisome fitness effects. **We've corrected this oversight in the revised text.**

10. Line 380. "…since these would likely retain at least one CAN1 cassette and fail to form colonies on canavanine-containing media." Sounds backwards, is this an error?

It is not. If cells retain either of the two CAN1 cassettes, they will remain sensitized to the toxic effects of canavanine.

11. In Table S1. LRH316 is derived from LRH307 and LRH303, but I don't see the latter strain listed elsewhere. Maybe it's meant to be LRH330? LRH556 also seems to appear just once.

Thank you for catching this typo. **We've corrected it in the revised supplemental table.**

Reviewer #2:

1. In the abstract and main text, the phrase "alternative mechanism in which nondisjunction and reversion are directly coupled" is somewhat confusing. It may be clearer to describe this instead as a single composite mutational process that produces disomic outcomes without a stable aneuploid intermediate.

We have rephrased these statements in the revised text accordingly.

2. In Fig. S2, the authors test for a correlation between μA and total chromosome size and find none. Given the mechanistic model proposed later in the manuscript, it may be more informative to assess whether μA correlates with the length of the longest chromosome arm rather than total chromosome size per se. If bridge formation, delayed arm resolution, or linkage-mediated breakage contribute to the observed chromosome-specific instability, arm length may be a more relevant structural parameter. This analysis seems particularly pertinent, as the manuscript ultimately argues that, for some chromosomes, instability reflects linkage-mediated missegregation and breakage rather than canonical nondisjunction alone. In this context, the length of the longest arm may be a better predictor of the likelihood that chromatin becomes trapped in the bud neck during mitotic exit.

Cool insight, thank you. We performed the suggested correlative analysis and found that there is no significant relationship between μ^A and chromosome arm length (R^2=0.295, p-value=0.128). In the context of our working model, such a correlation would only be expected if the probability of linkage formation/persistence is equally proportional across all loci and genomic regions. Other data presented in Figures 5 and 6 of the manuscript indicate that this is not the case; rather, specific loci or chromosomal features have a higher probability of forming linkages than others. **This correlative analysis, has been added to the text and to Supplemental Figure 3.**

3. Figure 3B-C reveals substantial chromosome-specific variation in μR, including cases where μR exceeds μA, suggesting that reversion dynamics are heterogeneous. In the Discussion, this complexity is somewhat condensed into a general statement that canonical reversion is rare and largely negligible. While this is broadly supported, the manuscript would benefit from a clearer acknowledgment of the chromosome-specific differences and the contexts in which reversion may be more significant.

We have updated the discussion of this subject in the revised text accordingly.

4. The interpretation that bipolar segregation is required for recombinant formation is somewhat unclear (lines 396–413). Do the authors propose that chromosome breakage arises from spindle-dependent tension during anaphase, from cytokinesis-associated forces during mitotic exit, or from another mechanism? One possibility not made explicit in the text, but consistent with chromatin bridge literature, is that breakage occurs when DNA persists within the bud neck during cytokinesis, independent of whether this positioning results from direct spindle attachment or indirect linkage-mediated displacement. The data presented (e.g. chromatin bridges and bud neck-associated DNA) appear equally consistent with a model in which spatial positioning at cytokinesis, rather than bipolar spindle forces per se, is the primary determinant of chromosome breakage. This interpretation is supported by prior work on dicentric chromosomes (PMID: 25644606, cited by the authors) and - perhaps even more relevant - on chromosomes with intermolecular linkages (PMID: 24286828, 27111841). This point would benefit from further clarification and discussion.

We have clarified this key facet of our model in the revised text. The striking similarity in pericentromeric breakpoint signatures displayed by dicentric molecules and the recombinants in this study is the primary reason we propose that bipolar segregation is a requirement. In the case of dicentric molecules, bipolar segregation of the two centromeres to opposite poles is a requisite for their breakage, which occurs after cytokinesis by the deposition of cell wall materials during septum formation. Here, bipolar attachment appears necessary to establish the spatial positioning of the dicentric, but not for facilitating the breakage event directly—which occurs after the spindle has disassembled and cells have exited mitosis. Current models explaining the pericentromeric-breakage hotspots of dicentrics suggest that the intrinsic architecture of mitotic chromosomes and the spindle increases the probability that—regardless of where the fusion/linkage site occurs along the arm of a chromosome—a centromere will be nearer to the budneck when cytokinesis occurs (PMID: 25644606). This model is supported by our microscopy experiments (Figure, 7 and 8).

5. The proposed mechanism of linkage-mediated chromosome breakage is highly reminiscent of prior work showing that late-replicating regions (e.g. subtelomeres or rDNA) can remain unresolved into anaphase and incur damage upon cytokinesis in otherwise unperturbed cells. Notably, cytokinesis induces subtelomeric mutations in these cases (PMID: 32385287). This raises the possibility that the phenomena described here represent a chromosome-scale manifestation of previously described locus-specific replication/segregation defects. The manuscript would benefit from a more explicit integration of this literature, and clarification of what aspects of the mechanism are genuinely novel versus extensions of these established observations. In this context, the novelty appears to lie less in identifying cytokinesis-associated damage per se, and more in extending its consequences to chromosome-scale aneuploidy dynamics.

An expanded discussion of the literature on linkage formation and linkage-mediated mutational processes is absolutely warranted. **We have extended this discussion in the revised text accordingly.**

Reviewer #3:

MAJOR CONCERNS:

1. The fflucsim Structural Assumptions: The authors present a thorough exploration of parameter space within fflucsim, pushing μR to values approaching 1 and demonstrating that even this extreme cannot recapitulate Chr12 dynamics. This is a genuinely strong result within the model's framework. However, a structural assumption of the simulator warrants explicit acknowledgment that is currently absent from the paper. To my understand the fflucsim models population expansion as a well-mixed system, yet the monosome replating assay — from which the revertant frequency distributions being modeled are derived — is performed on solid agar medium where spatially clustered progeny from a single early reversion event remain physically grouped. For chromosomes with extreme fitness costs such as Chr12 ($\omega \approx 0.25$), a single reversion event occurring within the first few divisions of a slow-growing monosomic colony could generate a spatially expanding revertant subclone that dominates the countable colony area by day 2 through fitness-driven spatial expansion rather

than through genuinely elevated μR. No combination of μR and ω within a well-mixed population model can reproduce this dynamic because the model is structurally incapable of representing spatial heterogeneity, regardless of what parameter values are explored. The directly testing this concern is non-trivial given the constraints of the violacein-based detection system. Performing the assay in liquid culture would eliminate spatial structure but destroy the pigment-based readout, and a time course approach on solid medium might be confounded by the inherent lag between reversion event occurrence and sufficient violacein accumulation for visual detection. The paper's existing data provides partial mitigation — the liquid-grown fluctuation test populations and the WGS characterization of individual clones are both independent of solid medium geometry and broadly consistent with the replating assay results. Nevertheless, the well-mixed assumption represents an unacknowledged limitation of the analytical framework that should be explicitly discussed. The authors should address this limitation in the Discussion and note that fully resolving it would require development of a detection system that does not depend on pigment accumulation on solid medium. Until such a system is developed, the conclusion that Chr12 dynamics are incompatible with any canonical parameter combination must be framed with the caveat that the assay geometry itself introduces spatial heterogeneity that the model cannot capture.

All of our fluctuation tests and monosome replating assays constitute well-mixed systems. In the monosome replating tests specifically, we picked single monosomic colonies from canavanine plates with sterile toothpicks and resuspended them vigorously in 200uL of water. A dilution of this well-mixed population was then replated onto complete media to enable us to quantify the frequency of revertant cells that had existed in the original monosomic colony. Likewise, our canavanine fluctuation tests began with liquid cultures or resuspended colonies (again in 200uL of water) that were pregrown in permissive conditions and plated onto canavanine media. In all cases, spatial heterogeneity is eliminated as a confounding factor in the quantification of mutant cell counts. Thus, our empirical estimates of nondisjunction rates are comparable to those derived from the fflucsim simulations. **We have clarified these approaches more clearly in the text.**

2. Molecular identity of the linkages (Chr4 and Chr12): No experiment demonstrates that disrupting linkage formation specifically reduces recombinant clone frequency for Chr4 and Chr12 while leaving stable chromosomes unaffected. The molecular species constituting the linkages — topological catenation, cohesin entrapment, unresolved recombination intermediates — is never identified, which limits the mechanistic conclusions and prevents connecting the proposed mechanism to specific molecular perturbations. Deleting FOB1 in the CAN1-VIO Chr12 strain and repeat the monosome replating assay and WGS characterization would directly tests whether rDNA recombination intermediates are the linkage species driving Chr12 non-canonical reversion. Fob1 deletion specifically reduces rDNA recombination without affecting transcription or copy number. The prediction is falsifiable: fob1Δ should reduce recombinant clone frequency for Chr12 — particularly the rDNA breakpoint class in Figure S7 — without affecting Chr1 or Chr2 reversion dynamics. This would be a more direct test for one of the main hypothesis generated from this work data. Another direct assay would be to engineer a truncated rDNA array of defined size inserted at a defined locus on Chr3, a chromosome showing clean canonical monosomic behavior. Introduce this modified Chr3 into the CAN1-VIO system and measure μR, recombinant clone frequency, and breakpoint patterns by WGS, comparing to unmodified Chr3. This experiment directly tests sufficiency — whether rDNA presence alone is sufficient to convert a stable chromosome into a non-canonical reverter — completely decoupled from Chr12 identity, centromere sequence, and gene content. A positive result would also have significant broader implications for understanding instability of any chromosome bearing large repetitive arrays, including human acrocentric chromosomes.

We agree, the molecular basis of the linkages we have observed is not mechanistically elucidated in this current manuscript. The goal of the current study was to explore the population dynamics of aneuploidy. In so doing, we have observed that nondisjunction mediated by intermolecular linkages contributes to these

dynamics. Given the density and length of the current manuscript, we feel that including a third major section—the molecular dissection of linkage-mediated nondisjunction—would be both excessive and outside the scope of the study. Indeed, the suggested experiments are directly in line with our ongoing exploration of linkage-mediated nondisjunction. Yet, a thorough investigation of the basis of linkages will constitute a substantial body of additional work, one that we feel would be most impactful as a stand-alone study unto itself.

3. The GAL1p-CEN Checkpoint Issue: The authors explicitly acknowledge the artificial biology: "Although the URA3-GAL1p-CEN system generates a mitotic scenario that would rarely occur in wild-type cells—because an unattached chromatid pair typically activates a mitotic checkpoint-mediated cell cycle arrest—it constituted a valuable tool to further probe the validity of the intermolecular linkage model." This acknowledgment is appropriate but the checkpoint escape mechanism is never characterized. In Figure 7, 28.3% of cells show normal disjunction, 55% show induced nondisjunction, and approximately 17% show alternative segregation — meaning a substantial fraction of cells with inactivated centromeres escape checkpoint arrest and complete division. Whether checkpoint escape rates vary across chromosomes, which would bias which chromosomes produce recombinant FOA resistant clones, is never addressed. Additionally the uracil starvation pregrowth required by the experimental design could independently influence chromosome segregation fidelity through replication stress, a confound that is not discussed. An assay to measure checkpoint activation duration and/or escape frequency per chromosome would be important to strength further statements. If checkpoint escape rates differ significantly across chromosomes — particularly if Chr12 and Chr4 show higher escape rates than Chr1 and Chr3 — this would indicate that differential checkpoint biology contributes to the observed differences in recombinant clone recovery, requiring reinterpretation of the GAL1p-CEN results.

The mechanism of checkpoint bypass that occurs in the *GAL1p-CEN* system is discussed in several studies cited in the manuscript (PMID: 15846338, PMID: 3302676, PMID: 25644606, PMID: 18832360). Cells in which the conditional centromere is inactivated by transcription fail to mount a spindle assembly checkpoint arrest in the presence of the unattached chromosome because a functional kinetochore is not formed upon the transcribed centromere sequence. Without a functional kinetochore to convert monomeric checkpoint factors into mitotic checkpoint complexes, the cell will not sense the unattached chromosome and will proceed through mitosis without delay. Technically speaking, this is not an escape so much as a detection failure. **We have provided revisions to the text clarifying the details of this system more explicitly.**

Whether the efficiency of induced centromere inactivation varies across chromosomes is intriguing, and we can speculate as to how differences in pericentromeric chromatin structure could mediate such variation. Differences in conditional centromere inactivation efficiency across chromosomes would alter the frequency at which FOA^R colonies are recovered, but would not be expected to influence the probability of linkage formation between chromosomes or the recovery of recombinant clones. Although all strains subjected to these experiments were uracil prototrophs, we cannot dismiss the possibility that pregrowth in media lacking uracil could increase basal levels of replication stress. We would not expect this to drive any chromosome-specific biases. Since we do not quantify or compare rates of FOA^R recovery across chromosomes or between experiments, the impacts of differential centromere inactivation or increased replication stress do not confound the conclusions we deduce in the current study. For the modest claims we make regarding the Chr4 and Chr12 bias in linkage-mediated nondisjunction, we provide additional data from orthologous approaches that do not rely on centromere inactivation (Fig. 5A).

4. Fitness Measurement Discordance Between Assays: Figure 2A shows systematic differences between induced and preselected ω values. The exclusion criterion for the preselected assay is stated: "this approach was restricted to monosomes that consistently exhibited undetectable revertant frequencies." This justification is reasonable — preselected monosomes cannot be measured for fast-reverting chromosomes because the

populations are already contaminated with revertants by the time of measurement.However, the paper uses induced ω values for these fast-reverting chromosomes in the fflucsim modeling without validating that the induced assay accurately captures true monosomic fitness for chromosomes like Chr12 where alternative segregation events occur at high rates during the induction period. Cells surviving galactose induction and 5-FOA selection for fast-reverting chromosomes may not be representative monosomes but rather cells that underwent alternative segregation events, potentially reporting fitness values that reflect a mixed population rather than pure monosomes.

Indeed, for some of the chromosomes subjected to both induced and preselected fitness assays, the estimated ω values differ, though most are statistically insignificant. It is for this very reason that we performed replicate fflucsim simulations across a broad parameter range of ω. **We noted the potential errors in estimating ω in the original text, but have emphasized it further in the revised version.** The key findings from these broad-range fflucsim studies are: 1) that no ω value across the observed range captured by our empirical studies can explain the reversion dynamics of Chr4, Chr10, and Chr13, and 2) that no ω value *whatsoever* can explain the observed reversion dynamics of Chr12. We also acknowledge the confounding effects of mixed populations on estimating ω. In fact, we accounted for this in our analysis by limiting the timeframe of growth. **We have added a new supplemental figure (Supplemental figure 2) and related discussion to the revised text.**

5. Replating Assay Temporal Asymmetry: The Methods state: "After 48hrs, dark revertant colonies were quantified; after 96hrs, light monosomic colonies were quantified." The fitness correction applied through mlemur is described but the specific appropriateness of mlemur's correction for solid-medium colony counting — as opposed to the liquid culture fluctuation tests for which it was designed — is never discussed. Looking at Supplemental Table 5, individual replicate revertant frequencies for Chr4 range from 0% to 97%, and for Chr13 show enormous variance. This variance itself suggests that the fitness correction may be insufficient for the solid-medium geometry, where spatial structure creates non-linear expansion dynamics that the population-level correction cannot capture. The temporal asymmetry and fitness correction appropriateness for solid-medium colony counting are not addressed, and the high variance in replicate revertant frequencies for intermediate chromosomes suggests the correction may be insufficient. The Methods section should explicitly acknowledge that mlemur's fitness correction assumes well-mixed population dynamics and discuss whether this assumption holds for the solid-medium replating assay geometry. The Discussion should acknowledge that high variance in revertant frequencies across replicates for chromosomes like Chr4 and Chr13 could reflect either genuine Luria-Delbrück jackpot dynamics or spatial expansion artifacts, and that these explanations are not fully distinguishable with the current experimental system.

mlemur is not designed only for assays in which mutant cell counts are inferred directly from liquid cultures. It is intended to facilitate an improved analysis of typical fluctuation test data (PMID: 37104996) derived from standard experimental approaches: replicate populations of cells are first pregrown in a given condition and then plated onto selective media to quantify mutant cell counts. Because we performed our assays in just this manner, we see no conflict between our experimental approach and the analytical assumptions posed by mlemur or any other MLE-MSS analysis.

As mentioned above, our methodologies eliminate spatial structure within a colony as a confounding factor in estimating aneuploid or revertant frequencies within the population. The cross-replicate variance in revertant frequencies for chromosomes like Chr4 and Chr13 reflects two expected features of mutant frequency dynamics: 1) the differential fitness between monosomes and revertant disomes, and 2) variance in the time at which point the reversion event occurred in the growth of the monosomic colony. Because we fully mix each population—whether derived from a liquid microculture or a solid-agar grown colony—prior to replating, we have explicitly eliminated spatial expansion artifacts as a confounding effect and can account for both fitness and typical Luria-Delbrück jackpot events in our estimates of mutation rate.

6. WGS Sample Sizes: Supplemental Table 2 reveals that for several chromosomes, as few as 5 light CAN^R clones were sequenced. The core Chr4 finding — 8/10 dark CANr clones showing pericentromeric breakpoints — is presented without confidence intervals or statistical comparison to other chromosomes. The paper never calculates whether this sample size provides sufficient power to detect the claimed frequency difference from other chromosomes or from what would be expected by chance. The authors do provide a larger dataset for Chr12 specifically through the single-cassette strain experiment in Figure 5F-H, sequencing 45 dark CAN^R clones, which is more reassuring for that specific claim. But the generalization to other chromosomes remains statistically underpowered.

We agree, the sequencing data are underpowered for drawing any strong conclusions about the likelihood of specific chromosomes to experience linkage-mediated nondisjunction. It is for this reason that we do not make quantitative or broad conclusions about the absolute or relative incidence of this type of event in the manuscript. Rather, we limit our interpretations to observational evidence that centromere-proximal breakpoints occur for numerous chromosomes. **Where possible, we have softened the language of these conclusions in the revised text.** The WGS studies were performed to validate the efficacy of our selectable system. Although some genotype-phenotypes are undersampled (due to their extreme rarity in incidence), the collective dataset demonstrates that the CAN-VIO system accurately discerns between monosomic and disomic karyotypes. Only in the experiment described in Fig. 5F-H do we make quantitative claims about relative mutation incidence and spectra. In this case, we performed sufficient sampling to substantiate our conclusions.

7. The rDNA Linkage Hypothesis: The rDNA hypothesis is introduced as an observation — 22% of Chr12 FOAR clones show rDNA breakpoints — and elevated to a general mechanism in the Discussion. The authors acknowledge this is a hypothesis: "Our work prompts the hypothesis that even in unperturbed wild type cells, persistent linkages existing within the ~1Mb rDNA array facilitate high-frequency chromosome nondisjunction events." The fact that 78% of Chr12 FOAR clones did not show rDNA breakpoints is never explicitly addressed as a challenge to the rDNA linkage model being general for Chr12. The authors propose that rDNA-mediated events resolving through repetitive region breakage would appear as typical UPDs indistinguishable from canonical reversion — which is a reasonable explanation but is not tested. The proximity of the GAL1p-CEN12 cassette to the rDNA in the strain construction raises the possibility that rDNA breakpoints are an assay architecture artifact, which is also never formally excluded.

As the reviewer points out, we propose rDNA linkage-mediated nondisjunction only as a hypothesis to explain the following observations: 1) the notable incidence of rDNA breakpoints in Chr12 FOA^R clones, 2) the notable coincidence of rDNA breakpoints in FOA^R clones derived from *other* strains, and 3) the high apparent reversion frequency Chr12. Due to the scope and size of this manuscript, we do not test this hypothesis further, but provide a speculative presentation of it in the discussion, which we—and the other reviewers too, apparently—feel is both necessary and warranted.

CEN12 is ~300kb away from the proximal boundary of the rDNA locus. It is also physically separated in the nucleus, with the rDNA comprising the distinct nucleolar compartment. To our knowledge, there is no described mechanism in which transcriptional disruption of such a distant locus (*CEN12)* would promote linkage formation of another that is so far away. This is not to say that such a mechanism does not exist, but as it stands, we have no reason to suspect that our observations reflect a limitation of our assay, particularly in light of the large body of work substantiating the rDNA as a hyperrecombinant and late-segregating locus. **We have added additional citations and and discussion of this literature in the revised text.**

8- Chr6 and Chr7 Exclusion Requires Explicit Justification: The paper claims to provide a systematic analysis of aneuploidy dynamics across all 16 yeast chromosomes, yet Chr6 and Chr7 are effectively excluded from the quantitative rate analysis without explicit criteria stated in the main text. The authors correctly identify that these chromosomes induce chromosomal instability through ACT1/TUB2 dosage imbalance, which violates the stable mutation rate assumption of fluctuation analysis. However, this exclusion justification appears only implicitly through the mechanistic discussion of CIN, never as an explicit statement of scope limitation. More importantly, the Methods note that VIO cassette integration failed repeatedly for several strains including those used for Chr6 and Chr7 — a significant technical limitation mentioned only in a footnote without connection to the main results narrative. The paper should explicitly state in the Results which chromosomes are excluded from which analyses and why, rather than allowing readers to piece this together from scattered statements across Methods and supplementary materials. I also noted that whether the CIN phenotype of Chr6 and Chr7 is mechanistically related to the non-canonical reversion observed in Chr4 and Chr12 is an interesting question the paper raises but does not address. Given the authors' proposed intermolecular linkage mechanism, it is worth considering whether the elevated nondisjunction rates induced by Chr6 aneuploidy could themselves generate linkage-mediated events on other chromosomes — a possibility that would have implications for interpreting the unselected aneuploidy burden observed in Figure S1. Without the need to perform a new experiment the author could perform a lower-resolution analysis that might be achievable with the existing deposited sequencing data to specifically, test whether the midpoints of unselected LOH events in Chr6 and Chr7 light CANr clones show statistically significant enrichment near centromeres relative to random chromosomal positions. This analysis would either provide preliminary support for linkage-mediated events in a CIN context or appropriately conclude that the existing data lacks sufficient resolution to address the question. Either outcome is informative and should be reported as a supplementary analysis.
Additionally, the Discussion should include a brief paragraph acknowledging that whether Chr6-induced CIN generates linkage-mediated events as a mechanistic consequence of spindle disruption remains an open question, identifying it as an important direction for future work. This would substantially improve the paper's mechanistic coherence and honestly situate the Chr6/Chr7 observations within the broader intermolecular linkage framework the paper proposes.

We have added an explicit discussion of how the CIN phenotypes displayed by Chr6 and Chr7 clones precluded an accurate deduction of fitness and reversion in the results section of the revised text. The inability to integrate the VIO cassette into the Chr6 and Chr7 strains is indeed an inconsistency in strain design, but it did not prove to be a technical limitation; every 48hr-CAN^R clone was subjected to WGS validation and every one harbored the expected disomic karyotype. For this reason, we do not think a discussion of this complication in the results section provides meaningful context for the conclusions we present on the analysis of Chr6 and Chr7 CAN^R clones. We have made no effort to conceal this feature of the Chr6 and Chr7 strains and describe it with transparency in both the methods and strain table.

To the new 'Extended Discussion' document, we have added the following discussion of CIN as it relates to linkage-mediated nondisjunction: CIN phenotypes can manifest broad mutational spectra. The CIN displayed in the sequenced clones of Chr6 and Chr7 is generally attributed to defects in microtubule assembly and stability, and is thought to manifest in increased rates of simple nondisjunction events. However, we have also considered the possibility that it might also result in an increase in linkage-mediated nondisjunction typified by centromere-proximal breakpoints. As a preliminary test of this hypothesis, we assessed the spectra of unselected aneuploidies—simple or recombinant—harbored by the light CAN^R clones of Chr6 and Chr7. All of the unselected aneuploidies detected were simple; we detected no examples of recombinant aneuploidy. Only 3 unselected recombination tracts were detected amongst the clones: 2/3 events were mosaic terminal LOH tracts with the breakpoint arising in the ribosomal DNA; the other event constituted a terminal LOH tract on the right arm of Chr8 with the breakpoint near the subtelomere. The co-occurrence of

rDNA-originating LOH could suggest that the CIN phenotype displayed by these clones increases linkage-mediated nondisjunction. However, this is a speculation that requires further study.

MINOR CONCERNS

1.Yeast-to-Human Extrapolation: The Abstract states the work "expands our perspective of the diverse, and chromosome-specific mutational mechanisms shaping genome architecture" in a context framed around human disease. In the discussion section we can see the following statement "Discerning the underlying basis for this variance warrants further study, as doing so is certain to clarify central principles of genome stability and refine current paradigms of aneuploidy-driven disease risk". But I believe it is important to address in the paper that there are fundamental difference between S. cerevisiae point centromeres (~125bp, single CENP-A nucleosome) and human regional centromeres (megabases, complex chromatin) acknowledged as a limitation on extrapolation. The intermolecular linkage mechanism proposed here specifically involves rDNA on Chr12 and centromere-proximal recombination — both features that are organized entirely differently in humans. The word "certain" in "certain to clarify" is an overstatement that no data in the paper supports.

Where possible, we have further tempered statements implying that the mechanisms of chromosome loss observed in humans are the same. We have also added a section in the 'Extended Discussion' that compares some particularly distinguishing differences between yeast and human mitoses.

2. Genetic Background Effects
Supplemental Table 1 shows Chr5 diploid strain is constructed as "(LRH279 x LRH202)" where LRH279 is in JAY291 background while most other strains are S288c. Several strains also show mixed parentage.
The Methods state only: "Strain construction was performed using standard transformation, crossing, and sporulation procedures" with no discussion of background standardization.
What is missing: The paper never discusses whether the JAY291 vs S288c background difference for Chr5 could contribute to its apparently distinct behavior in the fitness and rate analyses. JAY291 is a Brazilian bioethanol strain with substantially different genome content, ploidy history, and stress response compared to S288c. Given that Chr5 shows one of the higher μA values (Figure 3A) and relatively normal reversion dynamics, the possibility that background effects contribute to this pattern is never considered.

All strains used in the rate estimation section and recombinant disome experiments described in this manuscript are constructed in the same hybrid background (S288c x JAY291). The only difference between the Chr5 strain and the others is that the *CAN1*-marked chromosome is the JAY291 homolog, and the VIO marked chromosome is the corresponding S288c homolog. Thus, rate comparisons amongst all chromosome strains tested are standardized by a common heterozygous genetic background. **Upon rechecking the strain information, we identified two typos that we have remedied.**

3. The "Unified Model" Framing: The title claims "A unified model of aneuploid karyotype dynamics" and the author states: "Collectively, this work establishes a unified model of aneuploid population genetics"
But the author also acknowledges: "The genetic systems used in this study allowed detection of but a subset of outcomes predicted by our models of linkage-mediated missegregation, suggesting that we have substantially underestimated the true incidence of this mutational mechanism" And: "Discerning the underlying basis for this variance warrants further study" regarding why some chromosomes are subject to linkage-mediated instability more than others. A truly unified model would provide quantitative criteria to predict which chromosomes undergo canonical versus non-canonical reversion. Instead, the classification is entirely post-hoc — chromosomes are labeled non-canonical because their empirical data deviates from model predictions, not because any feature of the chromosome predicts non-canonical behavior prospectively. The Discussion

speculates about centromere sequence variation, nuclear organization, and rDNA content as potential predictors, but none of these are tested. The model describes two classes of chromosome behavior but cannot predict class membership from first principles, which substantially limits its utility as a "unified" framework. So please re-consider the use of "unified framework".

Where possible, we have softened the use of ‘unified’ throughout the text. Perhaps the most pronounced tempering of this language is in the title, which we have changed to ‘Towards a unified model of aneuploid karyotype dynamics’.
