# Supplementary material for: Towards a unified model of aneuploid karyotype dynamics
Source: PLoS Genet. 2026 Jun 18;22(6):e1012210. doi: 10.1371/journal.pgen.1012210 (PMC13318051; doi:10.1371/journal.pgen.1012210)
Supplement: Peer review history

# Peer review: Decision Letter 0

**DOI:** 10.1371/journal.pgen.1012210.r001

**Authors:** Kent W. Hunter, Soni Lacefield

30 Apr 2026

PGENETICS-D-26-00199

A unified model of aneuploid karyotype dynamics

PLOS Genetics

Dear Dr. Heasley,

Thank you for submitting your manuscript to PLOS Genetics. After careful consideration, we feel that it has merit but does not fully meet PLOS Genetics's publication criteria as it currently stands. Therefore, we invite you to submit a revised version of the manuscript that addresses the points raised during the review process.

Please submit your revised manuscript by Jun 28 2026 11:59PM. If you will need more time than this to complete your revisions, please reply to this message or contact the journal office at plosgenetics@plos.org. When you're ready to submit your revision, log on to https://www.editorialmanager.com/pgenetics/ and select the 'Submissions Needing Revision' folder to locate your manuscript file.

Please include the following items when submitting your revised manuscript:

* A letter that responds to each point raised by the editor and reviewer(s). You should upload this letter as a separate file labeled 'Response to Reviewers'. This file does not need to include responses to any formatting updates and technical items listed in the 'Journal Requirements' section below.

* A marked-up copy of your manuscript that highlights changes made to the original version. You should upload this as a separate file labeled 'Revised Manuscript with Track Changes'.

* An unmarked version of your revised paper without tracked changes. You should upload this as a separate file labeled 'Manuscript'.

If you would like to make changes to your financial disclosure, competing interests statement, or data availability statement, please make these updates within the submission form at the time of resubmission. Guidelines for resubmitting your figure files are available below the reviewer comments at the end of this letter.

We look forward to receiving your revised manuscript.

Kind regards,

Soni Lacefield, PhD

Academic Editor

PLOS Genetics

Kent Hunter

Section Editor

PLOS Genetics

Aimée Dudley

Editor-in-Chief

PLOS Genetics

Anne Goriely

Editor-in-Chief

PLOS Genetics

**Additional Editor Comments:**

Overall, the reviewers agree that this is an interesting study that will contribute to our understanding of aneuploidy. However, the reviewers have made several comments to improve the manuscript with some new experiments and additional discussion points, clarifications, and reference to other studies through writing and analysis. Please try to address these concerns where possible.

**Journal Requirements:**

1) We ask that a manuscript source file is provided at Revision. Please upload your manuscript file as a .doc, .docx, .rtf or .tex. If you are providing a .tex file, please upload it under the item type u2018LaTeX Source Fileu2019 and leave your .pdf version as the item type u2018Manuscriptu2019. 2) Please provide an Author Summary. This should appear in your manuscript between the Abstract (if applicable) and the Introduction, and should be 150-200 words long. The aim should be to make your findings accessible to a wide audience that includes both scientists and non-scientists. Sample summaries can be found on our website under Submission Guidelines:https://journals.plos.org/plosgenetics/s/submission-guidelines#loc-parts-of-a-submission 3) Please upload all main figures as separate Figure files in .tif or .eps format. For more information about how to convert and format your figure files please see our guidelines: https://journals.plos.org/plosgenetics/s/figures 4) We have noticed that you have uploaded Supporting Information files, but you have not included a complete list of legends. Please add a full list of legends for your Supporting Information files (Supplemental movies) after the references list. 5) We notice that your supplementary Figures, and Tables are included in the manuscript file. Please remove them and upload them with the file type 'Supporting Information'. Please ensure that each Supporting Information file has a legend listed in the manuscript after the references list. 6) Your current Financial Disclosure states, "The author(s) received no specific funding for this work.".

However, your funding information on the submission form indicates receiving funds.Please ensure that the funders and grant numbers match between the Financial Disclosure field and the Funding Information tab in your submission form. Note that the funders must be provided in the same order in both places as well. Please amend your detailed Financial Disclosure statement. This is published with the article. It must therefore be completed in full sentences and contain the exact wording you wish to be published.1) Please clarify all sources of financial support for your study. List the grants, grant numbers, and organizations that funded your study, including funding received from your institution. Please note that suppliers of material support, including research materials, should be recognized in the Acknowledgements section rather than in the Financial Disclosure2) State the initials, alongside each funding source, of each author to receive each grant. For example: "This work was supported by the National Institutes of Health (####### to AM; ###### to CJ) and the National Science Foundation (###### to AM)."3) State what role the funders took in the study. If the funders had no role in your study, please state: "The funders had no role in study design, data collection and analysis, decision to publish, or preparation of the manuscript."4) If any authors received a salary from any of your funders, please state which authors and which funders.

7) Some material included in your submission may be copyrighted. According to PLOS’s copyright policy, authors who use figures or other material (e.g., graphics, clipart, maps) from another author or copyright holder must demonstrate or obtain permission to publish this material under the Creative Commons Attribution 4.0 International (CC BY 4.0) License used by PLOS journals. Please closely review the details of PLOS’s copyright requirements here: PLOS Licenses and Copyright. If you need to request permissions from a copyright holder, you may use PLOS's Copyright Content Permission form.

Please respond directly to this email and provide any known details concerning your material's license terms and permissions required for reuse, even if you have not yet obtained copyright permissions or are unsure of your material's copyright compatibility. Once you have responded and addressed all other outstanding technical requirements, you may resubmit your manuscript within Editorial Manager.

Potential Copyright Issues:

Figures 1B, and 1E. Please confirm (a) that you are the photographer; or (b) provide written permission from the photographer to publish the photo(s) under our CC BY 4.0 license..

Note: If the reviewer comments include a recommendation to cite specific previously published works, please review and evaluate these publications to determine whether they are relevant and should be cited. There is no requirement to cite these works unless the editor has indicated otherwise.

**Reviewers' comments:**

Reviewer's Responses to Questions

Reviewer #1: This manuscript describes a series of experiments that both confirm some suspected patterns of karyotype evolution in yeast and reveal some new patterns using novel methods.

1. In the introduction, it might be helpful to clarify that many of the studies cited used empirical approaches designed to minimize the influence of selection on the detection of aneuploidy and other mutants (as they should). For instance, in mutation accumulation (MA) experiments, it’s certainly true that the possibility of aneuploidy reversion is generally not accounted for, and that differences in fitness could potentially confound rate estimates; however, even with these caveats, MA or fluctuation test style experiments will be a much more useful approaches than other types of experimental evolution where karyotypes evolve under selection, assuming our goal is to understand intrinsic mutation properties. The authors know all this, but in my experience many geneticists are not used to thinking about this distinction, and may assume that the karyotypic changes in question are under strong selection in these experiments.

2. It is known that classical nondisjunction, where a chromosome gain in one daughter cell is coupled with a chromosome loss in the other daughter cell, is not the only means through which aneuploidy might arise. In particular, chromosome loss could occur through “anaphase lag”, where there is a loss without a corresponding gain. If this is happening in yeast, we might expect the rate of mutation to monosomy (which can occur through nondisjunction or chromosome lag) to exceed the rate of reversion, which can only occur through nondisjunction, all else being equal. I’d ask the authors to consider how the anaphase lag model might apply to their observations.

3. As we move towards unbiased quantification of karyotype mutations, I wonder if the authors have considered whether the probability of nondisjunction in a given cell division ought to scale with the current number of chromosome copies. For example, all else being equal, the probability of segregation error occurring in a tetraploid should be twice that of a diploid, since tetraploids have to manage twice as many chromosomes overall. When thinking about aneuploidy reversion in diploids, this would suggest that reversion from trisomy to euploidy is more likely than reversion from monosomy to euploidy.

4. The authors use the term “uniparental disomy” (UPD) to refer to cells that are euploid diploids but have two identical copies of a given chromosome due to chromosome loss and subsequent reversion. I think it’s good to link yeast aneuploidy data with the UPD concept to highlight the health relevance and compare across species, but I’m a bit concerned about applying the term equivalently to these systems. In obligate sexuals like humans, we don’t normally expect cells with two chromosome copies from the same parent, and UPD may be a good term for the exceptions. But in asexual populations of diploid yeast, both homologs always come from the same parental cell, though they are usually distinct copies. I wonder if the emerging literature could be clearer if we started calling these events chromosome-wide LOH or uni-homolog disomy or chromosome loss revertants or something of that nature, since asexual yeast cells only have one parent.

5. Elevated LOH distal to the rDNA locus on Chr12 has been seen repeatedly, e.g., PMID: 37847861, PMID: 28369610, PMID: 29799840, PMID: 31371407, PMID: 32727920; it would be good to comment on the relationship between the current results and these previous observations––I’m not sure if the authors would say the elevated LOH in this region could be partly attributable to the chromosome attachment models they discuss, or if other mechanisms are more likely, like standard break induced replication. I might also note that increased UPD with respect to Chr12 (or even just the rDNA region) could help maintain similar rDNA copy number across the homologs.

6. I came across a likely case of “uniparental disomy” in the following study, where the evidence is a chromosome-wide LOH in a “hybrid” strain that started mutation accumulation with high heterozygosity. PMID: 32727920.

7. I gather that the relatively high degree of initial genome wide heterozygosity in the diploids studied here is intentional, making it easier to see LOH and karyotype change, but if this strategy was stated explicitly, I missed it.

8. Could mutations at the violacein insertion site conceivably affect colony color?

9. Line 308. A pretty strong fitness cost of trisomy was also observed in PMID: 29760081.

10. Line 380. “…since these would likely retain at least one CAN1 cassette and fail to form colonies on canavanine-containing media.” Sounds backwards, is this an error?

11. In Table S1. LRH316 is derived from LRH307 and LRH303, but I don’t see the latter strain listed elsewhere. Maybe it’s meant to be LRH330? LRH556 also seems to appear just once.

Reviewer #2: When a chromosome is lost (typically through nondisjunction), how stable is that loss in a population, and what determines its persistence? Key factors include the fitness of the resulting aneuploid cells and the likelihood of reversion to a euploid state, often through a second nondisjunction event. These parameters are likely to vary across organisms and cell types, but according to the authors, a comprehensive characterization within a single system has been lacking. This study addresses this question using diploid budding yeast, examining how aneuploidy persistence is shaped by the rates of chromosome loss, the fitness of the resulting cells, and reversion to euploidy. The authors employ a well-designed experimental system to quantify these variables on a per-chromosome basis, with particular emphasis on reversion. The manuscript first establishes a quantitative framework describing aneuploid formation, fitness, and reversion across all chromosomes. It then shows that, for a subset of chromosomes, the observed “reversion” dynamics cannot be explained by canonical sequential nondisjunction. In the second part, the authors propose and support an alternative mechanism (linkage-mediated missegregation followed by chromosome breakage and recombination) that produces disomic outcomes without a stable aneuploid intermediate.

In the first part of the study (Figures 1–3), the authors develop an elegant experimental framework to quantify spontaneous aneuploidy formation, reversion, and fitness. Aneuploidy formation is measured using selection for loss of a CAN1 marker inserted on both arms of each chromosome, followed by whole-genome sequencing (WGS) of selected clones. Reversion frequency is inferred from a shift from heterozygosity to homozygosity at a linked marker encoding a pigment-producing gene (resulting in a dark colony phenotype) followed by WGS. Fitness is assessed using population doubling times in liquid culture. These measurements are then integrated into a computational model. Using this approach, the authors show that the fitness of aneuploid strains is chromosome-dependent, as expected. They further find that, for most chromosomes, reversion rates differ substantially from aneuploidy formation rates, consistent with an unequal likelihood model. A key conclusion from this analysis is that classical two-step reversion via nondisjunction is rare and contributes minimally to population dynamics.

In the second part of the study (Figures 4–8), the authors report that a subset of chromosomes (Chr4, 10, 12, and 13) exhibit unusually high apparent reversion rates that cannot be explained by standard models. They interpret these cases as arising from a non-canonical pathway: intermolecular linkage leads to missegregation, chromosome breakage and recombination, effectively coupling “formation” and “reversion.” In this framework, the data are better explained by a model in which chromosome loss is accompanied by partial gains, suggestive of chromosome breakage events, potentially occurring during late mitosis or cytokinesis in the presence of incompletely segregated chromosomes. To support this model, the authors analyze cells in which nondisjunction is artificially induced and observe features consistent with this mechanism, including characteristic patterns in whole-genome sequencing and direct visualization of chromosome segregation defects by live-cell imaging. Based in part on these time-lapse experiments, they propose that a subset of chromosome-specific aneuploidies arise from “intermolecular linkage-mediated missegregation,” in which unresolved linkages between sister chromatids lead to chromatin bridges that are subsequently damaged during mitosis or cytokinesis, resulting in chromosome fragmentation and rearrangement.

The first part of the paper is clear and well presented, and the conclusions appear well supported by the data. The second part is more challenging to follow but also more conceptually interesting, as it provides, to my knowledge, one of the first quantitative analyses of partial chromosome missegregation in unperturbed yeast populations. Some sections would benefit from improved clarity and more precise definitions. The mechanistic model proposed (linkage-mediated missegregation followed by chromosome breakage and recombination) is well grounded in prior work on anaphase bridges (which is not always cited). While its application to explain the observed reversion dynamics is compelling, the manuscript would benefit from a clearer distinction between what is conceptually new (notably the quantitative integration with population genetics and the chromosome-specific effects) and what is already established in the literature. As written, the manuscript occasionally gives the impression that this represents a previously unrecognized mechanism. However, the underlying processes are well documented; the novelty here lies more in demonstrating their contribution under unperturbed conditions and linking them to apparent reversion rates.

Specific comments

1. In the abstract and main text, the phrase “alternative mechanism in which nondisjunction and reversion are directly coupled” is somewhat confusing. It may be clearer to describe this instead as a single composite mutational process that produces disomic outcomes without a stable aneuploid intermediate.

2. In Fig. S2, the authors test for a correlation between μA and total chromosome size and find none. Given the mechanistic model proposed later in the manuscript, it may be more informative to assess whether μA correlates with the length of the longest chromosome arm rather than total chromosome size per se. If bridge formation, delayed arm resolution, or linkage-mediated breakage contribute to the observed chromosome-specific instability, arm length may be a more relevant structural parameter. This analysis seems particularly pertinent, as the manuscript ultimately argues that, for some chromosomes, instability reflects linkage-mediated missegregation and breakage rather than canonical nondisjunction alone. In this context, the length of the longest arm may be a better predictor of the likelihood that chromatin becomes trapped in the bud neck during mitotic exit.

3. Figure 3B-C reveals substantial chromosome-specific variation in μR, including cases where μR exceeds μA, suggesting that reversion dynamics are heterogeneous. In the Discussion, this complexity is somewhat condensed into a general statement that canonical reversion is rare and largely negligible. While this is broadly supported, the manuscript would benefit from a clearer acknowledgment of the chromosome-specific differences and the contexts in which reversion may be more significant.

4. The interpretation that bipolar segregation is required for recombinant formation is somewhat unclear (lines 396–413). Do the authors propose that chromosome breakage arises from spindle-dependent tension during anaphase, from cytokinesis-associated forces during mitotic exit, or from another mechanism? One possibility not made explicit in the text, but consistent with chromatin bridge literature, is that breakage occurs when DNA persists within the bud neck during cytokinesis, independent of whether this positioning results from direct spindle attachment or indirect linkage-mediated displacement. The data presented (e.g. chromatin bridges and bud neck-associated DNA) appear equally consistent with a model in which spatial positioning at cytokinesis, rather than bipolar spindle forces per se, is the primary determinant of chromosome breakage. This interpretation is supported by prior work on dicentric chromosomes (PMID: 25644606, cited by the authors) and - perhaps even more relevant - on chromosomes with intermolecular linkages (PMID: 24286828, 27111841). This point would benefit from further clarification and discussion.

5. The proposed mechanism of linkage-mediated chromosome breakage is highly reminiscent of prior work showing that late-replicating regions (e.g. subtelomeres or rDNA) can remain unresolved into anaphase and incur damage upon cytokinesis in otherwise unperturbed cells. Notably, cytokinesis induces subtelomeric mutations in these cases (PMID: 32385287). This raises the possibility that the phenomena described here represent a chromosome-scale manifestation of previously described locus-specific replication/segregation defects. The manuscript would benefit from a more explicit integration of this literature, and clarification of what aspects of the mechanism are genuinely novel versus extensions of these established observations. In this context, the novelty appears to lie less in identifying cytokinesis-associated damage per se, and more in extending its consequences to chromosome-scale aneuploidy dynamics.

Reviewer #3: The manuscript "A unified model of aneuploid karyotype dynamics" addresses an important question in chromosome biology: how do aneuploidy formation rates, reversion rates, and fitness consequences interact to determine karyotypic stability in dividing populations? The authors develop a sophisticated dual-reporter system in budding yeast that enables systematic measurement of μA, μR, and ω across all 16 chromosomes in a unified genetic background, integrate these measurements into a forward simulation framework, and identify a subset of chromosomes whose reversion dynamics cannot be explained by canonical mutational models. The paper further proposes and provides evidence for an intermolecular linkage mechanism to explain these non-canonical dynamics, supported by whole-genome sequencing of recombinant clones and live-cell fluorescence microscopy.

The work represents a great technical achievement and makes genuine contributions to the comprehension of aneuploid population dynamics. The CAN1-VIO dual reporter system is elegant, the orthogonal fitness assays provide important cross-validation, and the live-cell microscopy directly visualizing chromatin bridges and chromosome fragmentation is the most compelling mechanistic data in the paper. The primary conclusion — that canonical reversion contributes negligibly to karyotypic stability for most chromosomes and that fitness is the dominant determinant of aneuploid population dynamics — is well supported by multiple independent measurement approaches.

However, some major and minor concerns must be addressed before the manuscript can be accepted. These range from an unacknowledged structural assumption in the computational framework, to the correlative rather than causal nature of the evidence for the intermolecular linkage mechanism, to the framing of the paper's scope and novelty. These concerns are detailed below.

MAJOR CONCERNS

1. The fflucsim Structural Assumptions

The authors present a thorough exploration of parameter space within fflucsim, pushing μR to values approaching 1 and demonstrating that even this extreme cannot recapitulate Chr12 dynamics. This is a genuinely strong result within the model's framework. However, a structural assumption of the simulator warrants explicit acknowledgment that is currently absent from the paper.

To my understand the fflucsim models population expansion as a well-mixed system, yet the monosome replating assay — from which the revertant frequency distributions being modeled are derived — is performed on solid agar medium where spatially clustered progeny from a single early reversion event remain physically grouped. For chromosomes with extreme fitness costs such as Chr12 (ω≈0.25), a single reversion event occurring within the first few divisions of a slow-growing monosomic colony could generate a spatially expanding revertant subclone that dominates the countable colony area by day 2 through fitness-driven spatial expansion rather than through genuinely elevated μR. No combination of μR and ω within a well-mixed population model can reproduce this dynamic because the model is structurally incapable of representing spatial heterogeneity, regardless of what parameter values are explored.

The directly testing this concern is non-trivial given the constraints of the violacein-based detection system. Performing the assay in liquid culture would eliminate spatial structure but destroy the pigment-based readout, and a time course approach on solid medium might be confounded by the inherent lag between reversion event occurrence and sufficient violacein accumulation for visual detection. The paper's existing data provides partial mitigation — the liquid-grown fluctuation test populations and the WGS characterization of individual clones are both independent of solid medium geometry and broadly consistent with the replating assay results. Nevertheless, the well-mixed assumption represents an unacknowledged limitation of the analytical framework that should be explicitly discussed.

The authors should address this limitation in the Discussion and note that fully resolving it would require development of a detection system that does not depend on pigment accumulation on solid medium. Until such a system is developed, the conclusion that Chr12 dynamics are incompatible with any canonical parameter combination must be framed with the caveat that the assay geometry itself introduces spatial heterogeneity that the model cannot capture.

2. Molecular identity of the linkages (Chr4 and Chr12)

No experiment demonstrates that disrupting linkage formation specifically reduces recombinant clone frequency for Chr4 and Chr12 while leaving stable chromosomes unaffected. The molecular species constituting the linkages — topological catenation, cohesin entrapment, unresolved recombination intermediates — is never identified, which limits the mechanistic conclusions and prevents connecting the proposed mechanism to specific molecular perturbations.

Deleting FOB1 in the CAN1-VIO Chr12 strain and repeat the monosome replating assay and WGS characterization would directly tests whether rDNA recombination intermediates are the linkage species driving Chr12 non-canonical reversion. Fob1 deletion specifically reduces rDNA recombination without affecting transcription or copy number. The prediction is falsifiable: fob1Δ should reduce recombinant clone frequency for Chr12 — particularly the rDNA breakpoint class in Figure S7 — without affecting Chr1 or Chr2 reversion dynamics. This would be a more direct test for one of the main hypothesis generated from this work data.

Another direct assay would be to engineer a truncated rDNA array of defined size inserted at a defined locus on Chr3, a chromosome showing clean canonical monosomic behavior. Introduce this modified Chr3 into the CAN1-VIO system and measure μR, recombinant clone frequency, and breakpoint patterns by WGS, comparing to unmodified Chr3. This experiment directly tests sufficiency — whether rDNA presence alone is sufficient to convert a stable chromosome into a non-canonical reverter — completely decoupled from Chr12 identity, centromere sequence, and gene content. A positive result would also have significant broader implications for understanding instability of any chromosome bearing large repetitive arrays, including human acrocentric chromosomes.

3. The GAL1p-CEN Checkpoint Issue

The authors explicitly acknowledge the artificial biology: "Although the URA3-GAL1p-CEN system generates a mitotic scenario that would rarely occur in wild-type cells—because an unattached chromatid pair typically activates a mitotic checkpoint-mediated cell cycle arrest—it constituted a valuable tool to further probe the validity of the intermolecular linkage model."

This acknowledgment is appropriate but the checkpoint escape mechanism is never characterized. In Figure 7, 28.3% of cells show normal disjunction, 55% show induced nondisjunction, and approximately 17% show alternative segregation — meaning a substantial fraction of cells with inactivated centromeres escape checkpoint arrest and complete division. Whether checkpoint escape rates vary across chromosomes, which would bias which chromosomes produce recombinant FOA resistant clones, is never addressed. Additionally the uracil starvation pregrowth required by the experimental design could independently influence chromosome segregation fidelity through replication stress, a confound that is not discussed. An assay to measure checkpoint activation duration and/or escape frequency per chromosome would be important to strength further statements. If checkpoint escape rates differ significantly across chromosomes — particularly if Chr12 and Chr4 show higher escape rates than Chr1 and Chr3 — this would indicate that differential checkpoint biology contributes to the observed differences in recombinant clone recovery, requiring reinterpretation of the GAL1p-CEN results.

4. Fitness Measurement Discordance Between Assays

Figure 2A shows systematic differences between induced and preselected ω values. The exclusion criterion for the preselected assay is stated: "this approach was restricted to monosomes that consistently exhibited undetectable revertant frequencies." This justification is reasonable — preselected monosomes cannot be measured for fast-reverting chromosomes because the populations are already contaminated with revertants by the time of measurement.However, the paper uses induced ω values for these fast-reverting chromosomes in the fflucsim modeling without validating that the induced assay accurately captures true monosomic fitness for chromosomes like Chr12 where alternative segregation events occur at high rates during the induction period. Cells surviving galactose induction and 5-FOA selection for fast-reverting chromosomes may not be representative monosomes but rather cells that underwent alternative segregation events, potentially reporting fitness values that reflect a mixed population rather than pure monosomes.

5. Replating Assay Temporal Asymmetry

The Methods state: "After 48hrs, dark revertant colonies were quantified; after 96hrs, light monosomic colonies were quantified." The fitness correction applied through mlemur is described but the specific appropriateness of mlemur's correction for solid-medium colony counting — as opposed to the liquid culture fluctuation tests for which it was designed — is never discussed.

Looking at Supplemental Table 5, individual replicate revertant frequencies for Chr4 range from 0% to 97%, and for Chr13 show enormous variance. This variance itself suggests that the fitness correction may be insufficient for the solid-medium geometry, where spatial structure creates non-linear expansion dynamics that the population-level correction cannot capture.

The temporal asymmetry and fitness correction appropriateness for solid-medium colony counting are not addressed, and the high variance in replicate revertant frequencies for intermediate chromosomes suggests the correction may be insufficient. The Methods section should explicitly acknowledge that mlemur's fitness correction assumes well-mixed population dynamics and discuss whether this assumption holds for the solid-medium replating assay geometry. The Discussion should acknowledge that high variance in revertant frequencies across replicates for chromosomes like Chr4 and Chr13 could reflect either genuine Luria-Delbrück jackpot dynamics or spatial expansion artifacts, and that these explanations are not fully distinguishable with the current experimental system.

6. WGS Sample Sizes

Supplemental Table 2 reveals that for several chromosomes, as few as 5 light CAN^R clones were sequenced. The core Chr4 finding — 8/10 dark CANr clones showing pericentromeric breakpoints — is presented without confidence intervals or statistical comparison to other chromosomes. The paper never calculates whether this sample size provides sufficient power to detect the claimed frequency difference from other chromosomes or from what would be expected by chance.

The authors do provide a larger dataset for Chr12 specifically through the single-cassette strain experiment in Figure 5F-H, sequencing 45 dark CAN^R clones, which is more reassuring for that specific claim. But the generalization to other chromosomes remains statistically underpowered.

7. The rDNA Linkage Hypothesis

The rDNA hypothesis is introduced as an observation — 22% of Chr12 FOAR clones show rDNA breakpoints — and elevated to a general mechanism in the Discussion. The authors acknowledge this is a hypothesis: "Our work prompts the hypothesis that even in unperturbed wild type cells, persistent linkages existing within the ~1Mb rDNA array facilitate high-frequency chromosome nondisjunction events."

The fact that 78% of Chr12 FOAR clones did not show rDNA breakpoints is never explicitly addressed as a challenge to the rDNA linkage model being general for Chr12. The authors propose that rDNA-mediated events resolving through repetitive region breakage would appear as typical UPDs indistinguishable from canonical reversion — which is a reasonable explanation but is not tested. The proximity of the GAL1p-CEN12 cassette to the rDNA in the strain construction raises the possibility that rDNA breakpoints are an assay architecture artifact, which is also never formally excluded.

8- Chr6 and Chr7 Exclusion Requires Explicit Justification

The paper claims to provide a systematic analysis of aneuploidy dynamics across all 16 yeast chromosomes, yet Chr6 and Chr7 are effectively excluded from the quantitative rate analysis without explicit criteria stated in the main text. The authors correctly identify that these chromosomes induce chromosomal instability through ACT1/TUB2 dosage imbalance, which violates the stable mutation rate assumption of fluctuation analysis. However, this exclusion justification appears only implicitly through the mechanistic discussion of CIN, never as an explicit statement of scope limitation.

More importantly, the Methods note that VIO cassette integration failed repeatedly for several strains including those used for Chr6 and Chr7 — a significant technical limitation mentioned only in a footnote without connection to the main results narrative. The paper should explicitly state in the Results which chromosomes are excluded from which analyses and why, rather than allowing readers to piece this together from scattered statements across Methods and supplementary materials.

I also noted that whether the CIN phenotype of Chr6 and Chr7 is mechanistically related to the non-canonical reversion observed in Chr4 and Chr12 is an interesting question the paper raises but does not address. Given the authors' proposed intermolecular linkage mechanism, it is worth considering whether the elevated nondisjunction rates induced by Chr6 aneuploidy could themselves generate linkage-mediated events on other chromosomes — a possibility that would have implications for interpreting the unselected aneuploidy burden observed in Figure S1. Without the need to perform a new experiment the author could perform a lower-resolution analysis that might be achievable with the existing deposited sequencing data to specifically, test whether the midpoints of unselected LOH events in Chr6 and Chr7 light CANr clones show statistically significant enrichment near centromeres relative to random chromosomal positions. This analysis would either provide preliminary support for linkage-mediated events in a CIN context or appropriately conclude that the existing data lacks sufficient resolution to address the question. Either outcome is informative and should be reported as a supplementary analysis.

Additionally, the Discussion should include a brief paragraph acknowledging that whether Chr6-induced CIN generates linkage-mediated events as a mechanistic consequence of spindle disruption remains an open question, identifying it as an important direction for future work. This would substantially improve the paper's mechanistic coherence and honestly situate the Chr6/Chr7 observations within the broader intermolecular linkage framework the paper proposes.

MINOR CONCERNS

1.Yeast-to-Human Extrapolation

The Abstract states the work "expands our perspective of the diverse, and chromosome-specific mutational mechanisms shaping genome architecture" in a context framed around human disease. In the discussion section we can see the following statement "Discerning the underlying basis for this variance warrants further study, as doing so is certain to clarify central principles of genome stability and refine current paradigms of aneuploidy-driven disease risk". But I believe it is important to address in the paper that there are fundamental difference between S. cerevisiae point centromeres (~125bp, single CENP-A nucleosome) and human regional centromeres (megabases, complex chromatin) acknowledged as a limitation on extrapolation. The intermolecular linkage mechanism proposed here specifically involves rDNA on Chr12 and centromere-proximal recombination — both features that are organized entirely differently in humans. The word "certain" in "certain to clarify" is an overstatement that no data in the paper supports.

2. Genetic Background Effects

Supplemental Table 1 shows Chr5 diploid strain is constructed as "(LRH279 x LRH202)" where LRH279 is in JAY291 background while most other strains are S288c. Several strains also show mixed parentage.

The Methods state only: "Strain construction was performed using standard transformation, crossing, and sporulation procedures" with no discussion of background standardization.

What is missing: The paper never discusses whether the JAY291 vs S288c background difference for Chr5 could contribute to its apparently distinct behavior in the fitness and rate analyses. JAY291 is a Brazilian bioethanol strain with substantially different genome content, ploidy history, and stress response compared to S288c. Given that Chr5 shows one of the higher μA values (Figure 3A) and relatively normal reversion dynamics, the possibility that background effects contribute to this pattern is never considered.

3. The "Unified Model" Framing:

The title claims "A unified model of aneuploid karyotype dynamics" and the author states: "Collectively, this work establishes a unified model of aneuploid population genetics"

But the author also acknowledges: "The genetic systems used in this study allowed detection of but a subset of outcomes predicted by our models of linkage-mediated missegregation, suggesting that we have substantially underestimated the true incidence of this mutational mechanism"

And: "Discerning the underlying basis for this variance warrants further study" regarding why some chromosomes are subject to linkage-mediated instability more than others.

A truly unified model would provide quantitative criteria to predict which chromosomes undergo canonical versus non-canonical reversion. Instead, the classification is entirely post-hoc — chromosomes are labeled non-canonical because their empirical data deviates from model predictions, not because any feature of the chromosome predicts non-canonical behavior prospectively. The Discussion speculates about centromere sequence variation, nuclear organization, and rDNA content as potential predictors, but none of these are tested. The model describes two classes of chromosome behavior but cannot predict class membership from first principles, which substantially limits its utility as a "unified" framework. So please re-consider the use of "unified framework".

**********

**Have all data underlying the figures and results presented in the manuscript been provided?**

Large-scale datasets should be made available via a public repository as described in the *PLOS Genetics*   data availability policy, and numerical data that underlies graphs or summary statistics should be provided in spreadsheet form as supporting information.

Reviewer #1: Yes

Reviewer #2: Yes

Reviewer #3: Yes

**********

PLOS authors have the option to publish the peer review history of their article (what does this mean?). If published, this will include your full peer review and any attached files.

If you choose “no”, your identity will remain anonymous but your review may still be made public.

**Do you want your identity to be public for this peer review?**   For information about this choice, including consent withdrawal, please see our Privacy Policy.

Reviewer #1: No

Reviewer #2: No

Reviewer #3: No

[NOTE: If reviewer comments were submitted as an attachment file, they will be attached to this email and accessible via the submission site. Please log into your account, locate the manuscript record, and check for the action link "View Attachments". If this link does not appear, there are no attachment files.]

**Figure resubmission:**

While revising your submission, we strongly recommend that you use PLOS’s NAAS tool (https://ngplosjournals.pagemajik.ai/artanalysis) to test your figure files. NAAS can convert your figure files to the TIFF file type and meet basic requirements (such as print size, resolution), or provide you with a report on issues that do not meet our requirements and that NAAS cannot fix.

After uploading your figures to PLOS’s NAAS tool - https://ngplosjournals.pagemajik.ai/artanalysis, NAAS will process the files provided and display the results in the "Uploaded Files" section of the page as the processing is complete. If the uploaded figures meet our requirements (or NAAS is able to fix the files to meet our requirements), the figure will be marked as "fixed" above. If NAAS is unable to fix the files, a red "failed" label will appear above. When NAAS has confirmed that the figure files meet our requirements, please download the file via the download option, and include these NAAS processed figure files when submitting your revised manuscript.

**Reproducibility:**

To enhance the reproducibility of your results, we recommend that authors of applicable studies deposit laboratory protocols in protocols.io, where a protocol can be assigned its own identifier (DOI) such that it can be cited independently in the future. Additionally, PLOS ONE offers an option to publish peer-reviewed clinical study protocols. Read more information on sharing protocols at https://plos.org/protocols?utm_medium=editorial-email&utm_source=authorletters&utm_campaign=protocols

# Author comment: Author response to Decision Letter 1

**DOI:** 10.1371/journal.pgen.1012210.r002

14 May 2026

Attachment Submitted filename: Response to Reviewers.pdf

# Peer review: Decision Letter 1

**DOI:** 10.1371/journal.pgen.1012210.r003

**Authors:** Kent W. Hunter, Soni Lacefield

8 Jun 2026

Dear Dr Heasley,

We are pleased to inform you that your manuscript entitled "Towards a unified model of aneuploid karyotype dynamics" has been editorially accepted for publication in PLOS Genetics. Congratulations!

Before your submission can be formally accepted and sent to production you will need to complete our formatting changes, which you will receive in a follow up email. Please be aware that it may take several days for you to receive this email; during this time no action is required by you. Please note: the accept date on your published article will reflect the date of this provisional acceptance, but your manuscript will not be scheduled for publication until the required changes have been made.

Once your paper is formally accepted, an uncorrected proof of your manuscript will be published online ahead of the final version, unless you’ve already opted out via the online submission form. If, for any reason, you do not want an earlier version of your manuscript published online or are unsure if you have already indicated as such, please let the journal staff know immediately at plosgenetics@plos.org.

In the meantime, please log into Editorial Manager at https://www.editorialmanager.com/pgenetics/, click the "Update My Information" link at the top of the page, and update your user information to ensure an efficient production and billing process. Note that PLOS requires an ORCID iD for all corresponding authors. Therefore, please ensure that you have an ORCID iD and that it is validated in Editorial Manager. To do this, go to ‘Update my Information’ (in the upper left-hand corner of the main menu), and click on the Fetch/Validate link next to the ORCID field.  This will take you to the ORCID site and allow you to create a new iD or authenticate a pre-existing iD in Editorial Manager.

If you have a press-related query, or would like to know about making your underlying data available (as you will be aware, this is required for publication), please see the end of this email. If your institution or institutions have a press office, please notify them about your upcoming article at this point, to enable them to help maximise its impact. Inform journal staff as soon as possible if you are preparing a press release for your article and need a publication date.

Thank you again for supporting open-access publishing; we are looking forward to publishing your work in PLOS Genetics!

Yours sincerely,

Soni Lacefield, PhD

Academic Editor

PLOS Genetics

Kent Hunter

Section Editor

PLOS Genetics

Aimée Dudley

Editor-in-Chief

PLOS Genetics

Anne Goriely

Editor-in-Chief

PLOS Genetics

www.plosgenetics.org

BlueSky: @plos.bsky.social

----------------------------------------------------

Reviewer's Responses to Questions

**Comments to the Authors:**

**Please note here if the review is uploaded as an attachment.**

Reviewer #1: I think the authors have thoughtfully addressed the reviewers' comments, and the revised manuscript is an excellent contribution.

Reviewer #2: The authors have satisfactorily addressed my comments and have improved the manuscript accordingly. In particular, they performed the additional chromosome-arm-length analysis, expanded the discussion of chromosome-specific reversion dynamics, and better integrated their findings with the existing literature on chromatin bridges and cytokinesis-associated chromosome breakage. I appreciate the authors’ careful revisions and would like to congratulate them on a very interesting and thoughtfully executed study.

**********

**Have all data underlying the figures and results presented in the manuscript been provided?**

Large-scale datasets should be made available via a public repository as described in the *PLOS Genetics*   data availability policy, and numerical data that underlies graphs or summary statistics should be provided in spreadsheet form as supporting information.

Reviewer #1: Yes

Reviewer #2: Yes

**********

PLOS authors have the option to publish the peer review history of their article (what does this mean?). If published, this will include your full peer review and any attached files.

If you choose “no”, your identity will remain anonymous but your review may still be made public.

**Do you want your identity to be public for this peer review?**   For information about this choice, including consent withdrawal, please see our Privacy Policy.

Reviewer #1: No

Reviewer #2: No

----------------------------------------------------

**Data Deposition**

If you have submitted a Research Article or Front Matter that has associated data that are not suitable for deposition in a subject-specific public repository (such as GenBank or ArrayExpress), one way to make that data available is to deposit it in the Dryad Digital Repository. As you may recall, we ask all authors to agree to make data available; this is one way to achieve that. A full list of recommended repositories can be found on our website.

The following link will take you to the Dryad record for your article, so you won't have to re‐enter its bibliographic information, and can upload your files directly:

http://datadryad.org/submit?journalID=pgenetics&manu=PGENETICS-D-26-00199R1

More information about depositing data in Dryad is available at http://www.datadryad.org/depositing. If you experience any difficulties in submitting your data, please contact help@datadryad.org for support.

Additionally, please be aware that our data availability policy requires that all numerical data underlying display items are included with the submission, and you will need to provide this before we can formally accept your manuscript, if not already present.

----------------------------------------------------

**Press Queries**

If you or your institution will be preparing press materials for this manuscript, or if you need to know your paper's publication date for media purposes, please inform the journal staff as soon as possible so that your submission can be scheduled accordingly. Your manuscript will remain under a strict press embargo until the publication date and time. This means an early version of your manuscript will not be published ahead of your final version. PLOS Genetics may also choose to issue a press release for your article. If there's anything the journal should know or you'd like more information, please get in touch via plosgenetics@plos.org.

# Editor report: Acceptance letter

**DOI:** 10.1371/journal.pgen.1012210.r004

**Authors:** Kent W. Hunter, Soni Lacefield

PGENETICS-D-26-00199R1

Towards a unified model of aneuploid karyotype dynamics

Dear Dr Heasley,

We are pleased to inform you that your manuscript entitled "

Towards a unified model of aneuploid karyotype dynamics" has been formally accepted for publication in PLOS Genetics! Your manuscript is now with our production department and you will be notified of the publication date in due course.

The corresponding author will soon be receiving a typeset proof for review, to ensure errors have not been introduced during production. Please review the PDF proof of your manuscript carefully, as this is the last chance to correct any errors. Please note that major changes, or those which affect the scientific understanding of the work, will likely cause delays to the publication date of your manuscript.

Soon after your final files are uploaded, unless you have opted out or your manuscript is a front-matter piece, the early version of your manuscript will be published online. The date of the early version will be your article's publication date. The final article will be published to the same URL, and all versions of the paper will be accessible to readers.

For Research Articles, you will receive an invoice from PLOS for your publication fee after your manuscript has reached the completed accept phase. If you receive an email requesting payment before acceptance or for any other service, this may be a phishing scheme. Learn how to identify phishing emails and protect your accounts at https://explore.plos.org/phishing.

Thank you again for supporting PLOS Genetics and open-access publishing. We are looking forward to publishing your work!

With kind regards,

Anita Estes

PLOS Genetics

On behalf of:

The PLOS Genetics Team

Carlyle House, Carlyle Road, Cambridge CB4 3DN | United Kingdom

plosgenetics@plos.org | +44 (0) 1223-442823

plosgenetics.org | Twitter: @PLOSGenetics
